# A novel quinine derivative as a PIM-1 kinase inhibitor induces apoptosis via mitochondrial depolarization with selective cytotoxicity in acute lymphoblastic leukemia cells

**DOI:** 10.3389/fphar.2026.1799674

**Published:** 2026-04-21

**Authors:** Viviana Donoso-Bustamante, Edison H. Osorio, Gabriela C. Torres, José A. López-Saenz, Renato J. Aguilera, Denisse A. Gutiérrez, Armando Varela-Ramírez, Yeray A. Rodríguez-Núñez, Javier Echeverría

**Affiliations:** 1 Laboratorio de Síntesis y Reactividad de Compuestos Orgánicos, Departamento de Química, Facultad de Ciencias Exactas, Universidad Andrés Bello, Santiago, Chile; 2 CBATA, Departamento de Ciencias Básicas y Áreas Comunes, Tecnológico de Antioquia Institución Universitaria-TdeA, Medellín, Colombia; 3 Department of Biological Sciences, Border Biomedical Research Center, The University of Texas at El Paso, El Paso, TX, United States; 4 Departamento de Ciencias del Ambiente, Facultad de Química y Biología, Universidad de Santiago de Chile, Santiago, Chile

**Keywords:** acute lymphoblastic leukemia, apoptosis, mitochondrial depolarization, PIM-1 kinase, quinine derivatives

## Abstract

**Background:**

Acute lymphoblastic leukemia (ALL) remains a major therapeutic challenge, particularly in adult patients with poor tolerance to intensive chemotherapy, underscoring the need for novel and selective antileukemic agents that target more specific pharmacological targets and contribute to the development of new, less invasive therapeutic interventions. Quinine, a natural alkaloid, and its derivatives have demonstrated promising anticancer properties that could contribute to this development.

**Purpose:**

In this study, a series of lipophilic and amphiphilic quinine derivatives were synthesized and structurally characterized, and the *in vitro* cytotoxicity was evaluated against CEM and Jurkat leukemia cell lines and non-cancerous fibroblasts (Hs-27). To elucidate the mechanism of action, analyses revealed a strong affinity of the compounds for PIM-1 kinase, a protein overexpressed in most cases of ALL.

**Results and discussion:**

Among the 26 synthesized compounds, compound **22** exhibited the highest cytotoxic potency and selectivity toward CEM leukemia cells (CC_50_ = 2.25 ± 0.03 µM; SCI = 4.5). Mechanistic studies demonstrated that compound **22** induces apoptosis by externalizing phosphatidylserine, depolarizing the mitochondrial membrane, activating caspase-3/7, and causing DNA fragmentation, without causing cell-cycle arrest. Molecular docking and molecular dynamics simulations revealed strong, stable interactions between the most active compounds, particularly compound **22**, and PIM-1 kinase, supported by favorable MM/GBSA binding free energy values.

**Conclusion:**

Overall, these findings identify compound **22** as a promising PIM-1 kinase–targeting antileukemic candidate.

## 1 Introduction

Cancer remains one of the leading causes of death worldwide. In 2022, approximately 20 million new cancer cases and nearly 10 million cancer-related deaths were reported. It is projected that by 2050, more than 35 million new cases will occur, representing a 77% increase compared with 2022 estimates (Bray et al., 2024). In Chile, more than 53,000 cancer cases were diagnosed in 2018, according to data from the Global Cancer Observatory (GLOBOCAN) (Villalobos et al., 2023). Also in Chile, mortality statistics from the Department of Health Statistics and Information (DEIS) identify cancer as the second leading cause of death in the country (Parra-Soto et al., 2020).

Acute lymphoblastic leukemia (ALL) is a malignant hematologic disorder characterized by the uncontrolled proliferation of immature lymphoid cells that infiltrate the bone marrow, peripheral blood, and extramedullary sites (Pagliaro et al., 2024). This disease predominantly affects children, who generally exhibit better therapeutic outcomes and lower mortality rates (Wu et al., 2025). In contrast, adult patients show poorer prognoses, mainly due to intolerance to intensive chemotherapy regimens (Pan et al., 2025). These observations highlight the urgent need to develop new antileukemic agents with greater selectivity and efficacy, particularly for adult patients with ALL.

Quinoline-based compounds act as potent anticancer agents by targeting multiple pathways, including apoptosis induction, cell cycle arrest, and angiogenesis inhibition (El-Miligy et al., 2023; Chawla and Vaidya, 2024; Saxena et al., 2024). Quinine is a cinchona alkaloid, cinchonidine, in which the hydrogen at the 6-position of the quinoline ring is substituted by methoxy (Hariyanti et al., 2022) that exhibits a broad spectrum of pharmacological activities (Santos and Rao, 1998; Große et al., 2021; Kato and Oshima, 2023; Mohammadi and Rezaei Niaraki, 2023; Basrani et al., 2025; Xiong et al., 2025). It is currently used to treat severe malaria (Aymard, 2019; Antika et al., 2020). Quinine and its derivatives have attracted increasing attention as potential anticancer agents. Several studies have demonstrated that quinine exerts cytotoxic, antiproliferative, and proapoptotic effects by elevating intracellular reactive oxygen species (ROS) levels in various tumor cell lines (Averill-Bates, 2024). In Hep-2 cells, quinine induces mitochondrial membrane depolarization and DNA damage (Krishnaveni and Suresh, 2015). In contrast, in HeLa and A549 cells, it suppresses AKT activation, a key signaling pathway involved in cell survival and tumor progression (Liu et al., 2016). Moreover, in the K562/ADM leukemia cell line, quinine has been shown to reverse doxorubicin resistance (Solary et al., 1991). Structural modification of quinine has also led to derivatives with enhanced antitumor activity, such as quinine-chalcone hybrids that have apoptotic effects on EBC-1 cells by inhibiting P-glycoprotein (Takács et al., 2025) and thiourea-, squaramide-, and thiosquaramide-based derivatives exhibiting cytotoxicity in MES-SA cells (Pósa et al., 2022). In addition, analogues bearing thiourea, urea, or sulfonamide moieties have been reported to inhibit the enzyme IDO1, which plays a central role in tumor immune evasion, thereby representing a promising strategy for developing new anticancer therapies (Pradhan et al., 2021).

The design of lipophilic and amphiphilic derivatives via structural modification represents a promising strategy for developing new, more effective anticancer agents with improved pharmacokinetic and pharmacodynamic profiles. Increasing lipophilicity enhances the stability, bioavailability, and passive diffusion of compounds, promoting their selective accumulation in tumor tissues characterized by elevated fatty acid uptake (Fattahi et al., 2020). In this context, the conjugation of gemcitabine with linoleic acid via an amide bond has been shown to increase metabolic stability and enhance *in vitro* cytotoxicity against L3.6pl and BXPC-3 cells compared with the parent drug (Huang et al., 2022). Furthermore, the design of compounds with cationic and amphiphilic characteristics enhances their interaction with biological membranes, thereby contributing to the development of new agents with greater therapeutic efficacy (Kawai et al., 2021).

Several studies have identified casein kinase 2 (CK2) and proviral integration site for Moloney murine leukemia virus (PIM) protein kinases as key mediators of signal transduction pathways, making them among the most promising targets for potential therapeutic targets in many cancers, particularly prostate, breast, lung cancers, and in hematological cancers (Ghani et al., 2024; Sharma et al., 2024). On the other hand, some studies have identified PIM kinases as potential therapeutic targets in T-cell acute lymphoblastic leukemia (T-ALL) (Chawla and Vaidya, 2024; Sharma et al., 2025). Gene expression profiling revealed that PIM-1 is overexpressed in the majority of early T-cell precursor (ETP)-ALL, as well as in a small subset of non-ETP ALL. Conversely, scientific evidence demonstrates a correlation between CK2 and the pathogenesis of diverse hematological malignancies, including leukemia (Cai et al., 2025). Nevertheless, there is a paucity of congruence between clinical outcomes and these findings.

While quinine and its derivatives have demonstrated general anticancer properties, and lipophilic modification is a known strategy to enhance drug delivery, the potential of rationally designed, amphiphilic quaternary ammonium quinine derivatives to act as targeted inhibitors of PIM-1 kinase in ALL remains unexplored. Furthermore, the precise molecular mechanism by which such derivatives might induce cell death—specifically, whether they trigger the intrinsic apoptosis pathway downstream of PIM-1 inhibition—has not been elucidated. This study addresses this gap by synthesizing a novel series of such compounds and systematically investigating their selective anti-leukemic mechanism of action.

In this study, the effects of a series of lipophilic and amphiphilic cationic quinine derivatives were evaluated on CEM cells of acute lymphoblastic leukemia. The compound exhibiting the highest cytotoxicity against this cell line was selected for further *in vitro* assays to elucidate its potential mechanism of action. In addition, molecular dynamics simulations were performed for the most active and selective derivatives against the cancerous CEM and non-cancerous Hs-27 cell lines to gain insight into their interactions with potential protein targets. These simulations revealed a strong affinity of the compounds for the PIM-1 kinase, a protein that is overexpressed in most cases of ALL. Overall, these findings suggest that the studied derivatives are promising anticancer agents that may act by inhibiting PIM-1 kinase.

## 2 Materials and methods

### 2.1 Chemicals and reagents

All reagents were obtained from Sigma-Aldrich Corp. (St. Louis, MO, USA). Compounds 2–26 were synthesized according to the procedures previously described (Echeverría et al., 2017; Mukusheva et al., 2022). Analytical TLC was used for compound identification and purification on 5-cm-long plates coated with 0.25 mm thick Merck silica gel 60F-254, visualized under UV light. Column chromatography was performed using Silicycle silica gel 60 (0.040–0.063 mm) and analytical-grade solvents. Melting points were measured using an electrothermal apparatus (Stuart Scientific, United Kingdom) in open capillaries and were uncorrected. Compound purification was performed by column chromatography on silica gel. Analytical grade solvents were used. ^1^H and ^13^C NMR spectra were acquired in CDCl_3_ on a Bruker Avance 400 NMR spectrometer at 400.13 MHz (^1^H) or 100.61 MHz (^13^C). Chemical shifts are reported in parts per million (ppm) downfield of TMS as an internal standard (δ scale), multiplicity [br = broad, s = singlet, d = doublet, t = triplet, q = quartet, ddd = doublet of doublet of doublets, m = multiplet] and coupling constants (*J*) in hertz (Hz). High-resolution mass spectra (HRMS) were obtained on a Bruker Compact Q-TOF MS (ESI/QTOF).

### 2.2 Synthesis

#### 2.2.1 General procedure for the synthesis of acylquinine derivatives, series 1 (2-14)

**FIGURE 1:**
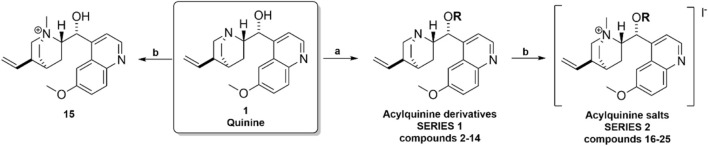
Synthesis of acylquinine derivatives (**2**–**14**) and acylquinine salts (**15**–**25**). (a) RCOCI, DMAP, DCM, 12h, 80°C; (b) MeOH, CHзl, 24h, rt. R: acetyl, propionyl, butyryl, pentanoyl, hexanoyl, octanoyl, nonanoyl, decanoyl, palmitoyl, oleoyl, cyclohexanoyl, benzoyl, and cinnamoyl.

The synthetic methodology for obtaining the studied compounds **2-14** of series **1** is shown in Figure 1. To obtain acylquinine derivatives, quinine (1 mmol) and 4-*N,N*-dimethylaminopyridine (DMAP, 1 mmol) in dichloromethane (10 mL) were added as solvent to a high-pressure tube, and the corresponding acyl chloride (2 mmol) was added to this mixture. The mixture was stirred at 80 °C for 12 h, then washed successively with a 6% sodium bicarbonate solution and distilled water. The organic phase was dried over anhydrous sodium sulfate, and the solvent was removed *in vacuo* using a rotary evaporator to afford the corresponding acyl derivative. The product was purified by chromatography using ethyl acetate/methanol (4:1) as the eluent (Echeverría et al., 2017; Mukusheva et al., 2022). This methodology was based on previously described research and has been optimized, reducing the reaction time from 24 h to 12 h using the high-pressure tube. The ^1^H and ^13^C NMR spectra of the compounds in series 1 and 2 are shown in Supplementary Figure S1-S45.

#### 2.2.2 Synthesis of (*R*)-(6-methoxyquinolin-4-yl) ((1*S*,2*S*,4*S*,5*R*)-5-vinylquinuclidin-2-yl) methyl acetate (2)

The general procedure was applied to quinine **1** and acetyl chloride, column flash chromatography on silica gel, allowed the isolation of 2, white solid (0.318 g, 56%); ^1^H NMR (CDCl_3_) δ: 8.74 (d, *J* = 4.4 Hz, 1H), 8.01 (d, *J* = 9.3Hz, 1H), 7.44 (d, *J* = 2.6 Hz, 1H), 7.33–7.40 (m, 2H), 6.49 (d, *J* = 7.4 Hz, 1H), 5.84 (ddd, *J* = 17.5, 10.3, 7.5 Hz, 1H), 5.07–4.95 (m, 2H) 3.96 (s, 3H), 3.37 (q, *J* = 8.1 Hz, 1H), 3.15–2.97 (m, 2H), 2.72–2.56 (m, 2H), 2.33–2.24 (m, 1H), 2.12 (s, 3H), 1.94–1.80 (m, 2H), 1.77–1.67 (m, 1H), 1.58–1.46 (m, 2H); ^13^C NMR (CDCl_3_) δ: 170.09, 157.93, 147.48, 144.83, 143.60, 141.76, 131.84, 127.07, 121.79, 118.94, 114.51, 101.52, 73.78, 59.08, 56.63, 55.66, 42.46, 39.73, 27.80, 27.58, 24.36, 21.09; HRMS *m/z*: calcd. For C_22_H_26_N_2_O_3_ [M]^+^ 367.2016 found 367.2030; m. p. 114.1 °C–115.3 °C (Molnár and Gilmour, 2016).

#### 2.2.3 Synthesis of (*R*)-(6-methoxyquinolin-4-yl) (1*S*,2*S*,4*S*,5*R*)-5-vinylquinuclidin-2-yl) methyl propionate (3)

The general procedure was applied to quinine **1** and propionyl chloride, column flash chromatography on silica gel, allowed the isolation of **3**, white solid (0.428 g, 73%); ^1^H NMR (CDCl_3_) δ: 8.73 (d, *J* = 4.5 Hz, 1H), 8.01 (d, *J* = 9.2 Hz, 1H), 7.45 (d, *J* = 2.7 Hz, 1H), 7.39–7.33 (m, 2H), 6.50 (d, *J* = 7.3 Hz, 1H), 5.84 (ddd, *J* = 17.3, 10.3, 7.3 Hz, 1H), 5.07–4.95 (m, 2H), 3.96 (s, 3H), 3.37 (q, *J* = 8.1 Hz, 1H), 3.16–3.00 (m, 2H), 2.72–2.57 (m, 2H), 2.45–2.36 (m, 2H), 2.31–2.23 (m, 1H), 1.95–1.82 (m, 2H), 1.77–1.67 (m, 1H), 1.60–1.48 (m, 2H), 1.15 (t, *J* = 7.5 Hz, 3H); ^13^C NMR (CDCl_3_) δ: 173.50, 157.92, 147.46, 144.82, 143.78, 141.76, 131.82, 127.08, 121.82, 118.91, 114.52, 101.50, 73.60, 59.17, 56.61, 55.65, 42.44, 39.73, 27.80, 27.59, 24.39, 9.06; HRMS *m/z*: calcd. For C_23_H_28_N_2_O_3_ [M]^+^ 381,2173 found 381.2182; m. p. 122.2 °C–123.6 °C.

#### 2.2.4 Synthesis of (*R*)-(6-methoxyquinolin-4-yl) (1*S*,2*S*,4*S*,5*R*)-5-vinylquinuclidin-2-yl) methyl butyrate (4)

The general procedure was applied to quinine **1** and butyryl chloride, column flash chromatography on silica gel, allowed the isolation of **4**, translucent oil (0.4213 g, 69%); ^1^H NMR (CDCl_3_) δ: 8.73 (d, *J* = 4.5 Hz, 1H), 8.02 (d, *J* = 9.2 Hz, 1H), 7.45 (d, *J* = 2.8 Hz, 1H), 7.31–7.41 (m, 2H), 6.52 (d, *J* = 7.0 Hz, 1H), 5.83 (ddd, *J* = 17.4, 10.4, 7.4 Hz, 1H), 5.10–4.94 (m, 2H), 3.96 (s, 3H), 3.37 (q, *J* = 7.9 Hz, 1H), 3.19–3.01 (m, 2H), 2.74–2.56 (m, 2H), 2.36 (t, *J* = 7.2 Hz, 2H), 2.33–2.24 (m, 1H), 1.91–1.81 (m, 2H), 1.79–1.50 (m, 5H), 0.92 (t, *J* = 7.4 Hz, 3H); ^13^C NMR (CDCl_3_) δ: 172.65, 157.98, 147.37, 144.76, 143.75, 141.61, 131.75, 127.02, 121.90, 118.81, 114.60, 101.45, 73.40, 59.03, 56.45, 55.69, 42.38, 39.61, 36.37, 27.68, 27.59, 24.20, 18.39, 13.67; HRMS *m/z*: calcd. For C_24_H_30_N_2_O_3_ [M]^+^ 395,2329 found 395.2338.

#### 2.2.5 Synthesis of (*R*)-(6-methoxyquinolin-4-yl) (1*S*,2*S*,4*S*,5*R*)-5-vinylquinuclidin-2-yl) methyl pentanoate (5)

The general procedure was applied to quinine **1** and pentanoyl chloride, column flash chromatography on silica gel, allowed the isolation of **5**, translucent oil (0.454 g, 72%); ^1^H NMR (CDCl_3_) δ: 8.73 (d, *J* = 4.5 Hz, 1H), 8.02 (d, *J* = 9.2 Hz, 1H), 7.45 (d, *J* = 2.7 Hz, 1H), 7.37 (dd, *J* = 9.2, 2.7 Hz, 1H), 7.34 (d, *J* = 4.6 Hz, 1H), 6.51 (d, *J* = 7.1 Hz, 1H), 5.82 (ddd, *J* = 17.5, 10.4, 7.4 Hz, 1H), 4.95–5.06 (m, 2H), 3.96 (s, 3H), 3.36 (q, *J* = 7.9 Hz, 1H), 3.18–3.00 (m, 2H), 2.74–2.57 (m, 2H), 2.37 (t, *J* = 7.7 Hz, 2H), 2.32–2.23 (m, 1H), 1.91–1.80 (m, 2H), 1.78–1.67 (m, 1H), 1.68–1.48 (m, 4H), 1.40–1.22 (m, 3H), 0.88 (t, *J* = 7.4 Hz, 3H); ^13^C NMR (CDCl_3_) δ: 172.82, 157.96, 147.39, 144.78, 143.76, 141.64, 131.77, 127.03, 121.88, 118.83, 114.58, 101.47, 73.43, 59.06, 56.48, 55.67, 42.39, 39.64, 34.18, 27.71, 27.59, 26.91, 24.24, 22.22, 13.64; HRMS *m/z*: calcd. For C_25_H_32_N_2_O_3_ [M]^+^ 409,2486 found 409.2504.

#### 2.2.6 Synthesis of (*R*)-(6-methoxyquinolin-4-yl) (1*S*,2*S*,4*S*,5*R*)-5-vinylquinuclidin-2-yl) methyl hexanoate (6)

The general procedure was applied to quinine **1** and hexanoyl chloride, column flash chromatography on silica gel, allowed the isolation of **6**, Translucent oil (0.471 g, 72%); ^1^H NMR (CDCl_3_) δ: 8.73 (d, *J* = 4.5 Hz, 1H), 8.01 (d, *J* = 9.1 Hz, 1H), 7.45 (d, *J* = 2.7 Hz, 1H), 7.39–7.33 (m, 2H), 6.51 (d, *J* = 7.1 Hz, 1H), 5.83 (ddd, *J* = 17.5, 10.4, 7.4 Hz, 1H), 5.08–4.95 (m, 2H), 3.96 (s, 3H), 3.37 (q, *J* = 8.0 Hz, 1H), 3.19–2.99 (m, 2H), 2.75–2.57 (m, 2H), 2.37 (t, *J* = 8.2 Hz, 2H), 2.33–2.14 (m, 1H), 1.92–1.82 (m, 2H), 1.79–1.68 (m, 1H), 1.67–1.48 (m, 4H), 1.36–1.19 (m, 4H), 0.85 (t, *J* = 6.9 Hz, 3H); ^13^C NMR (CDCl_3_) δ: 172.85, 157.94, 147.40, 147.33, 144.79, 143.76, 141.66, 131.77, 127.04, 121.86, 118.87, 114.56, 101.49, 73.47, 59.06, 56.51, 55.67, 42.40, 39.65, 34.43, 31.22, 27.72, 27.58, 24.56, 24.29, 22.25, 13.84; HRMS *m/z*: calcd. For C_26_H_34_N_2_O_3_ [M]^+^ 423.2642 found 423.2636.

#### 2.2.7 Synthesis of (*R*)-(6-methoxyquinolin-4-yl) (1*S*,2*S*,4*S*,5*R*)-5-vinylquinuclidin-2-yl) methyl octanoate (7)

The general procedure was applied to quinine **1** and octanoyl chloride, column flash chromatography on silica gel, allowed the isolation of **7**, translucent oil (0.575 g, 82%); ^1^H NMR (CDCl_3_) δ: 8.73 (d, *J* = 4.5 Hz, 1H), 8.01 (d, *J* = 9.2 Hz, 1H), 7.45 (d, *J* = 2.7 Hz, 1H), 7.32–7.39 (m, 2H), 6.52 (d, *J* = 7.0 Hz, 1H), 5.83 (ddd, *J* = 17.5, 10.4, 7.4 Hz, 1H), 5.06–4.96 (m, 2H), 3.96 (s, 3H), 3.37 (q, *J* = 7.8 Hz, 1H), 3.18–3.01 (m, 2H), 2.74–2.57 (m, 2H), 2.38 (t, *J* = 7.7 Hz, 2H), 2.34–2.24 (m, 1H), 1.93–1.81 (m, 2H), 1.80–1.68 (m, 1H), 1.68–1.50 (m, 4H), 1.34–1.15 (m, 8H), 0.86 (t, *J* = 6.9 Hz, 3H); ^13^C NMR (CDCl_3_) δ: 172.86, 157.94, 147.40, 144.81, 143.74, 141.66, 131.79, 127.05, 121.85, 118.89, 114.56, 101.49, 73.46, 59.07, 56.52, 55.66, 42.40, 39.65, 34.47, 31.60, 29.04, 28.86, 27.73, 27.58, 24.90, 24.32, 22.54, 14.03; HRMS *m/z*: calcd. For C_28_H_38_N_2_O_3_ [M]^+^ 451.2955 found 451.2953.

#### 2.2.8 Synthesis of (*R*)-(6-methoxyquinolin-4-yl) (1*S*,2*S*,4*S*,5*R*)-5-vinylquinuclidin-2-yl) methyl nonanoate (8)

The general procedure was applied to quinine **1** and nonanoyl chloride, column flash chromatography on silica gel, allowed the isolation of **8**, translucent oil (0.552 g, 77%); ^1^H NMR (CDCl_3_) δ: 8.73 (d, *J* = 4.5 Hz, 1H), 8.01 (d, *J* = 9.2 Hz, 1H), 7.45 (d, *J* = 2.7 Hz, 1H), 7.31–7.40 (m, 2H), 6.50 (d, *J* = 7.3 Hz, 1H), 5.84 (ddd, *J* = 17.4, 10.4, 7.4 Hz, 1H), 4.96–5.06 (m, 2H), 3.96 (s, 3H), 3.37 (q, *J* = 8.1 Hz, 1H), 3.17–3.00 (m, 2H), 2.72–2.56 (m, 2H), 2.37 (t, *J* = 7.6 Hz, 2H), 2.30–2.26 (m, 1H), 1.94–1.82 (m, 2H), 1.78–1.48 (m, 5H), 1.33–1.17 (m, 10 H), 0.87 (t, *J* = 6.9 Hz, 3H); ^13^C NMR (CDCl_3_) δ: 172.93, 157.91, 147.43, 144.81, 143.75, 141.72, 131.80, 127.07, 121.86, 118.92, 114.57, 101.45, 73.50, 59.07, 56.55, 55.66, 42.42, 39.69, 34.48, 31.77, 29.18, 29.10, 27.76, 27.57, 24.91, 24.40, 22.63, 14.10, 14.07; HRMS *m/z*: calcd. For C_29_H_40_N_2_O_3_ [M]^+^ 465.3112 found 465.3117.

#### 2.2.9 Synthesis of (*R*)-(6-methoxyquinolin-4-yl) (1*S*,2*S*,4*S*,5*R*)-5-vinylquinuclidin-2-yl) methyl decanoate (9)

The general procedure was applied to quinine **1** and decanoyl chloride, column flash chromatography on silica gel, allowed the isolation of **9**, translucent oil (0.289 g, 39%); ^1^H NMR (CDCl_3_) δ: 8.73 (d, *J* = 4.6 Hz, 1H), 8.02 (d, *J* = 9.2 Hz, 1H), 7.45 (d, *J* = 2.7 Hz, 1H), 7.38 (dd, *J* = 9.5, 2.5 Hz, 1H), 7.33 (d, *J* = 4.5 Hz, 2H), 6.55 (d, *J* = 6.5 Hz, 2H), 5.81 (ddd, *J* = 17.4, 10.4, 7.3 Hz, 1H), 4.96–5.08 (m, 2H), 3.97 (s, 3H), 3.36 (q, *J* = 7.8 Hz, 1H), 3.21–3.04 (m, 2H), 2.77–2.58 (m, 2H), 2.39 (t, *J* = 7.3 Hz, 2H), 2.35–2.27 (m, 1H), 1.91–1.70 (m, 3H), 1.69–1.53 (m, 4H), 1.33–1.15 (m, 12H), 0.87 (t, *J* = 6.8 Hz, 3H); ^13^C NMR (CDCl_3_) δ: 172.83, 157.99, 147.36, 144.74, 143.67, 141.51, 131.75, 126.98, 121.97, 118.79, 114.68, 101.37, 73.33, 58.96, 56.38, 55.72, 42.38, 39.55, 34.48, 31.85, 29.39, 29.23, 29.11, 27.60, 27.56, 24.90, 24.11, 22.66, 22.02, 14.13; HRMS *m/z*: calcd. For C_30_H_42_N_2_O_3_ [M]^+^ 479.3268 found 479.3274.

#### 2.2.10 Synthesis of (*R*)-(6-methoxyquinolin-4-yl) (1*S*,2*S*,4*S*,5*R*)-5-vinylquinuclidin-2-yl) methyl palmitate (10)

The general procedure was applied to quinine **1** and palmitoyl chloride, column flash chromatography on silica gel, allowed the isolation of **10**, light yellow oil (0.117 g, 14%); ^1^H NMR (CDCl_3_) δ: 8.73 (d, *J* = 4.5Hz, 1H), 8.01 (d, *J* = 9.2Hz, 1H), 7.45 (d, *J* = 2.7Hz, 1H), 7.37 (dd, *J* = 9.2, 2.7 Hz 1H), 7.33 (d, *J* = 4.6Hz, 1H), 6.54 (d, *J* = 6.8Hz, 1H), 5.82 (ddd, *J* = 17.4, 10.4, 7.4Hz, 1H), 4.95–5.06 (m-2H), 3.97 (s, 3H), 3.38 (dd, *J* = 16.2, 8.1Hz, 1H), 3.19–3.02 (m, 2H), 2.75–2.57 (m, 2H), 2.37 (t, *J* = 6.8Hz, 1H), 2.34–2.18 (m, 1H), 1.92–1.80 (m, 2H), 1.81–1.68 (m, 1H), 1.68–1.52 (m, 5H), 1.35–1.17 (m, 27H), 0.88 (t, *J* = 6.8 Hz, 3H); ^13^C NMR (CDCl_3_) δ: 172.83, 157.99, 147.38, 144.76, 143.64, 141.52, 131.78, 126.99, 121.96, 118.80, 114.70, 101.39, 73.32, 58.99, 56.41, 55.74, 42.41, 39.57, 34.49, 31.95, 29.72, 29.68, 29.65, 29.59, 29.46, 29.39, 29.25, 29.12, 27.61, 27.56, 24.91, 24.14, 22.72, 14.16; HRMS *m/z*: calcd. For C_36_H_54_N_2_O_3_ [M]^+^ 563.4207 found 563.4211.

#### 2.2.11 Synthesis of (*R*)-(6-methoxyquinolin-4-yl) (1*S*,2*S*,4*S*,5*R*)-5-vinylquinuclidin-2-yl) methyl (*E*)-octadec-9-enoate (11)

The general procedure was applied to quinine **1** and oleoyl chloride, column flash chromatography on silica gel, allowed the isolation of **11**, light yellow oil (0.518 g, 57%); ^1^H NMR (CDCl_3_) δ: 8.73 (d, *J* = 4.5 Hz, 1H), 8.01 (d, *J* = 9.2 Hz, 1H), 7.45 (d, *J* = 2.7 Hz, 1H), 7.37 (dd, *J* = 9.2, 2.7 Hz, 1H), 7.34 (d, *J* = 4.5 Hz, 1H), 6.52 (d, *J* = 7.0 Hz, 1H), 5.83 (ddd, *J* = 17.5, 10.4, 7.4 Hz, 1H), 5.41–5.27 (m, 2H), 5.04–4.96 (m, 2H), 3.96 (s, 3H), 3.37 (q, *J* = 7.8 Hz, 1H), 3.18–3.01 (m, 2H), 2.80–2.57 (m, 2H), 2.37 (t, *J* = 7.9 Hz, 2H), 2.34–2.18 (m, 1H), 2.09–1.93 (m, 4H), 1.92–1.81 (m, 2H), 1.79–1.69 (m, 1H), 1.67–1.49 (m, 5H), 1.38–1.18 (m, 22H), 0.88 (t, *J* = 6.8 Hz, 3H); ^13^C NMR (CDCl_3_) δ: 172.84, 157.96, 147.38, 144.75, 143.70, 141.59, 131.77, 130.05, 129.70, 127.01, 121.93, 118.83, 114.64, 101.39, 73.40, 58.99, 56.44, 55.70, 42.40, 39.61, 34.47, 31.92, 29.78, 29.67, 29.54, 29.34, 29.15, 29.08, 27.67, 27.57, 27.24, 27.15, 24.89, 24.22, 22.70, 14.15; HRMS *m/z*: calcd. For C_38_H_56_N_2_O_3_ [M]^+^ 589.4364 found 589.4367.

#### 2.2.12 Synthesis of (*R*)-(6-methoxyquinolin-4-yl) (1*S*,2*S*,4*S*,5*R*)-5-vinylquinuclidin-2-yl) methyl cyclohexanecarboxylate (12)

The general procedure was applied to quinine **1** and cyclohexanoyl chloride, column flash chromatography on silica gel, allowed the isolation of **12**, white solid (0.310 g, 46%); ^1^H NMR (CDCl_3_) δ: 8.73 (d, *J* = 4.5 Hz, 1H), 8.01 (d, *J* = 9.2 Hz, 1H), 7.44 (d, *J* = 2.7 Hz, 1H), 7.39–7.32 (m, 2H), 6.48 (d, *J* = 7.3 Hz, 1H), 5.84 (ddd, *J* = 17.4, 10.4, 7.5 Hz, 1H), 5.09–4.95 (m, 2H), 3.96 (s, 3H), 3.37 (q, *J* = 8.1 Hz, 1H), 3.14–2.99 (m, 2H), 2.72–2.55 (m, 2H), 2.36 (tt, *J* = 11.3, 3.6 Hz, 1H), 2.27 (d, *J* = 9.9 Hz, 1H), 1.99–1.83 (m, 4H), 1.59–1.19 (m, 7H); ^13^C NMR (CDCl_3_) δ: 175.07, 157.88, 147.48, 144.81, 143.97, 141.83, 131.81, 127.11, 121.89, 118.83, 114.55, 101.41, 73.23, 59.25, 56.58, 55.65, 43.34, 42.43, 39.77, 29.00, 28.89, 27.82, 27.60, 25.68, 25.41, 25.35, 24.50; HRMS *m/z*: calcd. For C_27_H_34_N_2_O_3_ [M]^+^ 435.2642 found 435.2655. m. p. 127.1 °C–127.9 °C.

#### 2.2.13 Synthesis of (*R*)-(6-methoxyquinolin-4-yl) (1*S*,2*S*,4*S*,5*R*)-5-vinylquinuclidin-2-yl) methyl benzoate (13)

The general procedure was applied to quinine **1** and palmitoyl chloride, column flash chromatography on silica gel, allowed the isolation of **13**, White solid (0.404 g, 61%); ^1^H NMR (CDCl_3_) δ: 8.72 (d, *J* = 4.5 Hz, 1H), 8.14–8.07 (m, 2H), 8.03 (d, *J* = 9.2 Hz, 1H), 7.64–7.58 (m, 1H), 7.54 (d, *J* = 2.7 Hz, 1H), 7.48 (t, *J* = 7.7 Hz, 2H), 7.36–7.44 (m, 2H), 6.83 (d, *J* = 6.0 Hz, 1H), 5.83 (ddd, *J* = 17.4, 10.4, 7.4 Hz, 1H), 5.08–4.97 (m, 2H), 3.96 (s, 3H), 3.51 (q, *J* = 8.6 Hz, 1H), 3.32–3.11 (m, 2H), 2.83–2.67 (m, 2H), 2.39–2.29 (m, 1H), 1.98–1.87 (m, 2H), 1.87–1.74 (m, 2H), 1.68–1.55 (m, 1H); ^13^C NMR (CDCl_3_) δ: 171.23, 165.43, 158.13, 147.41, 144.75, 143.48, 141.40, 133.57, 131.85, 131.63, 129.69, 128.70, 127.98, 126.82, 122.10, 118.50, 114.84, 101.28, 74.22, 59.21, 56.47, 55.76, 42.54, 39.50, 27.68, 27.64, 23.75; HRMS *m/z*: calcd. For C_27_H_28_N_2_O_3_ [M]^+^ 429.2173 found 429.2170; m. p. 115.5 °C–116.3 °C.

#### 2.2.14 Synthesis of (*R*)-(6-methoxyquinolin-4-yl) (1*S*,2*S*,4*S*,5R)-5-vinylquinuclidin-2-yl) methyl cinnamate (14)

The general procedure was applied to quinine **1** and palmitoyl chloride, column flash chromatography on silica gel, allowed the isolation of **14**, light yellow oil (0.219 g, 31%); ^1^H NMR (CDCl_3_) δ: 8.74 (d, *J* = 4.5 Hz, 1H), 8.02 (d, *J* = 9.2 Hz, 1H), 7.73 (d, *J* = 16.0 Hz, 1H), 7.56–7.48 (m, 3H), 7.35–7.44 (m, 5H), 6.65 (d, *J* = 6.9 Hz, 1H), 6.51 (d, *J* = 16.0 Hz, 1H), 5.85 (ddd, *J* = 17.5, 10.4, 7.4 Hz, 1H), 5.06–4.97 (m, 2H), 3.99 (s, 3H), 3.44 (q, *J* = 8.1 Hz, 1H), 3.24–3.02 (m, 2H), 2.76–2.59 (m, 2H), 2.76–2.59 (m, 2H), 1.79–1.57 (m, 6H); ^13^C NMR (CDCl_3_) δ: 166.04, 157.98, 147.52, 146.06, 144.81, 143.64, 141.74, 134.05, 131.85, 130.71, 128.98, 128.24, 127.06, 121.89, 118.80, 117.32, 114.59, 101.45, 73.74, 59.20, 56.69, 55.74, 42.58, 39.73, 27.84, 27.62, 24.19; HRMS *m/z*: calcd. For C_29_H_30_N_2_O_3_ [M]^+^ 455.2329 found 455.2337.

#### 2.2.15 General procedure for the synthesis of acylquinine salts, series 2 (15–26)

Compounds **15–26** were prepared by mixing the corresponding acylquinine derivatives (1 mmol) with methanol (10 mL) in a flask. Methyl iodide (1.5 mmol) was added to the solution, and the mixture was stirred at room temperature for 24 h. The solvent was removed *in vacuo* using a rotary evaporator, and the product was washed with diethyl ether to afford the respective salt (Joyce et al., 2016; McNeice et al., 2020). The acylquinine salts are described in Figure 1.

#### 2.2.16 Synthesis of (1*S*,2*S*,4*S*,5*R*)-2-(*R*)-hydroxy(6-methoxyquinolin-4-yl)methyl)-1-methyl-5-vinylquinuclidin-1-ium iodide (15)

Following the general procedure, quinine **1** reacts with methyl iodide in methanol, after washing obtain **15**, white solid (0.356 g, 33%); ^1^H NMR (CDCl_3_) δ: 8.77 (d, *J* = 4.5 Hz, 1H), 8.06 (d, *J* = 9.3 Hz, 1H), 7.74 (d, *J* = 4.6 Hz, 1H), 7.40 (dd, *J* = 9.3, 2.6 Hz, 1H), 7.04 (d, *J* = 2.7 Hz, 1H), 6.53 (d, *J* = 5.9 Hz, 1H), 5.55 (ddd, *J* = 17.1, 10.4, 6.7 Hz, 1H), 5.13–5.02 (m, 2H), 3.98 (s, 3H), 3.82 (s, 3H), 3.48 (q, *J* = 7.0 Hz, 1H), 3.37 (t, *J* = 8.9 Hz, 1H), 3.14–3.24 (m, 1H), 2.89 (s, 1H), 1.95–2.33 (m, 4H), 1.41 (dd, *J* = 13.6, 10.3 Hz, 1H), 1.21 (t, *J* = 7.0 Hz, 2H). Compound described previously (McNeice et al., 2020).

#### 2.2.17 Synthesis of (1*S*,2*S*,4*S*,5*R*)-2-(*R*)-(butyryloxy)(6-methoxyquinolin-4-yl)methyl)-1-methyl-5-vinylquinuclidin-1-ium iodide (16)

Following the general procedure, acylquinine **4** reacts with methyl iodide in methanol, after washing obtain **16**, light yellow solid (0.073 g, 27%); ^1^H NMR (CDCl_3_) δ: 8.79 (d, *J* = 4.5 Hz, 1H), 8.11 (d, *J* = 9.3 Hz, 1H), 7.48 (dd, *J* = 9.3, 2.5 Hz, 1H), 7.37 (d, *J* = 4.6 Hz, 1H), 7.10 (d, *J* = 1.9 Hz, 1H), 7.02 (d, *J* = 2.6 Hz, 1H), 5.64 (ddd, *J* = 17.0, 10.5, 6.4 Hz, 1H), 5.15 (dd, *J* = 17.1, 1.3 Hz, 2H), 4.91 (t, *J* = 11.6 Hz, 1H), 4.06 (s, 3H), 3.72 (s, 3H), 3.76–3.55 (m, 3H), 3.08 (s ancho, 1H), 2.56 (t, *J* = 7.5 Hz, 2H), 2.38–2.09 (m, 5H), 1.74 (sext, *J* = 7.5 Hz, 2H), 1.01 (t, *J* = 7.4 Hz, 3H); ^13^C NMR (101 MHz, CDCl_3_) δ: 171.39, 159.13, 147.12, 144.68, 137.98, 136.07, 132.83, 125.06, 122.22, 118.52, 118.45, 99.89, 66.96, 66.52, 65.88, 56.32, 54.85, 49.82, 37.44, 36.34, 26.21, 25.13, 22.03, 18.19, 13.69; HRMS *m/z*: Calcd. For C_25_H_33_N_2_O_3_ [M]^+^ 409.2486 found 409.2486; m. p. 70.8 °C–71.2 °C.

#### 2.2.18 Synthesis of (1*S*,2*S*,4*S*,5*R*)-2-(*R*)-(hexanoyloxy)(6-methoxyquinolin-4-yl)methyl)-1-methyl-5-vinylquinuclidin-1-ium iodide (17)

Following the general procedure, acylquinine **six** reacts with methyl iodide in methanol, after washing obtain **17**, white solid (0.071 g, 26%); ^1^H NMR (CDCl_3_) δ: 8.78 (d, *J* = 4.5 Hz, 1H), 8.11 (d, *J* = 9.3 Hz, 1H), 7.48 (dd, *J* = 9.3, 2.6 Hz, 1H), 7.37 (d, *J* = 4.7 Hz, 1H), 7.09 (d, *J* = 1.9 Hz, 1H), 7.02 (d, *J* = 2.6 Hz, 1H), 5.64 (ddd, *J* = 17.1, 10.5, 6.5 Hz, 1H), 5.21 (dd, *J* = 17.2, 1.5 Hz, 1H), 4.61 (td, *J* = 12.0, 5.5 Hz, 1H), 4.06 (s, 3H), 3.71 (s, 5H), 3.09 (s, 1H), 2.57 (t, *J* = 7.6 Hz, 2H), 2.40–2.19 (m, 4H), 1.82–1.50 (m, 10H), 1.42–1.21 (m, 5H), 0.96–0.87 (m, 3H); ^13^C NMR (101 MHz, CDCl_3_) δ: 171.56, 159.15, 147.10, 144.70, 137.96, 136.03, 132.83, 125.07, 122.21, 118.57, 118.44, 99.91, 67.11, 66.48, 65.80, 56.39, 54.85, 49.84, 37.39, 34.46, 31.16, 26.19, 25.08, 24.26, 22.23, 22.03, 13.83; HRMS *m/z*: calcd. For C_27_H_37_N_2_O_3_ [M]^+^ 437.2804 found 437.2801; m. p. 60.5 °C–61.3 °C.

#### 2.2.19 Synthesis of (1*S*,2*S*,4*S*,5*R*)-2-(*R*)-(6-methoxyquinolin-4-yl)(octanoyloxy)methyl)-1-methyl-5-vinylquinuclidin-1-ium iodide (18)

Following the general procedure, acylquinine **7** reacts with methyl iodide in methanol, after washing obtain **18**, yellow solid (0.044 g, 16%); ^1^H NMR (CDCl_3_) δ: 8.78 (d, *J* = 4.5 Hz, 1H), 8.11 (d, *J* = 9.3 Hz, 1H), 7.48 (dd, *J* = 9.3, 2.5 Hz, 1H), 7.37 (d, *J* = 4.6 Hz, 1H), 7.09 (d, *J* = 2.1 Hz, 1H), 7.03 (d, *J* = 2.7 Hz, 1H), 5.65 (ddd, *J* = 17.0, 10.5, 6.4 Hz, 1H), 5.26–5.06 (m, 2H), 4.58 (td, *J* = 11.8, 5.4 Hz, 1H), 4.06 (s, 3H), 3.82–3.72 (m, 2H), 3.71 (s, 3H), 3.69–3.54 (m, 2H), 3.08 (s, 1H), 2.57 (t, *J* = 7.5 Hz, 2H), 2.40–2.08 (m, 4H), 1.85–1.67 (m, 3H), 1.38–1.18 (m, 9H), 0.88 (t, *J* = 6.9 Hz, 3H); ^13^C NMR (CDCl_3_) δ: 171.57, 159.15, 147.08, 144.70, 137.99, 136.07, 132.78, 125.10, 122.28, 118.60, 118.41, 99.90, 67.04, 66.53, 65.73, 56.45, 54.88, 49.84, 37.39, 34.51, 31.56, 29.03, 28.84, 26.21, 25.08, 24.60, 22.55, 22.04, 14.04; HRMS *m/z*: calcd. For C_29_H_41_N_2_O_3_ [M]^+^ 465.3117 found 465.3135; m. p. 66.2 °C–67.2 °C.

#### 2.2.20 Synthesis of (1*S*,2*S*,4*S*,5*R*)-2-(*R*)-(6-methoxyquinolin-4-yl)(nonanoyloxy)methyl)-1-methyl-5-vinylquinuclidin-1-ium iodide (19)

Following the general procedure, acylquinine **8** reacts with methyl iodide in methanol, after washing obtain **19**, light yellow solid (0.050 g, 19%); ^1^H NMR (CDCl_3_) δ: 8.78 (d, *J* = 4.5 Hz, 1H), 8.11 (d, *J* = 9.3 Hz, 1H), 7.48 (dd, *J* = 9.3, 2.6 Hz, 1H), 7.37 (d, *J* = 4.6 Hz, 1H), 7.09 (d, *J* = 1.9 Hz, 1H), 7.02 (d, *J* = 2.7 Hz, 1H), 5.64 (ddd, *J* = 17.1, 10.5, 6.4 Hz, 1H), 5.26–4.84 (m, 1H), 4.61 (td, *J* = 11.9, 5.5 Hz, 1H), 4.06 (s, 3H), 3.71 (s, 3H), 3.60 (s, 7H), 3.15–3.03 (m, 1H), 2.57 (t, *J* = 7.6 Hz, 2H), 2.39–2.08 (m, 5H), 1.82–1.64 (m, 3H), 1.42–1.20 (m, 10H), 0.88 (t, *J* = 7.1 Hz, 3H); ^13^C NMR (CDCl_3_) δ: 171.56, 159.15, 147.09, 144.70, 137.96, 136.03, 132.82, 125.06, 122.22, 118.58, 118.43, 99.90, 67.11, 66.48, 65.77, 56.42, 54.84, 49.83, 37.39, 34.51, 31.74, 29.14, 29.08, 29.04, 26.20, 25.08, 24.61, 22.60, 22.04, 14.07; HRMS *m/z*: calcd. For C_30_H_43_N_2_O_3_ [M]^+^ 479.3274 found 479.3280; m. p. 66.7 °C–67.8 °C.

#### 2.2.21 Synthesis of (1*S*,2*S*,4*S*,5*R*)-2-(*R*)-(decanoyloxy)(6-methoxyquinolin-4-yl)methyl)-1-methyl-5-vinylquinuclidin-1-ium iodide (20)

Following the general procedure, acylquinine **9** reacts with methyl iodide in methanol, after washing obtain **20**, dark yellow solid (0.038 g, 15%); ^1^H NMR (CDCl_3_) δ: 8.79 (d, *J* = 4.5 Hz, 1H), 8.13 (d, *J* = 9.3 Hz, 1H), 7.49 (dd, *J* = 9.3, 2.6 Hz, 1H), 7.36 (d, *J* = 4.5 Hz, 1H), 7.07–7.12 (m, 1H), 7.00 (d, *J* = 2.6 Hz, 1H), 5.62 (ddd, *J* = 17.0, 10.5, 6.4 Hz, 1H), 5.06–5.24 (m, 2H), 4.92–5.02 (m, 1H), 4.04 (s, 3H), 3.70 (s, 3H), 3.45–3.68 (m, 4H), 3.09 (s ancho, 1H), 2.56 (t, *J* = 6.9 Hz, 2H), 2.03–2.39 (m, 3H), 1.65–1.81 (m, 4H), 1.22–1.36 (m, 12H), 0.88 (t, *J* = 7.6 Hz, 3H); ^13^C NMR (CDCl_3_) δ: 171.55, 159.16, 147.14, 144.71, 137.93, 135.99, 132.91, 122.11, 118.53, 118.47, 99.91, 67.22, 66.39, 65.91, 56.28, 54.75, 49.78, 37.38, 34.49, 31.82, 29.35, 29.22, 29.19, 29.09, 26.16, 25.07, 24.62, 22.65, 22.02, 14.10; HRMS *m/z*: calcd. For C_31_H_45_N_2_O_3_ [M]^+^ 493.3430 found 493.3426; m. p. 61.1 °C–61.8 °C.

#### 2.2.22 Synthesis of (1*S*,2*S*,4*S*,5*R*)-2-((*R*)-(6-methoxyquinolin-4-yl)(palmitoyloxy)methyl)-1-methyl-5-vinylquinuclidin-1-ium iodide (21)

Following the general procedure, acylquinine **10** reacts with methyl iodide in methanol, After washing obtain **21**, yellow solid (0.037 g, 15%); ^1^H NMR (CDCl_3_) δ: 8.78 (d, *J* = 4.5 Hz, 1H), 8.10 (d, *J* = 9.3 Hz, 1H), 7.47 (dd, *J* = 9.3, 2.5 Hz, 1H), 7.37 (d, *J* = 4.6 Hz, 1H), 7.08 (d, *J* = 2.0 Hz, 1H), 7.04 (d, *J* = 2.6 Hz, 1H), 5.65 (ddd, *J* = 17.1, 10.5, 6.4 Hz, 1H), 5.05–5.27 (m, 2H), 4.86 (dd, *J* = 12.9, 10.5 Hz, 1H), 4.55 (td, *J* = 11.7, 5.2 Hz, 1H), 4.07 (s, 3H), 3.71–3.86 (m, 3H), 3.71 (s, 3H), 3.56–3.68 (m, 1H), 3.08 (s, 1H), 2.57 (t, *J* = 7.6 Hz, 2H), 2.06–2.40 (m, 4H), 1.63–1.80 (m, 4H), 1.19–1.38 (m, 26H), 0.88 (t, *J* = 6.8 Hz, 3H); ^13^C NMR (CDCl_3_) δ: 171.57, 159.14, 147.05, 144.68, 138.04, 136.09, 132.72, 125.12, 122.33, 118.61, 118.38, 99.91, 66.95, 66.57, 65.71, 56.47, 54.92, 49.86, 37.42, 34.53, 31.92, 29.69, 29.68, 29.66, 29.65, 29.63, 29.58, 29.41, 29.36, 29.21, 29.10, 26.24, 25.10, 24.62, 22.69, 22.05, 14.12; HRMS *m/z*: calcd. For C_37_H_57_N_2_O_3_ [M]^+^ 577.4369 found 577.4373; m. p. 56.9 °C–57.6 °C.

#### 2.2.23 Synthesis of (1*S*,2*S*,4*S*,5*R*)-2-((*R*)-(6-methoxyquinolin-4-yl)(((*E*)-octadec-9-enoyl) oxy) methyl)-1-methyl-5-vinylquinuclidin-1-ium iodide (22)

Following the general procedure, acylquinine **11** reacts with methyl iodide in methanol, after washing obtain **22**, dark yellow solid (0.050 g, 20%), ^1^H NMR (CDCl_3_) δ: 8.78 (d, *J* = 4.5 Hz, 1H), 8.10 (d, *J* = 9.3 Hz, 1H), 7.47 (dd, *J* = 9.3, 2.5 Hz, 1H), 7.37 (d, *J* = 4.6 Hz, 1H), 7.08 (d, *J* = 2.1 Hz, 1H), 7.04 (d, *J* = 2.6 Hz, 1H), 5.66 (ddd, *J* = 17.0, 10.5, 6.4 Hz, 1H), 5.29–5.39 (m, 2H), 5.06–5.28 (m, 2H), 4.86 (dd, *J* = 12.9, 10.4 Hz, 1H), 4.55 (td, *J* = 11.6, 5.1 Hz, 1H), 4.07 (s, 3H), 3.72–3.90 (m, 2H), 3.71 (s, 3H), 3.56–3.69 (m, 1H), 3.08 (s, 1H), 2.57 (t, *J* = 7.6 Hz, 2H), 1.97–2.27 (m, 7H), 1.22–1.38 (m, 20H), 0.87 (t, *J* = 6.6 Hz, 3H); ^13^C NMR (CDCl_3_) δ: 171.54, 159.14, 147.04, 144.69, 138.02, 136.09, 132.72, 130.13, 129.59, 125.12, 122.34, 118.62, 118.39, 99.90, 66.96, 66.58, 65.66, 56.51, 54.92, 49.88, 37.41, 34.52, 31.90, 29.76, 29.66, 29.52, 29.32, 29.30, 29.13, 29.06, 29.04, 27.24, 27.13, 26.25, 25.09, 24.60, 22.68, 22.05, 14.12; HRMS *m/z*: calcd. For C_39_H_59_N_2_O_3_ [M]^+^ 603.4526 found 603.4543 m. p. 62.0 °C–62.9 °C.

#### 2.2.24 Synthesis of (1*S*,2*S*,4*S*,5*R*)-2-((*R*)-((cyclohexanecarbonyl)oxy)(6-methoxyquinolin-4-yl)methyl)-1-methyl-5-vinylquinuclidin-1-ium iodide (23)

Following the general procedure, acylquinine **12** reacts with methyl iodide in methanol, After washing obtain **23**, light yellow solid (0.069 g, 26%);^1^H NMR (CDCl_3_) δ: 8.78 (d, *J* = 4.6 Hz, 1H), 8.10 (d, *J* = 9.3 Hz, 1H), 7.48 (dd, *J* = 9.3, 2.5 Hz, 1H), 7.37 (d, *J* = 4.5 Hz, 1H), 7.09 (s, 1H), 7.03 (d, *J* = 2.6 Hz, 1H), 5.65 (ddd, *J* = 17.0, 10.5, 6.5 Hz, 1H), 5.16 (dd, *J* = 16.2, 1.6 Hz, 2H), 4.92 (q, *J* = 10.6 Hz, 1H), 4.07 (s, 3H), 3.85–3.73 (m, 1H), 3.70 (s, 3H), 3.64–3.48 (m, 1H), 3.08 (sw, 1H), 2.54 (tt, *J* = 11.3, 3.7 Hz, 1H), 2.40–1.97 (m, 6H), 1.88–1.70 (m, 4H), 1.58–1.20 (m, 6H); ^13^C NMR (101 MHz, CDCl_3_) δ:173.75, 159.14, 147.06, 144.68, 138.16, 136.17, 132.72, 125.13, 122.39, 118.50, 118.37, 99.81, 66.96, 66.32, 65.61, 56.48, 54.78, 49.81, 43.28, 37.37, 29.06, 28.69, 26.24, 25.40, 25.27, 25.15, 25.10, 22.14.; HRMS *m/z*: calcd. For C_28_H_37_N_2_O_3_ [M]^+^ 449.2799 found 449.2791; m. p. 102.2 °C–103.6 °C.

#### 2.2.25 Synthesis of (1*S*,2*S*,4*S*,5*R*)-2-((*R*)-(benzoyloxy)(6-methoxyquinolin-4-yl)methyl)-1-methyl-5-vinylquinuclidin-1-ium iodide (24)

Following the general procedure, acylquinine **13** reacts with methyl iodide in methanol, after washing obtain **24**, white solid (0.067 g, 24%); ^1^H NMR (CDCl_3_) δ: 8.77 (d, *J* = 4.6 Hz, 1H), 8.11–8.17 (m, 3H), 7.75 (t, *J* = 7.5 Hz, 1H), 7.60 (t, *J* = 7.9 Hz, 2H), 7.49–7.52 (m, 2H), 7.40 (s, 1H), 7.10 (d, *J* = 2.6 Hz, 1H), 5.67 (ddd, *J* = 17.1, 10.5, 6.5 Hz, 1H), 5.09–5.26 (m, 2H), 4.92–5.03 (m, 1H), 4.61 (td, *J* = 12.3, 5.6 Hz, 1H), 4.07 (s, 3H), 3.97 (s, 1H), 3.80–3.90 (m, 1H), 3.75 (s, 3H), 3.62 (t, *J* = 12.4 Hz, 1H), 3.11 (s, 1H), 2.27–2.48 (m, 3H), 2.10–2.24 (m, 1H), 1.82–1.95 (m, 2H); ^13^C NMR (CDCl_3_) δ: 164.31, 159.16, 147.21, 144.64, 137.70, 136.04, 135.10, 132.86, 129.78, 129.66, 129.43, 128.68, 127.72, 125.04, 122.19, 118.71, 118.50, 99.94, 67.30, 66.93, 65.88, 56.38, 54.72, 49.81, 37.36, 26.23, 25.23, 22.37; HRMS *m/z*: calcd. For C_28_H_31_N_2_O_3_ [M]^+^ 443.2335 found 443.2343; m. p. 104.2 °C–105.4 °C.

#### 2.2.26 Synthesis of (1*S*,2*S*,4*S*,5*R*)-2-((*R*)-(cinnamoyloxy)(6-methoxyquinolin-4-yl)methyl)-1-methyl-5-vinylquinuclidin-1-ium iodide (25)

Following the general procedure, acylquinine **14** reacts with methyl iodide in methanol, After washing obtain **25**, white solid (0.024 g, 19%); ^1^H NMR (CDCl_3_) δ: 8.78 (d, *J* = 4.6 Hz, 1H), 8.11 (d, *J* = 9.3 Hz, 1H), 7.86 (d, *J* = 15.9 Hz, 1H), 7.67–7.59 (m, 2H), 7.47 (dd, *J* = 8.5, 6.1 Hz, 5H), 7.23 (s, 1H), 7.07 (d, *J* = 2.6 Hz, 1H), 6.65 (d, *J* = 15.9 Hz, 1H), 5.67 (s, 1H), 5.27–5.06 (m, 2H), 4.87 (t, *J* = 11.7 Hz, 1H), 4.60–4.49 (m, 1H), 4.07 (s, 3H), 3.89–3.78 (m, 2H), 3.73 (s, 3H), 3.08 (s, 1H), 2.37–2.20 (m, 4H), 1.74–1.63 (m, 2H), 1.25 (s, 1H); ^13^C NMR (101 MHz, CDCl_3_) δ: 164.76, 159.12, 149.51, 147.23, 144.68, 137.85, 136.11, 133.25, 132.78, 131.75, 129.22, 129.00, 128.78, 128.28, 125.13, 122.22, 118.91, 118.40, 114.84, 100.02, 67.18, 66.67, 65.80, 56.48, 54.89, 49.82, 37.46, 29.70, 26.27, 25.18, 22.14.; HRMS *m/z*: calcd. For C_30_H_33_N_2_O_3_ [M]^+^ 469.2486 found 469.2501; m. p. 102.2 °C–103.6 °C.

### 2.3 Biological assays

#### 2.3.1 Cell line and culture conditions

Three cell lines were used: two leukemia cell lines and one non-carcinogenic cell line. Acute lymphoblastic leukemia CCRF-CEM (ATCC CCL-119) and acute T-cell leukemia Jurkat Clone E6-1 (ATCC TIB-152) were grown in RPMI-1640 culture medium supplemented with 10% inactivated fetal bovine serum (FBS; Seraprime, LLC, CO, USA), 100 U/mL penicillin, and 100 μg/mL streptomycin. The human non-carcinogenic fibroblast cell line Hs27 (ATCC, CRL-1634) was grown in DMEM (Corning, NY, USA) supplemented with 10% inactivated FBS, 100 U/mL penicillin, and 100 μg/mL streptomycin. All cell lines were incubated at 37 °C and 5% CO_2_.

#### 2.3.2 Differential nuclear staining (DNS) assay

To evaluate the compounds’ cytotoxicity, the 50% cytotoxic concentration (CC_50_) was determined using the Differential nuclear staining (DNS) assay, which was performed on the cell lines CEM, Jurkat, and Hs27 (Lema et al., 2011). Cells were seeded into 96-well plates at 10,000 cells per well in 100 µL of complete medium. After overnight incubation at 37 °C in 5% CO_2_, compounds at concentrations ranging from 40 μM to 1.25 µM were tested in triplicate. Control treatments were also included: 1% v/v DMSO as a vehicle; untreated (UNT) cells as a negative control for cell death; and 1 mM hydrogen peroxide (H_2_O_2_) as a positive control, all in triplicate. After a 48h–72 h incubation under optimal cell culture conditions, a 1 μg/mL concentration of DNA-intercalating fluorescent dyes Hoechst 33,342 and propidium iodide (PI) was added, and the cells were analyzed with the ImageXpress Pico bioimager (Molecular Devices, San Jose, CA, USA) after 1 h. Hoechst 33,342 (blue) stains the nuclei of dead and live populations, while PI (red) stains the nuclei of dead cells by passing through their permeable membranes (Aishwarya et al., 2025). Four continuous images were acquired from each well, forming a 2 × 2 montage across two fluorescence channels. Death percentages were normalized to the total cell count of the DMSO vehicle controls.

#### 2.3.3 Apoptosis assay

To assess the mechanism of cell death induced by compound **22** in CEM cells, apoptosis was measured using the Annexin V-FITC/PI kit (Beckman Coulter, Miami, FL, USA) according to the manufacturer’s instructions. Phosphatidylserine (PS) externalization is a hallmark of apoptosis. PS is a phospholipid that resides in the inner leaflet of healthy cells and, upon damage, externalizes to the outer leaflet, signaling nearby phagocytes to engulf the cells *in vivo* (Fadok et al., 1998). Annexin has a high affinity for PS and is commonly used as a probe to detect PS on the outer leaflet of the cell membrane. When conjugated with FITC, this interaction results in green fluorescence. CEM cells were seeded at 100,000 cells/mL in 24-well plates (1 mL per well) and incubated at 37 °C in 5% CO_2_ for at least 2 h. Treatments were added in triplicate as follows: compound **22**, 24 h CC_50_ (2.25 µM); 22, 24 h 2x CC_50_ (4.5 µM); 1% v/v DMSO; 1 mM H_2_O_2_; and UNT cells. After 24 h of incubation at 37 °C and 5% CO_2_, cells were collected into flow cytometry tubes and centrifuged at 1,200 rpm for 5 min. Supernatants were discarded, and a 100 µL mixture of Annexin V-FITC, PI, and 1X binding buffer was added to each sample, which was then gently resuspended. Tubes were incubated in an ice bath in the dark for 15 min and then analyzed on the Gallios™ flow cytometer. The FL1 and FL2 channels were used to detect green and red fluorescence signals, respectively. Approximately 10,000 events were recorded per sample, and data were analyzed using Kaluza 1.3 software (Beckman Coulter, Miami, USA). Cells positive for Annexin V-FITC and PI were classified as late apoptotic, cells only positive for Annexin V-FITC were classified as early apoptotic, and cells only positive for PI were classified as necrotic.

#### 2.3.4 Mitochondrial depolarization assay

Mitochondrial depolarization was assessed using the MitoProbe JC-1 Assay Kit for Flow Cytometry (Molecular Probes, M34152). JC-1 is a cationic, polychromatic dye that accumulates in the mitochondria of healthy cells and emits a red fluorescence. In cells with damaged mitochondrial membranes, the aggregates of JC-1 do not form and instead emit green fluorescence. Cells were seeded in 24-well plates at a density of 100,000 cells/mL and treated in triplicate with compound **22**, 24 h CC_50_ (2.25 µM), H_2_O_2_ (2 mM), DMSO (1% *v/v*), or left untreated (UNT). The cells were incubated for 5 h at 37 °C in 5% CO_2_. After the incubation period, cells were transferred to flow cytometry tubes and centrifuged at 1,200 rpm for 5 min. Supernatants were discarded, and cell pellets were resuspended in 500 μL of pre-warmed 1X PBS containing JC-1 dye at a final concentration of 2 μM. The cells were incubated for 30 min, washed with 500 μL of pre-warmed 1X PBS, centrifuged again (1,200 rpm, 5 min), and resuspended in 500 μL of 1X PBS. Samples were immediately analyzed in the Gallios™ flow cytometer. The FL1 and FL2 channels were used to detect green and red fluorescence, respectively. Approximately 10,000 events were recorded per sample, and data were analyzed using Kaluza 1.3 software (Beckman Coulter, Miami, USA).

#### 2.3.5 Caspase-3 assay

Caspase-3/7 activation was assessed in CEM cells using the NucView 488 caspase-3 fluorogenic substrate (Biotium, #10402, Fremont, USA). CEM cells were seeded at 100,000 cells/mL in 24-well plates and treated with **22** CC_50_ (2.25 µM) and **22** 2x CC_50_ (4.5 µM). The cells were incubated at 37 °C with 5% CO_2_ for 5 h, then collected into flow cytometry tubes and centrifuged at 1,200 rpm for 5 min. The cells were then washed with 100 µL of PBS containing the caspase-3 substrate at a final concentration of 2 µM and incubated in the dark at room temperature for 30 min. After incubation, 300 µL of pre-warmed PBS was added, and the samples were then transferred to the flow cytometer for analysis. The NucView 488 substrate produces a green, fluorescent signal in cells with activated caspase-3/7 (Cen et al., 2008). The FL1 detector was used to detect cells with activated caspase-3. Approximately 10,000 events were obtained per sample, and the data were analyzed as described in the previous sections.

#### 2.3.6 Cell cycle analysis

The effects of compound **22** on the cell cycle profile were analyzed using nuclear isolation medium (NIM) with DAPI, which quantifies DNA content across stages by staining the cells’ nuclei (Gutierrez et al., 2022). A density of 100,000 cells/mL (CEM) was seeded in 24-well plates and treated in triplicate with **22** CC_20_ (0.9 µM), **22** CC_30_ (1.35 µM), and **22** CC_50_ (2.25 µM), 1% DMSO, 1 mM H_2_O_2_, positive control Etoposide (ETP) topoisomerase inhibitor, and no treatment (UNT). Lower concentrations of **22** were used to avoid excess DNA fragmentation, which could interfere with the other phases of the cell cycle. The plate was incubated for 72 h at 37 °C in 5% CO_2_. After 72 h, cells were collected in flow cytometry tubes and centrifuged at 1,200 rpm for 5 min. Supernatant was discharged from each tube, and a mixture of 200 µL Phosphate-Buffered Saline (PBS) and 200 µL NIM-DAPI was added to the pellet. Samples were analyzed using a Gallios™ flow cytometer with Kaluza 1.3 software (Beckman Coulter, Miami, USA). For each sample, 10,000 events were acquired using the FL9 detector to capture the NIM-DAPI fluorescent signal at ∼461 nm (Gutierrez et al., 2022).

### 2.4 Computational studies

#### 2.4.1 Molecular docking

The binding mechanisms of the compounds **7**, **8**, and **22**, synthesized molecules to the active sites of human protein kinases, PIM-1 (PDB code: 4DTK) (Cozza et al., 2014) and CK2α (PDB code: 4 kW P) (Cozza et al., 2014), were investigated using AutoDock V 4.2 (Morris et al., 2009). Inhibition of CK2 and/or PIM-1 kinases has been shown to induce apoptosis in a variety of cancer cell lines, underscoring their potential as valuable targets in anti-cancer drug development. The experimental and theoretical studies have been validated using the 4,5,6,7-tetrabromo-1H-benzimidazole (TBBI) as a positive control for those inhibition mechanisms (Wińska et al., 2025). Water molecules, cofactors, and ions were removed from the X-ray crystallographic structure during protein preparation. Polar hydrogen atoms were added, and atomic charges were computed using the Gasteiger method, while the non-polar hydrogen atoms were merged. The grid maps required by AutoDock were generated using the auxiliary program AutoGrid, selecting a 60 × 60 × 60 Å grid box centered on the active site. The Lamarckian Genetic Algorithm (LGA) was used to conduct docking searches (Morris et al., 1998) with a population of 2,000 individuals, resulting in 2,500,000 energy evaluations per 50-run LGA. The best docking complex poses were analyzed based on intermolecular interactions (ligand/enzyme), including hydrogen bonding, hydrophobic interactions, and cation–π, and π–π stacking, using the protein–ligand interaction profiler PLIP web platform (Adasme et al., 2021).

#### 2.4.2 Molecular dynamics simulations (MD)

MD experiments were conducted on the ligand-PIM-1(PDB:4DTK) and ligand-CK2α (PDB:4 kW P) complexes in aqueous solutions containing **7**, **8**, **22**, and TBBI as ligands, using the TIP3P water model as an explicit solvent (Neria et al., 1996) (≈16.000 water molecules). Additionally, Na^+^ and Cl^−^ ions were added to neutralize the system and maintain an ionic concentration of 0.15 mol⋅ L^-1^. A general AMBER force field (GAFF) was used to parameterize all molecules (Wang et al., 2004), and the protein structure was modeled using the CHARMM27 force field (Brooks et al., 2009). The simulations followed a standard MD protocol: (i) minimization and structural relaxation of water molecules were performed using 2,000 stages of minimization (downward step) and MD simulations with an NPT (310 K) assembly by 1,000 ps, using harmonic constraints of 10 kcal⋅mol Å^−2^ on the protein and ligand; (ii) a complete structure minimization considering 2,000 downstream minimization steps and 6,500 steps of conjugate gradient minimization; (iii) the minimized systems were progressively heated to 310 K over 0.5 ns, with harmonic restrictions of 10 kcal⋅mol Å^−2^ in the carbon skeleton and ligand; (iv) the system was then balanced for 0.5 ns while adhering to the constraints, and then for 5 ns without constraints to 310 K in a canonical assembly (NVT); and (v) a production dynamic was conducted for 100 ns without constraints at 310 K and 1 atm with a temporary passage of 2 fs using an isothermal isobaric assembly (NPT). In the MD simulation, Langevin dynamics controlled the temperature with a collision frequency of 1 ps ^−1^ (NVT), and the pressure was controlled by a Berendsen barostat (NPT). In addition, the Particle Mesh Ewald (PME) method with a 10 Å cutoff was used to handle nonbonded and long-range electrostatic interactions. All MD simulations were performed using NAMD, developed by the Theoretical and Computational Biophysics Group at the Beckman Institute for Advanced Science and Technology at the University of Illinois at Urbana-Champaign (Phillips et al., 2005; 2020). Molecular visualization of the systems and MD trajectory analysis were performed using the VMD software package (Humphrey et al., 1996).

#### 2.4.3 Free energy calculations

The binding free energies for PIM-1-ligand and CK2α-ligand complexes were estimated using the molecular MM/GBSA technique. For computational purposes, the last 40 nanoseconds of each molecular dynamics simulation were extracted, and explicit water molecules and ions were subsequently removed. Three subsets of each system were analyzed using MM/GBSA: the protein alone, the ligand alone, and the complex (protein–ligand). The total free energy (ΔGtot) for each subset is calculated as follows:

$$\Delta G=E_{MM}+Gsolv-{T\Delta S}_{conf}$$

Where E_MM_ represents the bonded and Lennard–Jones energy components, G_Solv_ denotes the polar and nonpolar contributions to the solvation energy, T represents the temperature, and Δ_Sconf_ denotes the conformational entropy (Hayes and Archontis, 2012). Both E_MM_ and G_Solv_ were calculated using NAMD with the generalized Born implicit-solvent model (Götz et al., 2012). ΔG_tot_ was calculated as a linear function of the solvent-accessible surface area with a probe radius of 1.4 Å (Abroshan et al., 2010). The difference between the binding free energies of the complex, protein, and ligand was used to compute the binding free energy of each complex (ΔG_bind_). In this representation, the values signify the averages obtained from the simulation.

$$\Delta G_{bind}=G_{tot}\left(complex\right)-G_{tot}\left(protein\right)-G_{tot}\left(ligand\right)$$

## 3 Results and discussion

### 3.1 Chemistry

The hemisynthesis of quinine derivatives (Figure 1) involved a nucleophilic acylation reaction (Echeverría et al., 2017). In these reactions, acyl chlorides of varying chain lengths were employed to enhance the compounds’ lipophilicity in biological assays. This is series 1 of acylquinine derivatives 2–14. In series 2 of acylquinine salt derivatives 15–25, they were obtained by methylation with methyl iodide (Joyce et al., 2016) to form the quaternary ammonium, thereby yielding the quaternary amphiphilic compounds. The synthetic methodology is described in Figure 1, and the yields obtained are listed in Table 1. They range from 14% to 82%, with compound **7** yielding the highest percentage. ^1^H/^13^C NMR characterized the compounds, and HRMS confirmed their authenticity. Characteristic signals of the quinidine scaffold are observed in all spectra as protons belonging to the methoxy group at approximately 3.96–3.98 ppm, and in the region between 5.00 and 5.30 ppm, the protons of the double bond. The aromatic region is distinguished by the presence of five signals indicating the presence of the quinoline ring, except for compounds **13**, **24** (benzoyl) and **14**, **25** (cinnamoyl), which have an aromatic system in the acylated chain. Additionally, a signal integrating three protons corresponds to the methyl group added for the formation of the ammonium salt, which is located around 3.70 ppm in all synthesized compounds; this is a key signal that confirms the production of the desired compounds.

**TABLE 1:** Yield (%) of each compound in series 1 and series 2.

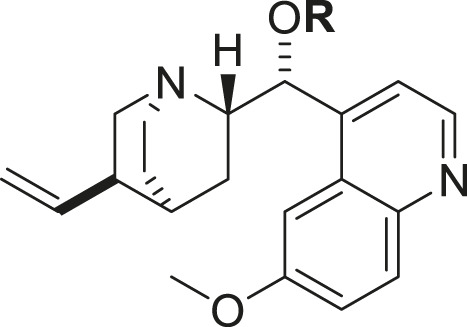			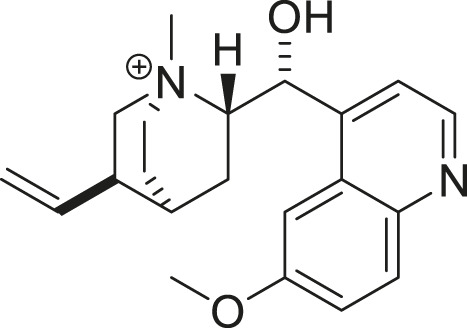			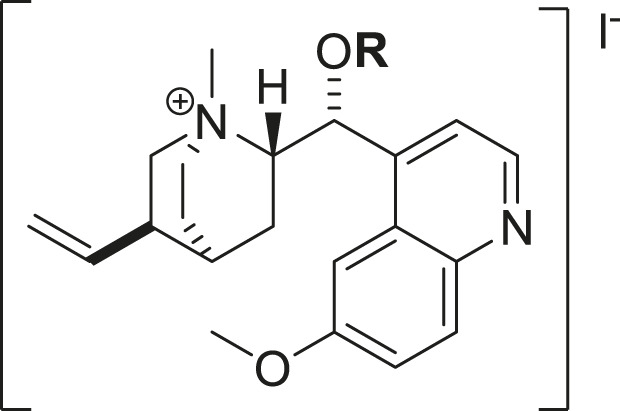		

Series 1			Series 2					
Comp.	R	Yield (%)	Comp.	R	Yield (%)	Comp.	R	Yield (%)
2	Acetyl	56	15	—	33	16	Butyryl	27
3	Propionyl	73	​	​	​	17	Hexanoyl	26
4	Butyryl	69	​	​	​	18	Octanoyl	16
5	Pentanoyl	72	​	​	​	19	Nonanoyl	19
6	Hexanoyl	72	​	​	​	20	Decanoyl	15
7	Octanoyl	82	​	​	​	21	Palmitoyl	15
8	Nonanoyl	77	​	​	​	22	Oleoyl	20
9	Decanoyl	39	​	​	​	23	Cyclohexanoyl	26
10	Palmitoyl	14	​	​	​	24	Benzoyl	24
11	Oleoyl	57	​	​	​	25	Cinnamoyl	19
12	Cyclohexanoyl	46	​	​	​	​	​	​
13	Benzoyl	61	​	​	​	​	​	​
14	Cinnamoyl	31	​	​	​	​	​	​

### 3.2 Differential nuclear staining (DNS) assay identified compound 22 to be the most cytotoxic of the series

The DNS assay was used to evaluate the cytotoxicity of 26 compounds from the acylquinine series 1 and acylquinine salts series 2 in a panel of cell lines, including CEM (acute lymphoblastic leukemia), Jurkat (acute T-cell leukemia), and Hs27 (normal human fibroblasts) (Lema et al., 2011). A preliminary screening was conducted to assess the cytotoxic effects of all synthesized compounds following 48 and 72 h of exposure.

Among the tested compounds, compound **22** demonstrated potent cytotoxic activity across all three cancer cell lines, with CC_50_ values of 2.25 ± 0.03 μM and 4.1 ± 0.05 μM for the CEM and Jurkat lines, respectively (Table 2). Notably, its cytotoxicity was most pronounced in the CEM cell line.

**TABLE 2:** CC_50_ values of the synthesized compounds in Series **1** and **2** were determined by the DNS assay in CEM and Jurkat cells after 48 h of exposure.

Compound	CC_50_ (µM)	
	CEM	JURKAT
2	>40	>40
3	43.3 ± 0.21	>40
4	44.0 ± 0.16	>40
5	24.9 ± 0.60	36.0 ± 0.30
6	8.5 ± 0.24	11.6 ± 0.30
7	6.4 ± 0.14	8.4 ± 0.15
8	7.3 ± 0.21	8.1 ± 0.15
9	9.0 ± 0.31	15.4 ± 0.23
10	>40	33.9 ± 2.90
11	>40	>40
12	52.7 ± 5.90	>40
13	26.6 ± 0.36	33.5 ± 0.23
14	8.7 ± 0.30	35.2 ± 0.40
15	>40	>40
16	>40	37.8 ± 1.90
17	>40	12.7 ± 0.70
18	>40	>40
19	>40	34.7 ± 0.60
20	>40	36.4 ± 0.30
21	8.0 ± 1.57	12.3 ± 0.90
22	2.3 ± 0.03	4.1 ± 0.05
23	35.8 ± 0.90	>40
24	>40	>40
25	56.2 ± 1.90	>40

In addition to compound **22**, other compounds exhibiting CC_50_ values below 10 μM in CEM cells included compounds **6**, **7**, **8**, **9**, **14**, and **21**, with values of 8.5 ± 0.24 μM, 6.4 ± 0.14 μM, 7.25 ± 0.21 μM, 9 ± 0.31 μM, 8.7 ± 0.3 μM, and 8.01 ± 1.57 μM, respectively. In Jurkat cells, compounds **7** and **8** were the most active, with CC_50_ values of 6.4 ± 0.14 μM and 7.25 ± 0.21 μM, respectively.

Structure–activity relationship analysis in the CEM line indicated that medium-chain acyl derivatives (6–10 carbon atoms) from series **1** displayed greater cytotoxicity than their series **2** analogs. Conversely, long-chain acyl derivatives (oleoyl and palmitoyl) from series **1** were markedly less active than those in series **2**. Specifically, compound **22**, which exhibits the best cytotoxic activity against CEM and Jurkat cells, is the salt with the longest chain in the entire two series (18 carbon atoms). Comparatively, the improvement in activity over compound **11**, the neutral molecule with the same chain length, is notable, suggesting the importance of ammonium salt compounds as biologically relevant alternatives.

Only compounds with CEM CC_50_ values below 10 µM were evaluated in the non-cancerous Hs27 cell line. These included compounds **6**, **7**, **8**, **9**, **14**, **21**, and **22**.

As shown in Table 3, the compounds evaluated in Hs27 cells exhibited higher CC_50_ values than those observed in CEM cells. Compounds 7 and 9 displayed the highest CC_50_ values, 33.2 ± 0.41 µM and 37.1 ± 7.14 µM, respectively. These findings demonstrate that the compounds are more selective for the leukemia cell lines CEM and Jurkat than for the non-cancerous Hs27 cells.

**TABLE 3:** Evaluation of CC_50_ values of the compound in CEM cells was conducted in non-cancerous Hs-27 cells after 72 h of exposure.

Compound	CC_50_ (µM)		SCI*
	Hs-27	CEM	
6	28.1 ± 1.46	8.5 ± 0.24	3.3
7	33.2 ± 0.41	6.4 ± 0.14	5.2
8	26.6 ± 2.57	7.3 ± 0.21	5.1
9	37.1 ± 7.14	9.0 ± 0.31	4.1
14	27.3 ± 11.50	8.7 ± 0.30	3.1
21	27.2 ± 1.35	8.01 ± 1.57	3.4
22	10.0 ± 0.81	2.25 ± 0.03	4.5

*Selective Cytotoxicity Index (SCI) values were determined using the following equation: CC_50_ of non-cancer cells (Hs27) divided by the CC_50_ of the cancer CEM, cell line (Robles-Escajeda et al., 2016).

The Selective Cytotoxicity index (SCI), calculated as the ratio of the CC_50_ value in normal cells to that in tumor cells (Robles-Escajeda et al., 2016), was derived from the data presented in Table 3. An SCI value greater than 1 indicates preferential cytotoxicity toward cancer cells, whereas a value below 1 suggests greater toxicity toward normal cells. According to this analysis, compounds **7**, **8**, and **22** exhibited the highest SCI values of 5.2, 5.1, and 4.5, respectively, suggesting a favorable selectivity profile toward cancer cells. These findings support the hypothesis that these compounds may possess tumor-selective properties.

Based on these results, a more in-depth investigation into the mechanism of action of compound **22** was conducted in the CEM cell line.

### 3.3 Compound 22 induces apoptosis in CEM cells

To determine whether compound **22** induces apoptosis in CEM cells, the Annexin V-FITC/PI assay was performed by flow cytometry. This assay detects the externalization of phosphatidylserine (PS) and the binding of Annexin V to these PS residues and is widely used to detect cells in the early stages of apoptosis (Eskandari and Eaves, 2022). Using this assay, CEM cells were treated with compound **22** at its CC_50_ concentration (2.25 ± 0.03 μM) and incubated for 24 h. DMSO, H_2_O_2_, and UNT cells were included as controls.

**FIGURE 2:**
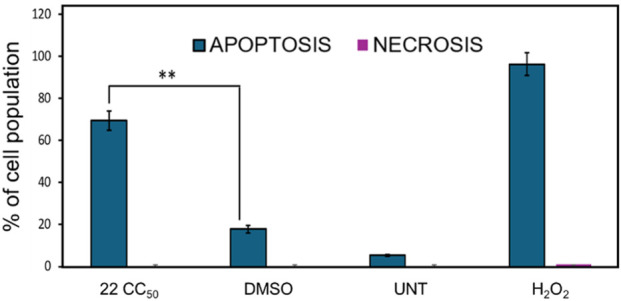
In the bar chart, the total percentages of apoptosis (early and late) are shown in blue bars, and the percentages of necrotic cells are shown in purple bars. CEM cells exposed to compound **22** showed significant PS externalization. Apoptotic cells with exposed PS are FITC-positive, indicating an early stage of apoptosis induction. Cells positive for both FITC and PI indicate late apoptosis, while cells emitting only the PI signal are classified as necrotic.

As shown in Figure 2, compound **22** induced a 69.5% increase in Annexin V-positive cells, indicating significant phosphatidylserine (PS) externalization and subsequent apoptosis induction in CEM cells. In comparison, the DMSO control showed a lower percentage (17.8%), whereas the H_2_O_2_ control displayed high levels of PS externalization.

### 3.4 Compound 22 induces a loss of mitochondrial membrane potential in CEM cells

The loss of mitochondrial membrane potential is a hallmark event in the intrinsic apoptosis pathway. Depolarization of the mitochondrial membrane allows cytochrome c, a protein in the inner mitochondrial membrane, to be released into the cytoplasm. This protein forms the apoptosome, which activates caspase-3, leading to proteolytic cleavage of cellular proteins and DNA damage, thereby inducing apoptosis (Kwak et al., 2023). In this assay, the ability of compound **22** to induce the intrinsic mitochondrial apoptotic pathway was evaluated. The MitoProbe JC-1 assay kit (Molecular Probes, M34152) and flow cytometry were used. A significant increase in mitochondrial depolarization was detected in CEM cells after treatment with compound **22** at two concentrations (CC_50_ and 2x CC_50_) compared with the DMSO control. Cells with damaged mitochondria were detected by a green fluorescent signal, indicating that the treated cells had lost mitochondrial membrane potential.

**FIGURE 3:**
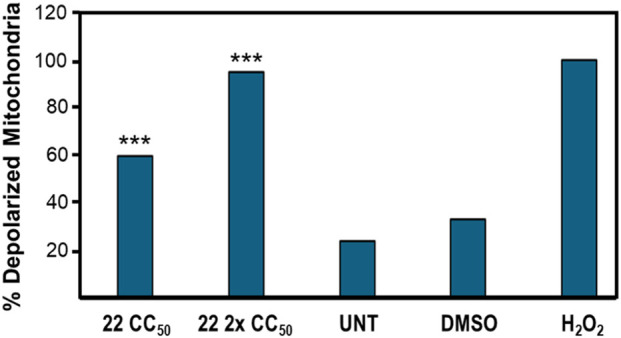
Compound **22** induced mitochondrial depolarization in CEM cells in a dose-dependent manner. The Mito-Probe JC-1 and flow cytometry were used to collect data.

As shown in Figure 3, the results demonstrate that compound **22** induced mitochondrial depolarization in 59.7% at the CC_50_ concentration and 95% at the 2x CC_50_ concentration, compared to 33.2% in the DMSO vehicle control. These data indicate that compound **22** activates the intrinsic apoptosis pathway *via* loss of mitochondrial membrane potential.

### 3.5 Compound 22 triggers caspase-3/7 activation

Caspase-3 is considered a vital biomarker because its activation leads to cell shrinkage, chromatin condensation, cellular blebbing, and externalization of phosphatidylserine (PS), ultimately culminating in cell death and phagocytosis of apoptotic bodies (Lauwerys et al., 2024).

**FIGURE 4:**
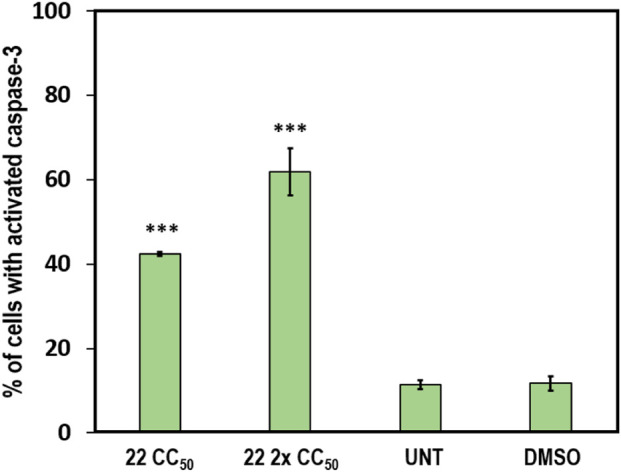
A significant activation of caspase-3/7 was observed after treating CEM cells for 5 h with compound **22**.

To determine the onset of apoptosis in CEM cells treated with compound **22**, caspase-3 activation was assessed by flow cytometry at 5 h post-treatment. In this assay, apoptotic cells with activated caspase-3/7 cleave the NucView-488 caspase-3 substrate, releasing a high-affinity DNA dye that generates a bright green, fluorescent signal upon migration to the nucleus. As expected, cells treated with compound **22** showed a significant increase in caspase-3/7 activation, with 42.4% at the CC_50_ concentration and 61.9% at the 2x CC_50_ concentration, compared to 11.6% in the DMSO solvent control (Figure 4). These results indicate that treatment with compound **22** triggers caspase-3/7 activation and induces apoptosis.

### 3.6 Compound 22 interferes with cell cycle progression

The effects on the cell cycle progression were assessed in CEM cells treated with 2.25 µM (CC_50_), 1.35 µM (CC_30_), and 0.9 µM (CC_20_) of compound **22**. Control treatments included DMSO (1%), etoposide (ET) (10 µM), and untreated (UNT). Cells were incubated for 72 h with the treatments and subsequently stained with a NIM-DAPI solution to quantify DNA content by flow cytometry. Lower concentrations of the compound were used in this assay to prevent excessive DNA fragmentation, which could have impaired visualization of any potential arrest at different stages.

**FIGURE 5:**
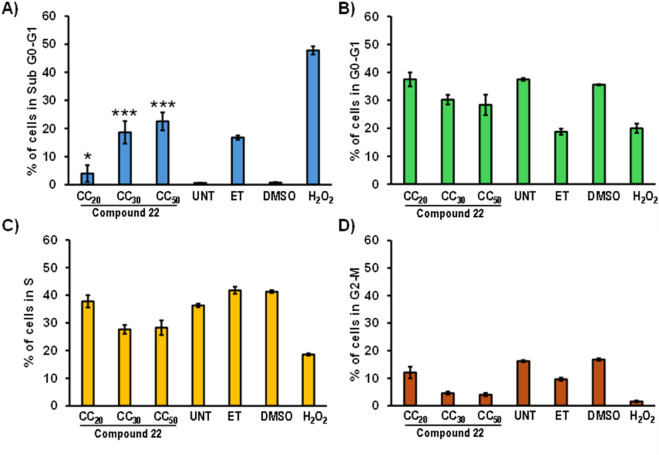
Cell cycle analysis was performed using a NIM-DAPI solution and flow cytometry, followed by 72 h of incubation with **22** at CC_50_, CC_30_, and CC_20,_ DMSO, Etoposide (ET), untreated (UNT), and H_2_O_2_, in CEM cells. Results show the percentages of cells in the **(A)** Sub G0-G1, **(B)** G0-G1, **(C)** S, and **(D)** G2-M phases. The statistical significance of treatment when compared to DMSO control is indicated by the following asterisks: *p < 0.05, **p < 0.01, and ***p < 0.001.

As shown in Figure 5A, compound **22** induces DNA fragmentation in a dose-dependent manner in the sub-G0-G1 phase, most likely due to DNA cleavage during apoptosis. However, no effect was observed at high concentrations across the other stages of the cell cycle (Figures 5B–D). These results suggest that **22** acts as a pro-apoptotic cytotoxic agent rather than as a specific cell-cycle inhibitor.

### 3.7 Docking analysis

Because the CK2 and PIM protein kinases are potential therapeutic targets in T-cell acute lymphoblastic leukemia (T-ALL), molecular docking experiments were performed for all synthesized molecules (Table 4).

**TABLE 4:** *In-silico* estimated free energy of binding by molecular docking.

Compound	PIM-1 PDB:4DTK	CK2α PDB: 4 kW P	Compound	PIM-1 PDB:4DTK	CK2α PDB: 4 kW P
	Estimated free energy**			Estimated free energy**	
2	−6.67	−4.54	15	−6.14	−4.01
3	−6.48	−4.32	16	−7.66	−5.25
4	−7.64	−5.21	17	−8.06	−4.74
5	−7.72	−4.97	18	−8.56	−5.60
6	−7.90	−5.25	19	−8.84	−5.35
7	−8.54	−5.23	20	−9.10	−5.46
8	−8.76	−5.82	21	−9.14	−6.19
9	−8.98	−7.19	22	−10.72	−6.91
10	−9.65	−7.25	23	−8.35	−5.68
11	−10.42	−6.38	24	−8.29	−4.14
12	−8.15	−5.10	25	−8.65	−5.03
13	−7.97	−4.99	TBBI^*^	−8.06	−8.65
14	−8.71	−4.36	​	​	​

* The TBBI, molecule was used as a positive control in all theoretical calculations.

** Free Energy of Binding calculated (kcal⋅mol^-1^).

The results reported in Table 4 show that all estimated binding free energies are higher for the PIM system than for CK2. This indicates that the CK2 mechanism cannot be explained by *in silico* methods. According to molecular docking experiments, the 4DTK-22 complex was the most stable system, with an estimated binding free energy of −10.72 kcal⋅mol^-1^. The experiment results showed compounds **7**, **8**, and **22** exhibited significant cytotoxic activity against the CEM and JURKAT cancer cell lines (as shown in Table 2). Notably, compound **22** exhibited its exceptional cytotoxicity, with a CC_50_ of 2.25 ± 0.03 µM in CEM cells. Given its high level of cytotoxicity, compound **22** was the most active, prompting further exploration of its mechanism of action in CEM cells. Furthermore, compounds **7**, **8**, and **22** demonstrated favorable selectivity for CEM cancer cells compared to the non-cancerous cell line Hs-27 (as presented in Table 3).

**FIGURE 6:**
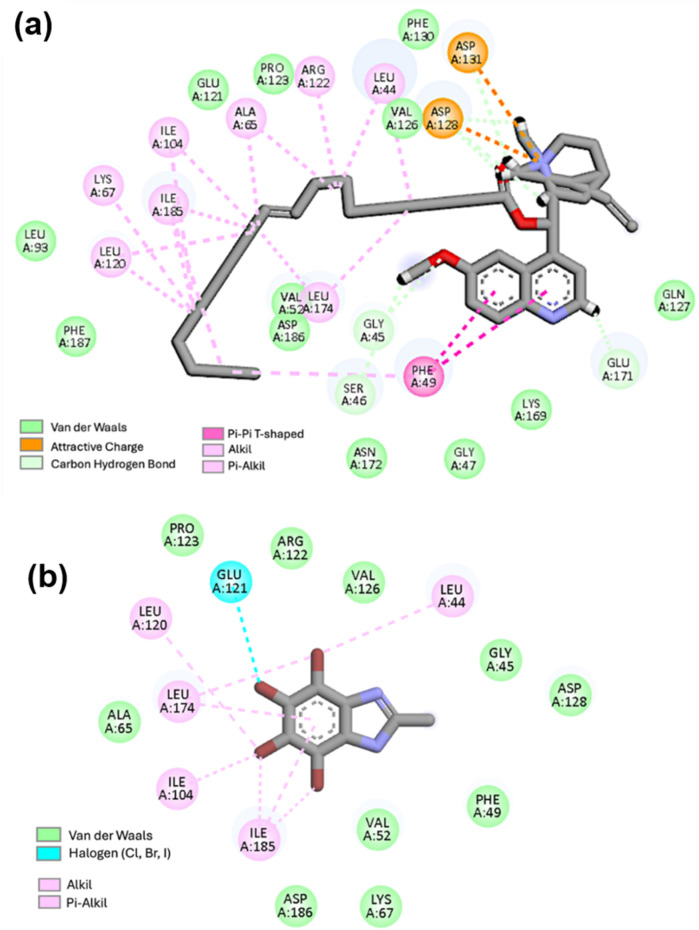
Predicted binding conformations into PIM-1 active site of **(a)** compound **22** and **(b)** TBBI. residues in dark green (VdW interactions), light green (carbon hydrogen bond), cyan (halogen bond), light pink (alkyl and pi-alkyl interaction), dark pink (pi-pi stacking), orange (attractive charge).

Compound **22** demonstrates a robust, multifaceted anchoring mechanism characteristic of a high-molecular-weight ligand occupying a spacious pocket. It engages in an attractive electrostatic interaction with ASP131 (orange in Figure 6), suggesting an ionic interaction (salt bridge) or a strong electrostatic-dipole interaction. Additionally, it forms a carbon-hydrogen bond with GLY45 and GLU171. In the context of hydrophobic interactions (VDW, Alkyl, and Pi-alkyl), the complex establishes an extensive network of these interactions, which is pivotal for stabilizing the ligand’s large apolar structure within the hydrophobic pocket. On the other hand, the protein-TBBI complex is stabilized by hydrophobic interactions and specific halogen contacts. These interactions are typical of smaller, more rigid ligands that interact with only a portion of the active site. Notably, the complex lacks hydrogen bridge interactions but exhibits a specific halogen interaction with GLU A:121 (cyan). This halogen interaction is significant because it is directional and influences the ligand’s orientation. In contrast, hydrophobic interactions primarily rely on alkyl and pi-alkyl interactions, with fewer Van der Waals contacts. This behavior is typical for small ligands with a limited contact surface.

### 3.8 Molecular dynamics study

To comprehensively evaluate the outcomes of molecular docking and inhibition experiments, 100 ns molecular dynamics simulations were conducted for complexes **22**, **8**, **7**, and TBBI. The results of protein-ligand interaction energy, calculated using the MMGBSA methodology, are presented in Table 5. Both molecular docking and molecular dynamics experiments indicate that complexes with the CK2α protein (PDB: 4 kW P) exhibit lower interaction energies than those with the PIM-1 protein (PDB: 4DTK). This suggests that PIM-1, rather than CK2, mediates the inhibition of these molecules. However, it is essential to note that the compound 22 may have distinct biological targets.

**TABLE 5:** Binding energy for PIM-1 and CK2α complexes using molecular dynamics procedure.

Ligand	PIM-1 (PDB: 4DTK)		CK2α (PDB: 4 kW P)	
	Molecular docking *	Molecular dynamics **	Molecular docking *	Molecular dynamics **
7	−8.54	−23.371 ± 2.471	−5.23	−21.794 ± 2.961
8	−8.76	−21.361 ± 3.555	−5.82	−17.355 ± 3.654
22	−10.72	−30.841 ± 4.019	−6.91	−27.789 ± 4.334
TBBI	−8.06	−32.082 ± 2.243	−8.65	−24.697 ± 3.669

* Estimated free energy of binding (kcal⋅mol^-1^).

** Binding energy calculated by MMGBSA (kcal⋅mol^-1^).

The MMGBSA, values indicate that the synthesized molecule with the most potent inhibitory activity is ligand **22**, differing by only 1.24 kcal⋅mol^-1^ from the positive control (TBBI). For ligands **7** and **8**, the differences with the control were 8.71 and 10.72 kcal⋅mol^-1^, respectively. As depicted in Figure 6, complex **22** is stabilized by multiple interactions within the active site.

**FIGURE 7:**
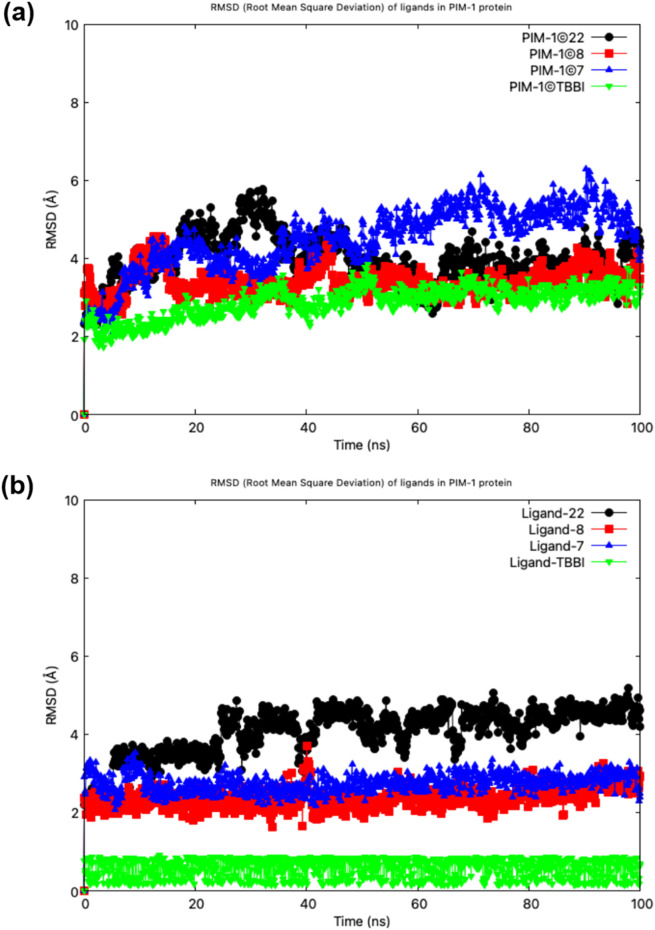
RMSD analysis of **(a)** PIM-1–ligand complexes and **(b)** corresponding ligand stability during molecular dynamics simulations.

Figure 7 illustrates the stability of the protein-ligand complexes as assessed by root-mean-square deviation (RMSD) values. The results demonstrate that complexes **22**, **8**, **7**, and TBBI exhibit remarkable stability over time. This observation suggests a potential connection between these systems *via* their active sites, implying molecular interactions that stabilize the ligands within the PIM-1 protein active site. Notably, the evaluated systems (**22**, **8**, and **7**) exhibit comparable stability to the TBBI control, despite TBBI being smaller and less bulky than the synthesized systems.

**FIGURE 8:**
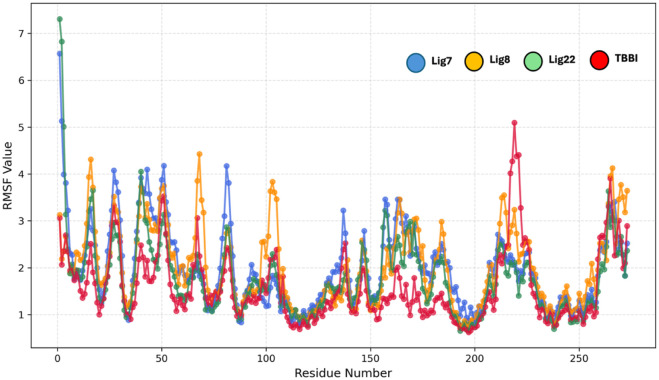
Root mean square fluctuation (RMSF) of PIM-1 residues obtained from molecular dynamics simulations in the presence of ligands **7**, **8**, **22**, and TBBI.

The root-mean-square fluctuation (RMSF) analysis in Figure 8 revealed distinct protein dynamics upon binding to compounds **7**, **8**, **22**, and TBBI. Low RMSF values (below 1.5 Å) indicate stable regions, typically core elements such as α-helices and β-sheets, that are consistently stable across all complexes in segments around residues 100–140 and 240–260. High RMSF peaks (exceeding 3.0 Å) indicate flexible regions, mainly at the *N*-terminal end and in key loop areas, particularly between residues 200 and 220. Compound **22** consistently exhibits the lowest average fluctuations across most of the protein backbone, suggesting it’s the most effective compound in imposing global conformational stability and restricting movement more broadly than the other three ligands.

**FIGURE 9:**
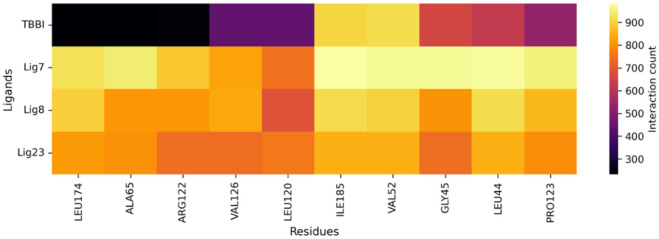
Interaction frequency heat map of PIM-1 active-site residues with ligands **7**, **8**, **22,** and TBBI during molecular dynamics simulations.

A heat map of interactions within a 3 Å radius (Figure 9) shows how frequently ligands engage key residues at the binding site (yellow), indicating more enduring interactions. Compounds **22** and **8** exhibit the most robust interaction profiles, consistently contacting central residues, including LEU174, ALA65, VAL126, LEU120, GLY45, LEU44, and PRO123. This suggests robust anchoring and extensive contacts at the active site. In contrast, the TBBI ligand has the lowest and weakest interaction frequency, with almost all residues, suggesting lower binding affinity and transient contacts. Compounds **22** and **8** establish more effective and stable interactions, which correlate with their potentially enhanced stability. The high frequency of interaction of compound **22** with strongly hydrophobic and aliphatic amino acids (LEU, VAL, and ALA) indicates that hydrophobic interactions and Van der Waals forces drive their binding and stability. Proline (PRO) also provides structural rigidity, explaining the persistence of contact observed in the heat map.

In summary, this study successfully identifies a novel quinine derivative, compound **22**, with potent and selective *in vitro* anti-leukemic activity, and provides compelling preliminary evidence that its mechanism of action involves the induction of intrinsic apoptosis.

However, the research has some limitations that must be acknowledged. The primary constraint is reliance on *in silico* modeling to implicate PIM-1 kinase as the molecular target, without the necessary experimental validation through direct kinase inhibition or cellular target-engagement assays. Furthermore, the mechanistic link between the proposed PIM-1 inhibition and the observed mitochondrial depolarization remains to be fully elucidated. Crucially, the absence of *in vivo* efficacy and pharmacokinetic studies means the therapeutic potential and safety profile of compound **22** in a living system are completely unknown.

To address these limitations and advance this promising lead, future work should prioritize three key areas. First, target validation studies are essential, including biochemical PIM-1 kinase assays and genetic approaches *(e.g.*, PIM-1 knockdown/overexpression) to confirm that the compound’s cytotoxic effects are indeed PIM-1-dependent. Second, detailed mechanistic studies should map the signaling pathway from PIM-1 inhibition to mitochondrial dysfunction by examining the phosphorylation status of known PIM-1 substrates, such as Bad (Sharma et al., 2024). Third, and most critically, compound **22** must be evaluated in preclinical *in vivo* models. This would involve assessing its pharmacokinetic properties, tolerability, and anti-tumor efficacy in a murine xenograft model of acute lymphoblastic leukemia. Such studies would transform compound **22** from a mechanistically interesting *in vitro* hit into a credible lead candidate for further anticancer drug development.

## 4 Conclusion

In conclusion, among the synthesized quinine derivatives, compound **22** exhibited potent cytotoxicity against various cancer cell lines, including acute lymphoblastic leukemia and acute T-cell leukemia. Compound **22** induced apoptosis, as evidenced by PS externalization, caspase-3/7 activation, mitochondrial depolarization, and DNA fragmentation. In addition, compound **22** exhibited a favorable selective cytotoxicity towards leukemia cells. Thus, the novel PIM-1 kinase inhibitor demonstrated attractive anti-leukemic cytotoxicity and a suitable mechanism of action, making it a good candidate for its evaluation as an anticancer drug in an animal model.

## Data Availability

The original contributions presented in the study are included in the article/Supplementary Material, further inquiries can be directed to the corresponding authors.

## References

[B1] Abroshan H. Akbarzadeh H. Parsafar G. A. (2010). Molecular dynamics simulation and MM–PBSA calculations of sickle cell hemoglobin in dimer form with Val, Trp, or Phe at the lateral contact. J. Phys. Org. Chem. 23, 866–877. 10.1002/poc.1679

[B2] Adasme M. F. Linnemann K. L. Bolz S. N. Kaiser F. Salentin S. Haupt V. J. (2021). PLIP 2021: expanding the scope of the protein–ligand interaction profiler to DNA and RNA. Nucleic Acids Res. 49, W530–W534. 10.1093/nar/gkab294 33950214 PMC8262720

[B3] Aishwarya S. Torres G. C. Lopez-Saenz J. A. Gutierrez D. A. Kumar S. Madarakhandi A. (2025). Synthesis of novel pyridazine and pyrimidine linked pyrazole derivatives as DNA ligase 1 and IV inhibitors that induce apoptosis. Chem. Biol. Interact. 414, 111509. 10.1016/j.cbi.2025.111509 40221125 PMC12083462

[B4] Antika L. D. Triana D. Ernawati T. (2020). Antimicrobial activity of quinine derivatives against human pathogenic bacteria. IOP Conf. Ser. Earth Environ. Sci. 462, 012006. 10.1088/1755-1315/462/1/012006

[B5] Averill-Bates D. (2024). Reactive oxygen species and cell signaling. Review. Biochim. Biophys. Acta - Mol. Cell Res. 1871, 119573. 10.1016/j.bbamcr.2023.119573 37949302

[B6] Aymard G. A. (2019). A brief outline on current taxonomical and nomenclatural aspects of the genus Cinchona (Rubiaceae-Cinchoneae). Rev. la Acad. Colomb. Ciencias Exactas, Físicas Nat 43, 234–241.

[B7] Basrani S. Patil S. Chougule S. Kotalagi T. Yankanchi S. Karuppayil S. M. (2025). Repurposing of quinine as an antifungal antibiotic: identification of molecular targets in Candida albicans. Folia Microbiol. (Praha). 10.1007/s12223-025-01281-5 40483339

[B8] Bray F. Laversanne M. Sung H. Ferlay J. Siegel R. L. Soerjomataram I. (2024). Global cancer statistics 2022: GLOBOCAN estimates of incidence and mortality worldwide for 36 cancers in 185 countries. Ca. Cancer J. Clin. 74, 229–263. 10.3322/caac.21834 38572751

[B9] Brooks B. R. Brooks C. L. Mackerell A. D. Nilsson L. Petrella R. J. Roux B. (2009). CHARMM: the biomolecular simulation program. J. Comput. Chem. 30, 1545–1614. 10.1002/jcc.21287 19444816 PMC2810661

[B10] Cai Z. Luo Q. Zhang Y. Meng R. Tian M. (2025). CK2 in the spotlight: decoding its role in hematological malignancies and therapeutic applications. Discov. Oncol. 16, 965. 10.1007/s12672-025-02797-5 40445457 PMC12125434

[B11] Cen H. Mao F. Aronchik I. Fuentes R. J. Firestone G. L. (2008). DEVD‐NucView488: a novel class of enzyme substrates for real‐time detection of caspase‐3 activity in live cells. FASEB J. 22, 2243–2252. 10.1096/fj.07-099234 18263700

[B12] Chawla R. Vaidya A. (2024). Anticancer mechanism of quinoline based compounds for cancer therapy. Int. J. Med. Pharm. Heal. Sci. 1, 65–79. 10.62946/IJMPHS/1.2.65-79

[B13] Cozza G. Girardi C. Ranchio A. Lolli G. Sarno S. Orzeszko A. (2014). Cell-permeable dual inhibitors of protein kinases CK2 and PIM-1: structural features and pharmacological potential. Cell. Mol. Life Sci. 71, 3173–3185. 10.1007/s00018-013-1552-5 24442476 PMC11113908

[B14] Echeverría J. Urzúa A. Sanhueza L. Wilkens M. (2017). Enhanced antibacterial activity of Ent-Labdane derivatives of Salvic acid (7α-Hydroxy-8(17)-ent-Labden-15-Oic acid): effect of lipophilicity and the hydrogen bonding role in bacterial membrane interaction. Molecules 22 (7), 1039. 10.3390/molecules22071039 28644410 PMC6152121

[B15] El-Miligy M. M. M. Abdelaziz M. E. Fahmy S. M. Ibrahim T. M. Abu-Serie M. M. Mahran M. A. (2023). Discovery of new pyridine-quinoline hybrids as competitive and non-competitive PIM-1 kinase inhibitors with apoptosis induction and caspase 3/7 activation capabilities. J. Enzyme Inhib. Med. Chem. 38, 2152810. 10.1080/14756366.2022.2152810 36629075 PMC9848351

[B16] Eskandari E. Eaves C. J. (2022). Paradoxical roles of caspase-3 in regulating cell survival, proliferation, and tumorigenesis. J. Cell Biol. 221, e202201159. 10.1083/jcb.202201159 35551578 PMC9106709

[B17] Fadok V. A. Bratton D. L. Frasch S. C. Warner M. L. Henson P. M. (1998). The role of phosphatidylserine in recognition of apoptotic cells by phagocytes. Cell Death Differ. 5, 551–562. 10.1038/sj.cdd.4400404 10200509

[B18] Fattahi N. Shahbazi M.-A. Maleki A. Hamidi M. Ramazani A. Santos H. A. (2020). Emerging insights on drug delivery by fatty acid mediated synthesis of lipophilic prodrugs as novel nanomedicines. J. Control. Release 326, 556–598. 10.1016/j.jconrel.2020.07.012 32726650

[B19] Ghani M. U. Shi J. Du Y. Zhong L. Cui H. (2024). A comprehensive review on the dynamics of protein kinase CK2 in cancer development and optimizing therapeutic strategies. Int. J. Biol. Macromol. 280, 135814. 10.1016/j.ijbiomac.2024.135814 39306165

[B20] Götz A. W. Williamson M. J. Xu D. Poole D. Le Grand S. Walker R. C. (2012). Routine microsecond molecular dynamics simulations with AMBER on GPUs. 1. Generalized born. J. Chem. Theory Comput. 8, 1542–1555. 10.1021/ct200909j 22582031 PMC3348677

[B21] Große M. Ruetalo N. Layer M. Hu D. Businger R. Rheber S. (2021). Quinine inhibits infection of human cell lines with SARS-CoV-2. Viruses 13, 647. 10.3390/v13040647 33918670 PMC8069458

[B22] Gutierrez D. A. Contreras L. Villanueva P. J. Borrego E. A. Morán-Santibañez K. Hess J. D. (2022). Identification of a potent cytotoxic pyrazole with anti-breast cancer activity that alters multiple pathways. Cells 11, 254. 10.3390/cells11020254 35053370 PMC8773755

[B23] Hariyanti H. Mauludin R. Sumirtapura Y. C. Kurniati N. F. (2022). A review: pharmacological activities of quinoline Alkaloid of Cinchona sp. Biointerface Res. Appl. Chem. 13, 319. 10.33263/BRIAC134.319

[B24] Hayes J. M. Archontis G. (2012). MM-GB (PB) SA calculations of protein-ligand binding free energies. Mol. Dyn. Synth. Biol. Macromol. 11, 171–190.

[B25] Huang L. Yang J. Wang T. Gao J. Xu D. (2022). Engineering of small-molecule lipidic prodrugs as novel nanomedicines for enhanced drug delivery. J. Nanobiotechnology 20, 49. 10.1186/s12951-022-01257-4 35073914 PMC8785568

[B26] Humphrey W. Dalke A. Schulten K. (1996). VMD: visual molecular dynamics. J. Mol. Graph. 14, 33–38. 10.1016/0263-7855(96)00018-5 8744570

[B27] Joyce M. D. Jennings M. C. Santiago C. N. Fletcher M. H. Wuest W. M. Minbiole K. P. (2016). Natural product-derived quaternary ammonium compounds with potent antimicrobial activity. J. Antibiot. (Tokyo). 69, 344–347. 10.1038/ja.2015.107 26577453

[B28] Kato E. Oshima S. (2023). Association of bitter taste receptors with obesity and diabetes and their role in related tissues. Receptors 2, 251–263. 10.3390/receptors2040017

[B29] Kawai R. Yada S. Yoshimura T. (2021). Adsorption and aggregation behavior of mixtures of quaternary-ammonium-salt-type amphiphilic compounds with fluorinated counterions and surfactants. Langmuir 37, 11330–11337. 10.1021/acs.langmuir.1c01912 34520217

[B30] Krishnaveni M. Suresh K. (2015). Induction of apoptosis by quinine in human laryngeal carcinoma cell line. Int. J. Curr. Res. Acad. Rev. 3, 169–178.

[B31] Kwak A.-W. Kim W.-K. Lee S.-O. Yoon G. Cho S.-S. Kim K.-T. (2023). Licochalcone B induces ROS-Dependent apoptosis in oxaliplatin-resistant colorectal cancer cells *via* p38/JNK MAPK signaling. Antioxidants 12, 656. 10.3390/antiox12030656 36978904 PMC10045364

[B32] Lauwerys L. Beroske L. Solania A. Vangestel C. Miranda A. Van Giel N. (2024). Development of caspase-3-selective activity-based probes for PET imaging of apoptosis. EJNMMI Radiopharm. Chem. 9, 58. 10.1186/s41181-024-00291-x 39117920 PMC11310375

[B33] Lema C. Varela-Ramirez A. Aguilera R. J. (2011). Differential nuclear staining assay for high-throughput screening to identify cytotoxic compounds. Curr. Cell. Biochem. 1, 1–14. 27042697 PMC4816492

[B34] Liu W. Qi Y. Liu L. Tang Y. Wei J. Zhou L. (2016). Suppression of tumor cell proliferation by quinine *via* the inhibition of the tumor necrosis factor receptor-associated factor 6-AKT interaction. Mol. Med. Rep. 14, 2171–2179. 10.3892/mmr.2016.5492 27430155

[B35] McNeice P. Vallana F. M. F. Coles S. J. Horton P. N. Marr P. C. Seddon K. R. (2020). Quinine based ionic liquids: a tonic for base instability. J. Mol. Liq. 297, 111773. 10.1016/j.molliq.2019.111773

[B36] Mohammadi M. R. Rezaei Niaraki E. (2023). Antibacterial, antiviral, and antifungal activities of quinine and its derivatives: a narrative mini-review. Micro Nano Bio Asp. 2, 1–6. 10.22034/mnba.2023.399582.1034

[B37] Molnár I. G. Gilmour R. (2016). Catalytic difluorination of olefins. J. Am. Chem. Soc. 138, 5004–5007. 10.1021/jacs.6b01183 26978593

[B38] Morris G. M. Goodsell D. S. Halliday R. S. Huey R. Hart W. E. Belew R. K. (1998). Automated docking using a Lamarckian genetic algorithm and an empirical binding free energy function. J. Comput. Chem. 19, 1639–1662. 10.1002/(SICI)1096-987X(19981115)19:14<1639::AID-JCC10>3.0.CO;2-B

[B39] Morris G. M. Ruth H. Lindstrom W. Sanner M. F. Belew R. K. Goodsell D. S. (2009). Software news and updates AutoDock4 and AutoDockTools4: automated docking with selective receptor flexibility. J. Comput. Chem. 30, 2785–2791. 10.1002/jcc.21256 19399780 PMC2760638

[B40] Mukusheva G. K. Zhasymbekova A. R. Seidakhmetova R. B. Nurkenov O. A. Akishina E. A. Petkevich S. K. (2022). Quinine esters with 1,2-Azole, pyridine and adamantane fragments. Molecules 27, 3476. 10.3390/molecules27113476 35684414 PMC9182173

[B41] Neria E. Fischer S. Karplus M. (1996). Simulation of activation free energies in molecular systems. J. Chem. Phys. 105, 1902–1921. 10.1063/1.472061

[B42] Pagliaro L. Chen S.-J. Herranz D. Mecucci C. Harrison C. J. Mullighan C. G. (2024). Acute lymphoblastic leukaemia. Nat. Rev. Dis. Prim. 10, 41. 10.1038/s41572-024-00525-x 38871740

[B43] Pan T. Zhang J. Wang X. Song Y. (2025). Global burden and trends of hematologic malignancies based on global cancer observatory 2022 and global burden of disease 2021. Exp. Hematol. Oncol. 14, 98. 10.1186/s40164-025-00684-x 40676693 PMC12273037

[B44] Parra-Soto S. Petermann-Rocha F. Martínez-Sanguinetti M. A. Leiva-Ordeñez A. M. Troncoso-Pantoja C. Ulloa N. (2020). Cáncer en Chile y en el mundo: una mirada actual y su futuro escenario epidemiológico. Rev. Med. Chil. 148, 1489–1495. 10.4067/S0034-98872020001001489 33844720

[B45] Phillips J. C. Braun R. Wang W. Gumbart J. Tajkhorshid E. Villa E. (2005). Scalable molecular dynamics with NAMD. J. Comput. Chem. 26, 1781–1802. 10.1002/jcc.20289 16222654 PMC2486339

[B46] Phillips J. C. Hardy D. J. Maia J. D. C. Stone J. E. Ribeiro J. V. Bernardi R. C. (2020). Scalable molecular dynamics on CPU and GPU architectures with NAMD. J. Chem. Phys. 153, 044130. 10.1063/5.0014475 32752662 PMC7395834

[B47] Pósa S. P. Dargó G. Nagy S. Kisszékelyi P. Garádi Z. Hámori L. (2022). Cytotoxicity of cinchona alkaloid organocatalysts against MES-SA and MES-SA/Dx5 multidrug-resistant uterine sarcoma cell lines. Bioorg. Med. Chem. 67, 116855. 10.1016/j.bmc.2022.116855 35640378

[B48] Pradhan N. Akhtar N. Nath B. Peña-García J. Gupta A. Pérez-Sánchez H. (2021). Inhibition of immunosuppressive indoleamine 2, 3-dioxygenase by targeting the heme and apo-form. Chem. Commun. 57, 395–398. 10.1039/d0cc06942f 33326535

[B49] Robles-Escajeda E. Das U. Ortega N. M. Parra K. Francia G. Dimmock J. R. (2016). A novel curcumin-like dienone induces apoptosis in triple-negative breast cancer cells. Cell. Oncol. 39, 265–277. 10.1007/s13402-016-0272-x 26920032 PMC4899127

[B50] Santos F. A. Rao V. S. N. (1998). A study of the anti-pyretic effect of quinine, an alkaloid effective against cerebral malaria, on fever induced by bacterial endotoxin and yeast in rats. J. Pharm. Pharmacol. 50, 225–229. 10.1111/j.2042-7158.1998.tb06180.x 9530992

[B51] Saxena A. Majee S. Ray D. Saha B. (2024). Inhibition of cancer cells by Quinoline-Based compounds: a review with mechanistic insights. Bioorg. Med. Chem. 103, 117681. 10.1016/j.bmc.2024.117681 38492541

[B52] Sharma A. Dubey R. Gupta S. Asati V. Kumar V. Kumar D. (2024). PIM kinase inhibitors: an updated patent review (2016-present). Expert Opin. Ther. Pat. 34, 365–382. 10.1080/13543776.2024.2365411 38842051

[B53] Sharma A. Dubey R. Asati V. Baweja G. S. Gupta S. Asati V. (2025). Assessment of structural and activity-related contributions of various PIM-1 kinase inhibitors in the treatment of leukemia and prostate cancer. Mol. Divers. 29, 4999–5031. 10.1007/s11030-023-10795-4 38642309

[B54] Solary E. Velay I. Chauffert B. Bidan J.-M. Caillot D. Dumas M. (1991). Sufficient levels of quinine in the serum circumvent the multidrug resistance of the human leukemic cell line K562/ADM. Cancer 68, 1714–1719. 10.1002/1097-0142(19911015)68:8<1714::AID-CNCR2820680811>3.0.CO;2-2 1913513

[B55] Takács A. Jessen M. Lajkó E. Szász Z. Kalabay M. Csámpai A. (2025). Quinine-chalcone hybrids as potent inhibitors of P-glycoprotein with apoptotic effects on EBC-1 cells. Biomed. Pharmacother. 187, 118076. 10.1016/j.biopha.2025.118076 40267640

[B56] Villalobos C. Ferrer-Rosende P. Cavallera C. Cavada G. Manríquez M. Quirland C. (2023). Distribución geográfica de la incidencia de cáncer de beneficiarios de un convenio de atención oncológica en Chile. Rev. Med. Chil. 151, 340–348. 10.4067/s0034-98872023000300340 38293879

[B57] Wang J. Wolf R. M. Caldwell J. W. Kollman P. A. Case D. A. (2004). Development and testing of a general amber force field. J. Comput. Chem. 25, 1157–1174. 10.1002/jcc.20035 15116359

[B58] Wińska P. Wielechowska M. Milewski Ł. Siedlecki P. Łukowska-Chojnacka E. (2025). Pro-apoptotic activity of 1-(4,5,6,7-Tetrabromo-1H-benzimidazol-1-yl)propan-2-one, an intracellular inhibitor of PIM-1 kinase in acute lymphoblastic leukemia and breast cancer cells. Int. J. Mol. Sci. 26, 5897. 10.3390/ijms26125897 40565356 PMC12193579

[B59] Wu X. Lin C. Wang H. Gao J. Chen S. Zhou Z. (2025). Connectivity-map unveils Gemcitabine’s efficacy in overcoming nelarabine resistance in T-cell acute lymphoblastic leukemia. Biochem. Biophys. Res. Commun. 769, 151971. 10.1016/j.bbrc.2025.151971 40354678

[B60] Xiong F.-R. Zhu J.-J. Zhu X.-R. Lu J. Yang J.-K. (2025). Low-dose quinine targets KCNH6 to potentiate glucose-induced insulin secretion. J. Mol. Cell Biol. 16, mjae051. 10.1093/jmcb/mjae051 39848918 PMC12120441

