# Supplementary material for: A novel quinine derivative as a PIM-1 kinase inhibitor induces apoptosis via mitochondrial depolarization with selective cytotoxicity in acute lymphoblastic leukemia cells
Source: Front Pharmacol. 2026 Apr 21;17:1799674. doi: 10.3389/fphar.2026.1799674 (PMC13139020; doi:10.3389/fphar.2026.1799674)
Supplement: Supplementary file 1 [file Supplementaryfile1.docx]

**SUPPLEMENTARY MATERIAL**

**
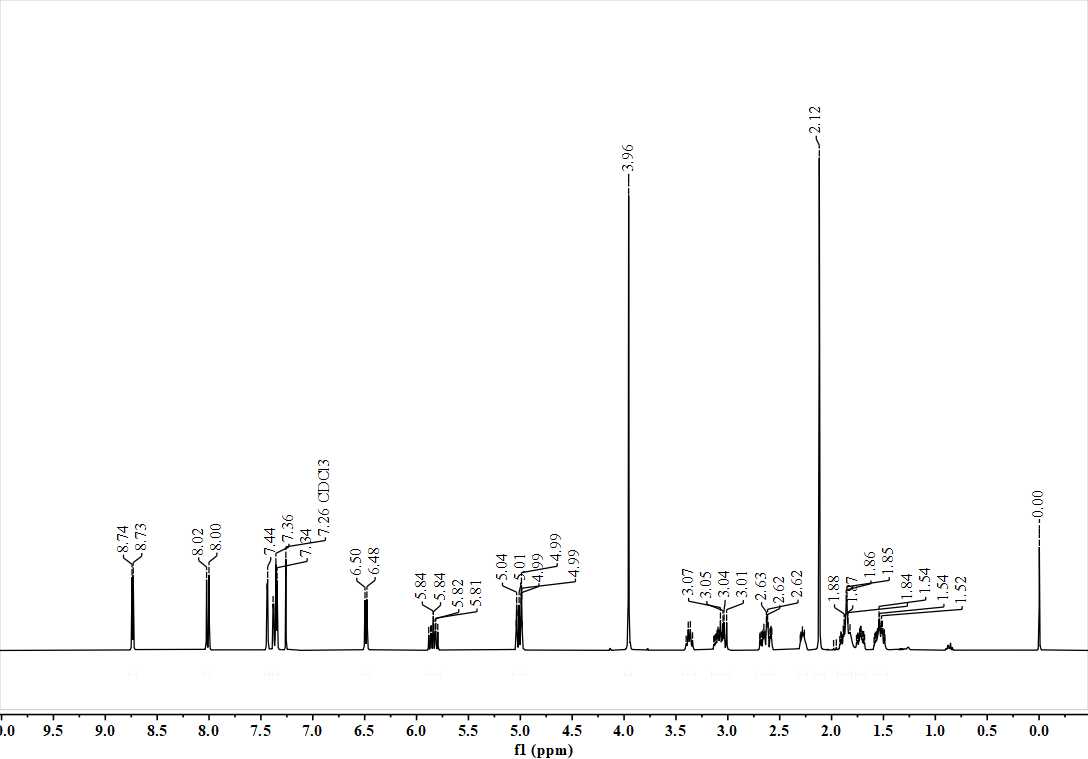
**

**Figure S1.** ^1^H-NMR of (*R*)-(6-methoxyquinolin-4-yl) ((1*S*,2*S*,4*S*,5*R*)-5-vinylquinuclidin-2-yl) methyl acetate **(2)**

**
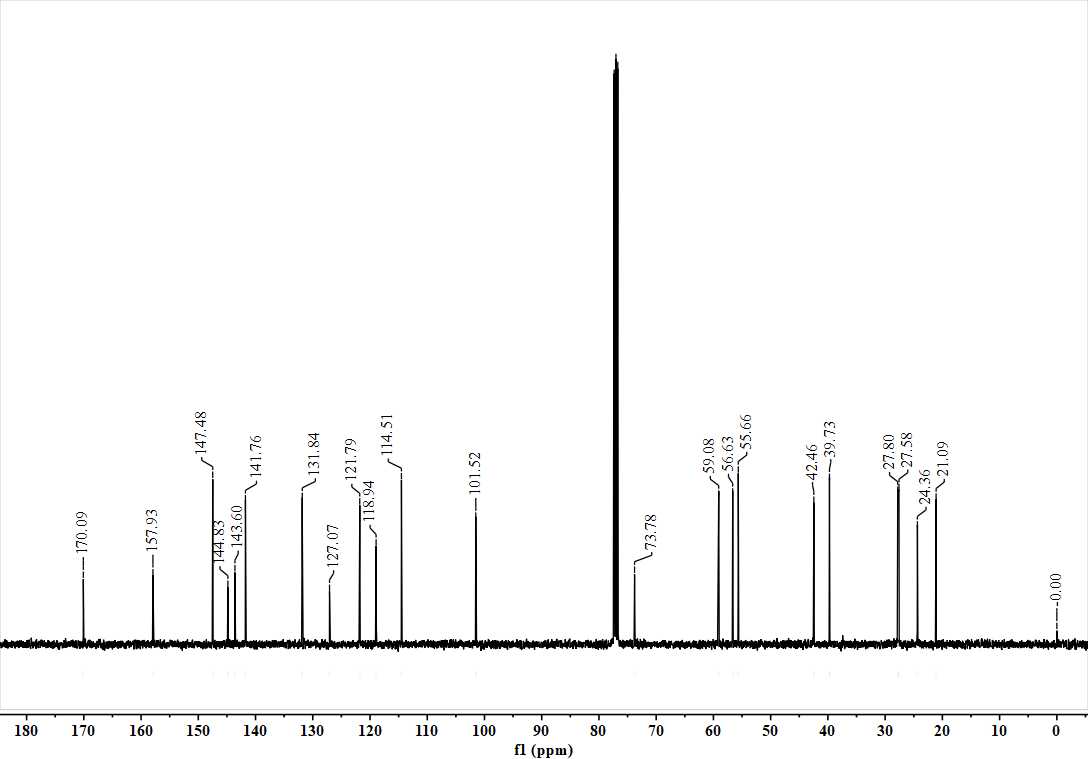
**

**Figure S2.** ^13^C-NMR of (*R*)-(6-methoxyquinolin-4-yl) ((1*S*,2*S*,4*S*,5*R*)-5-vinylquinuclidin-2-yl) methyl acetate **(2)**

***
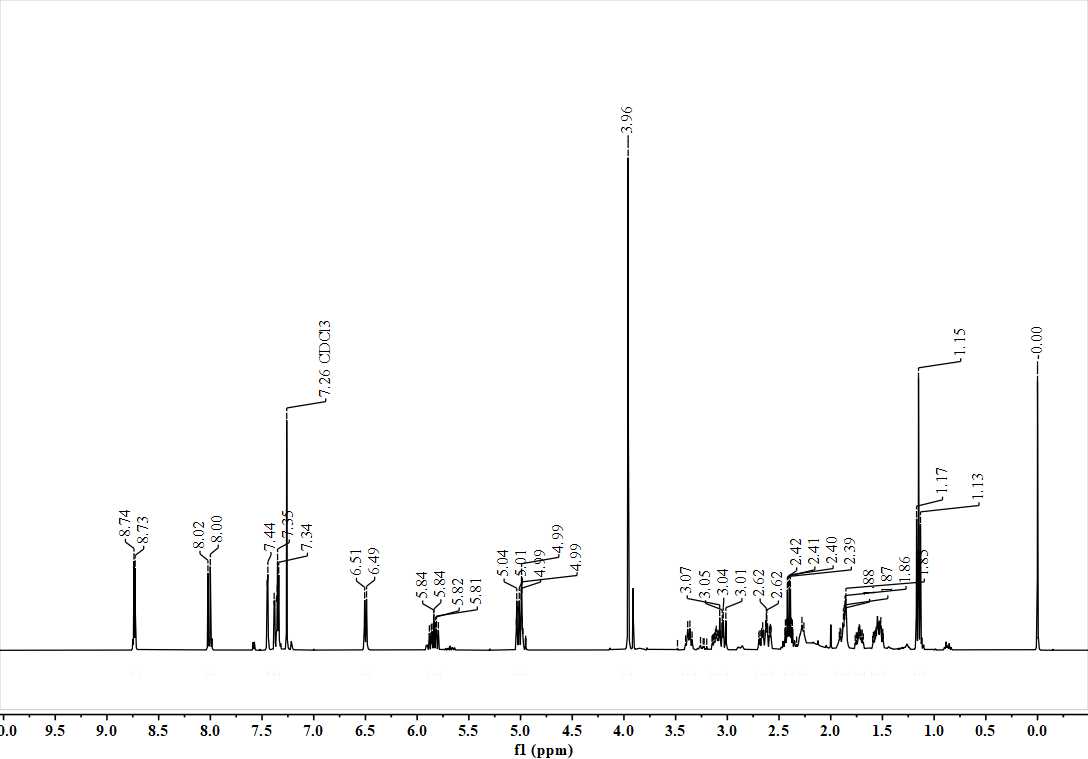
***

**Figure S3.** ^1^H-NMR of (*R*)-(6-methoxyquinolin-4-yl) (1*S*,2*S*,4*S*,5*R*)-5-vinylquinuclidin-2-yl) methyl propionate (**3**).

***
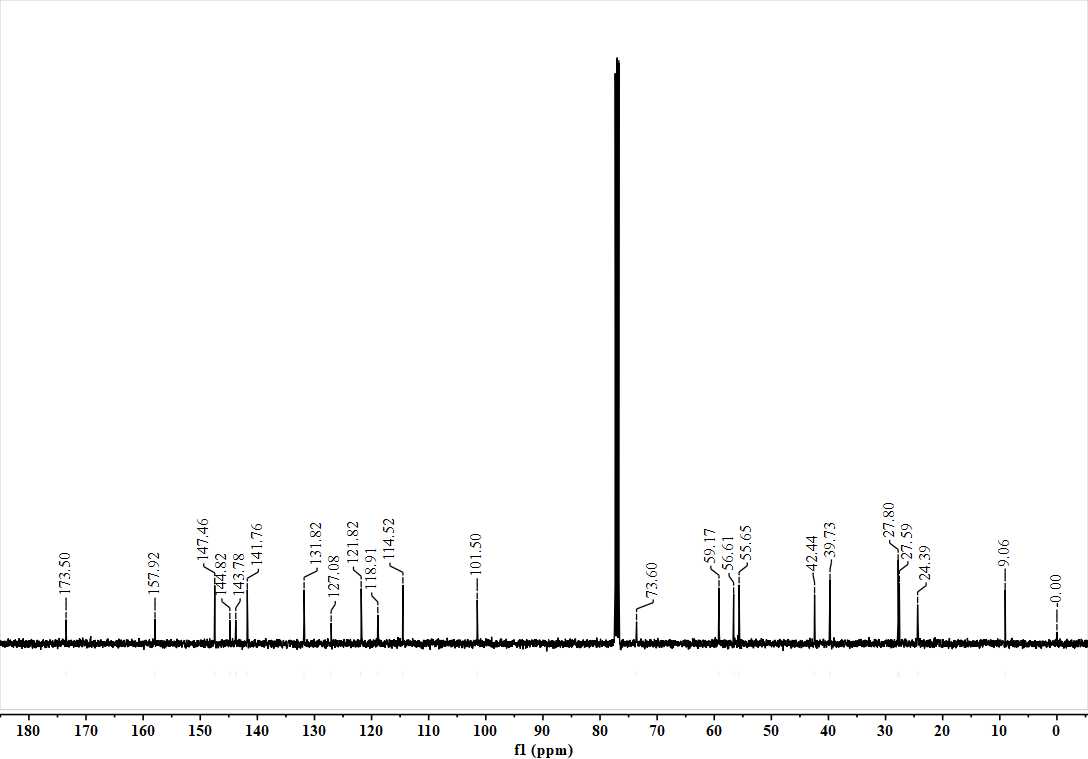
***

**Figure S4.** ^13^C-NMR of (*R*)-(6-methoxyquinolin-4-yl) (1*S*,2*S*,4*S*,5*R*)-5-vinylquinuclidin-2-yl) methyl propionate (**3**)

*
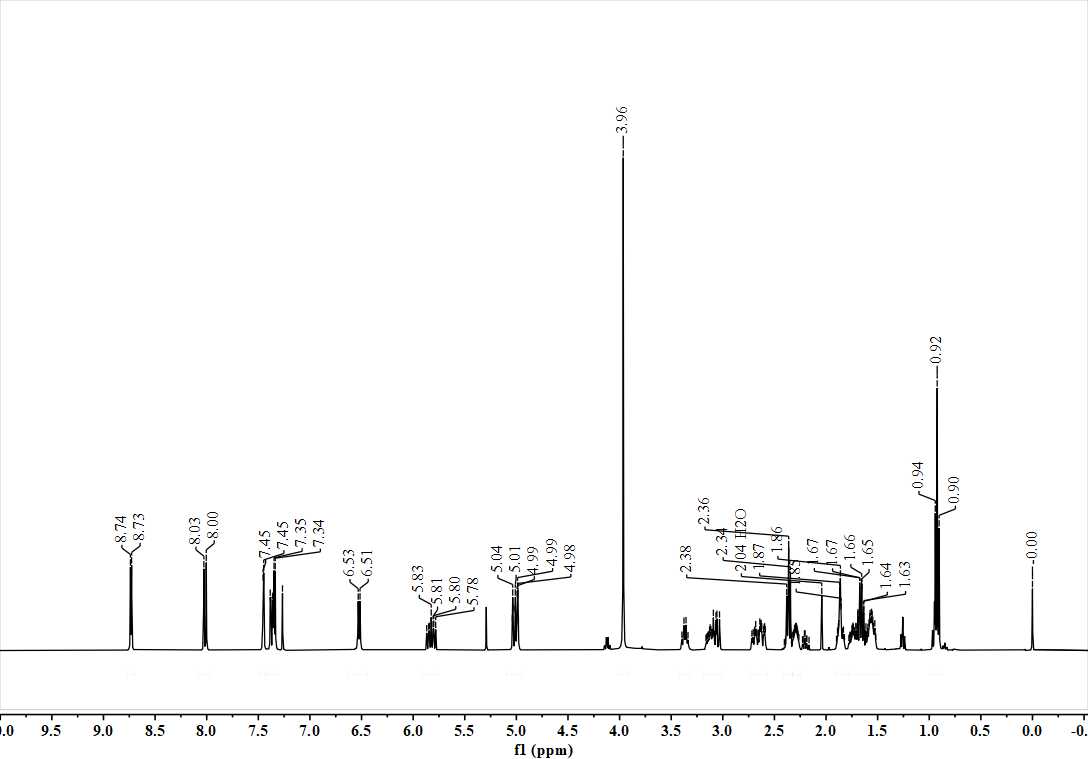
*

**Figure S5.** ^1^H-NMR of (*R*)-(6-methoxyquinolin-4-yl) (1*S*,2*S*,4*S*,5*R*)-5-vinylquinuclidin-2-yl) methyl butyrate (**4**)

***
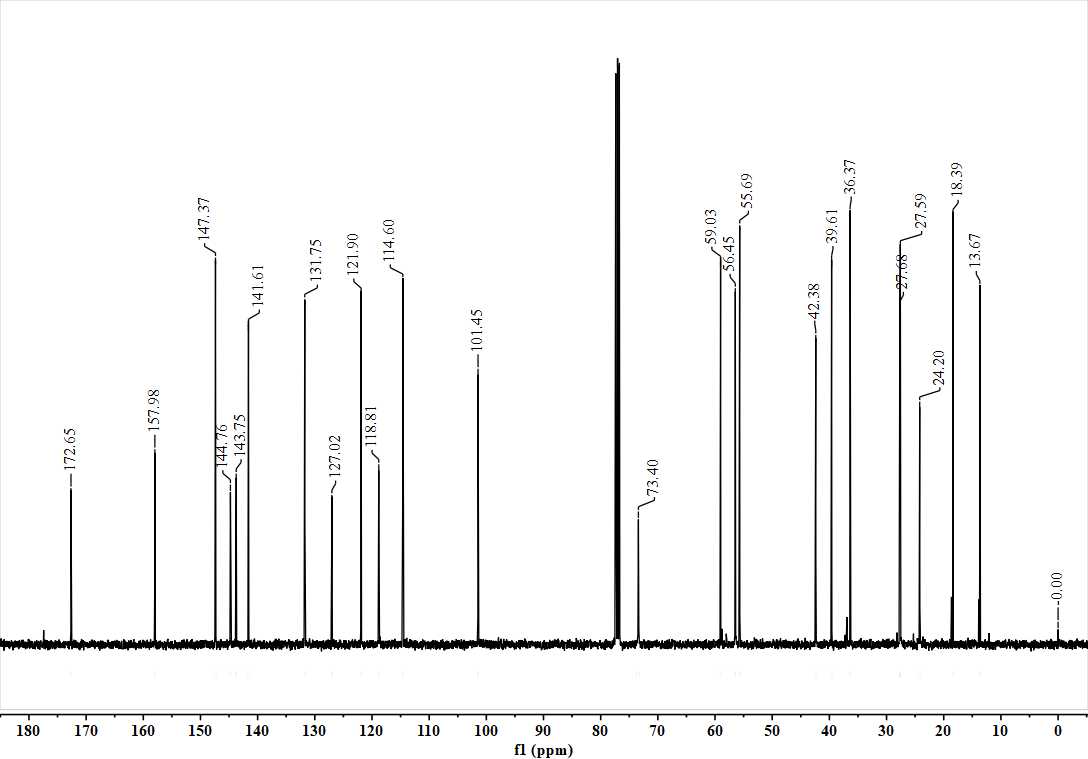
***

**Figure S4.** ^13^C-NMR of (R)-(6-methoxyquinolin-4-yl) (1S,2S,4S,5R)-5-vinylquinuclidin-2-yl) methyl butyrate (**4**).

*
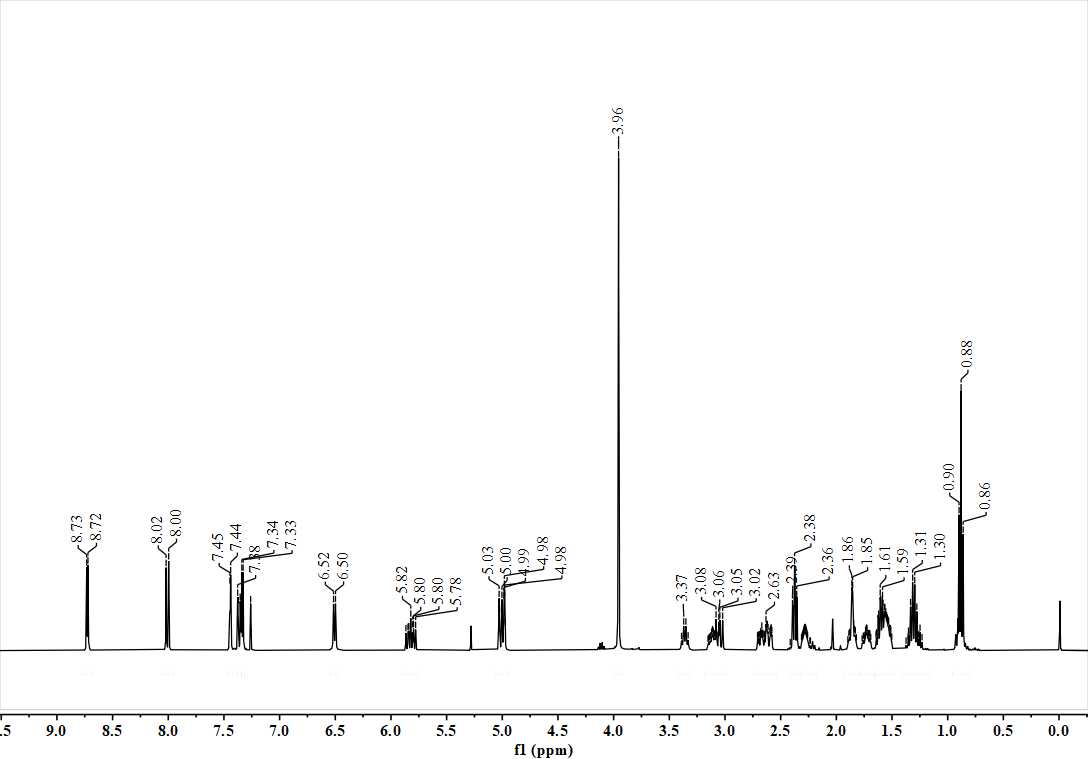
*

**Figure S5.** ^1^H-NMR of (*R*)-(6-methoxyquinolin-4-yl) (1*S*,2*S*,4*S*,5*R*)-5-vinylquinuclidin-2-yl) methyl pentanoate (**5**).

*
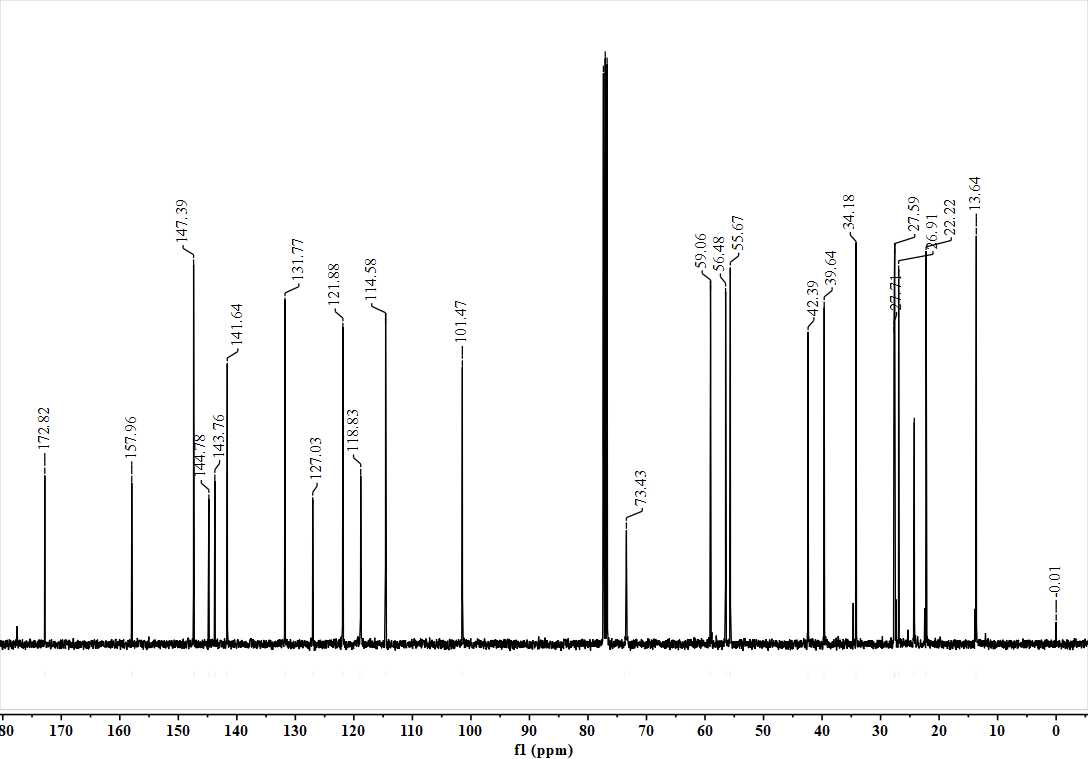
*

**Figure S6.** ^13^C-NMR of (*R*)-(6-methoxyquinolin-4-yl) (1*S*,2*S*,4*S*,5*R*)-5-vinylquinuclidin-2-yl) methyl pentanoate (**5**).

*
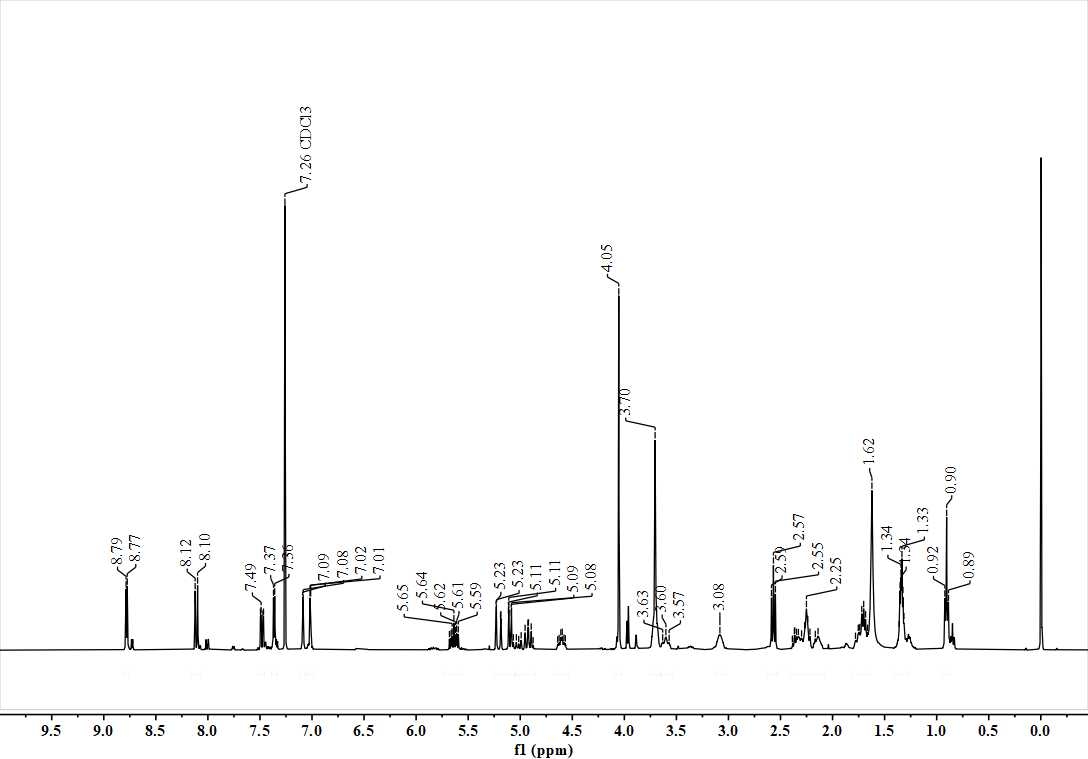
*

**Figure S7.** ^1^H-NMR of (*R*)-(6-methoxyquinolin-4-yl) (1*S*,2*S*,4*S*,5*R*)-5-vinylquinuclidin-2-yl) methyl hexanoate **(6).**

*
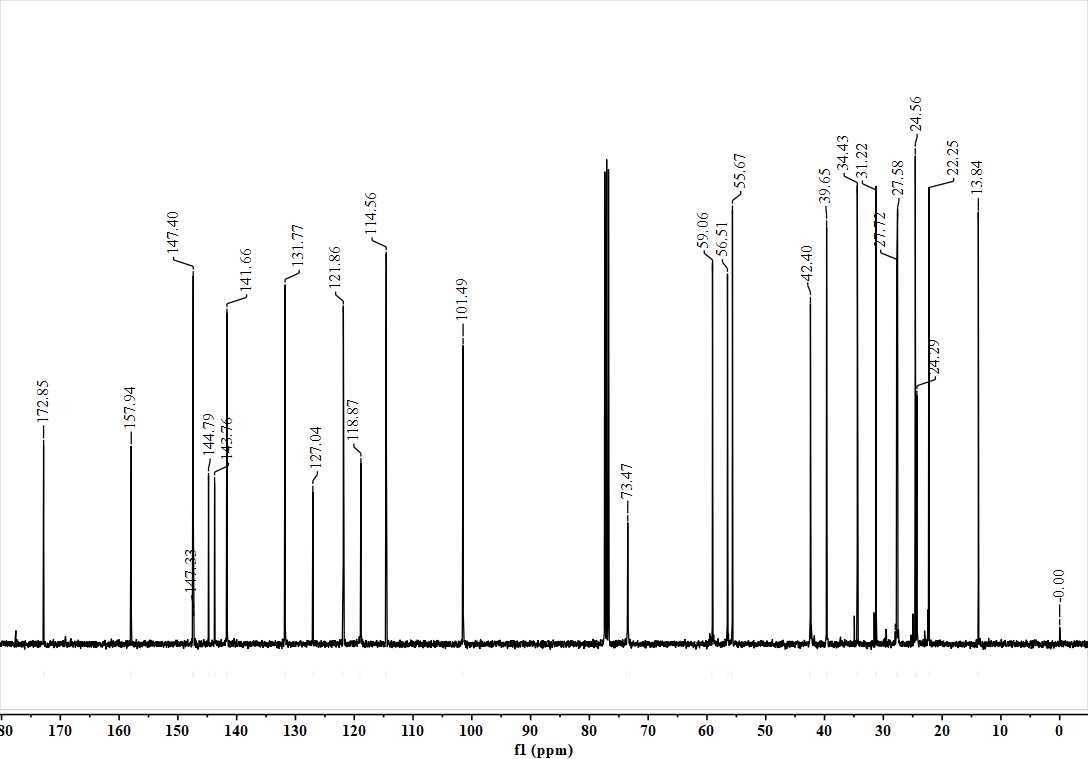
*

**Figure S8.** ^13^C-NMR of (*R*)-(6-methoxyquinolin-4-yl) (1*S*,2*S*,4*S*,5*R*)-5-vinylquinuclidin-2-yl) methyl hexanoate **(6).**

*
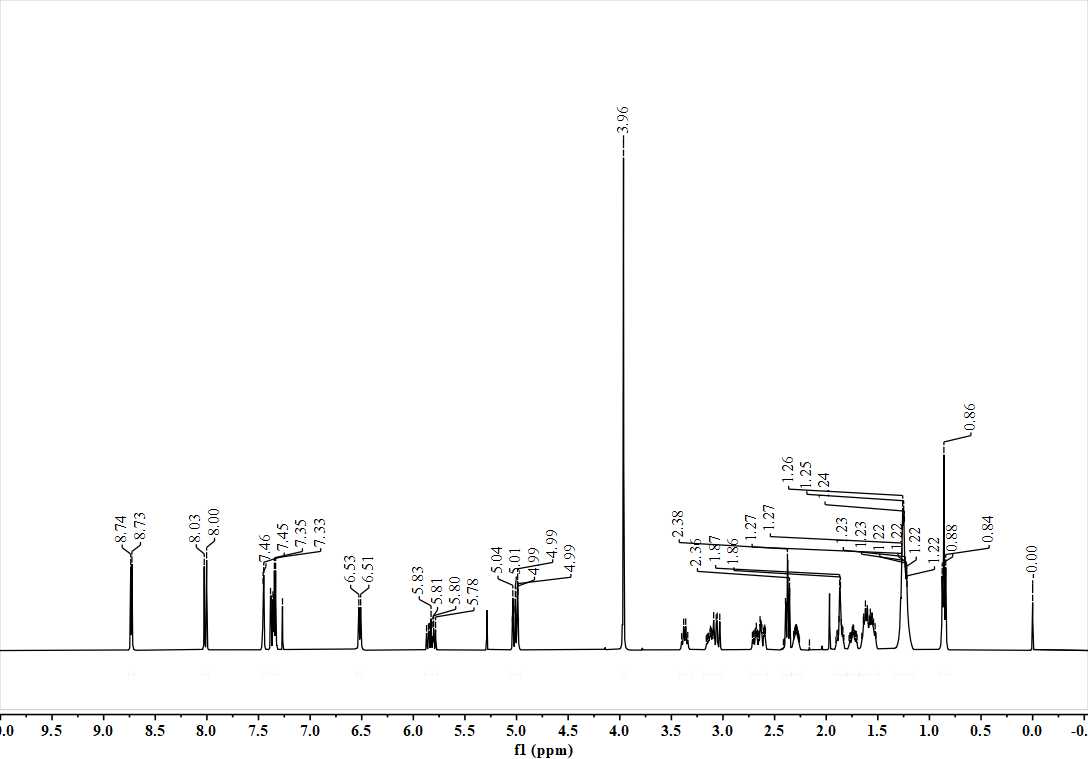
*

**Figure S9.** ^1^H-NMR of (R)-(6-methoxyquinolin-4-yl) (1S,2S,4S,5R)-5-vinylquinuclidin-2-yl) methyl octanoate (**7**).

*
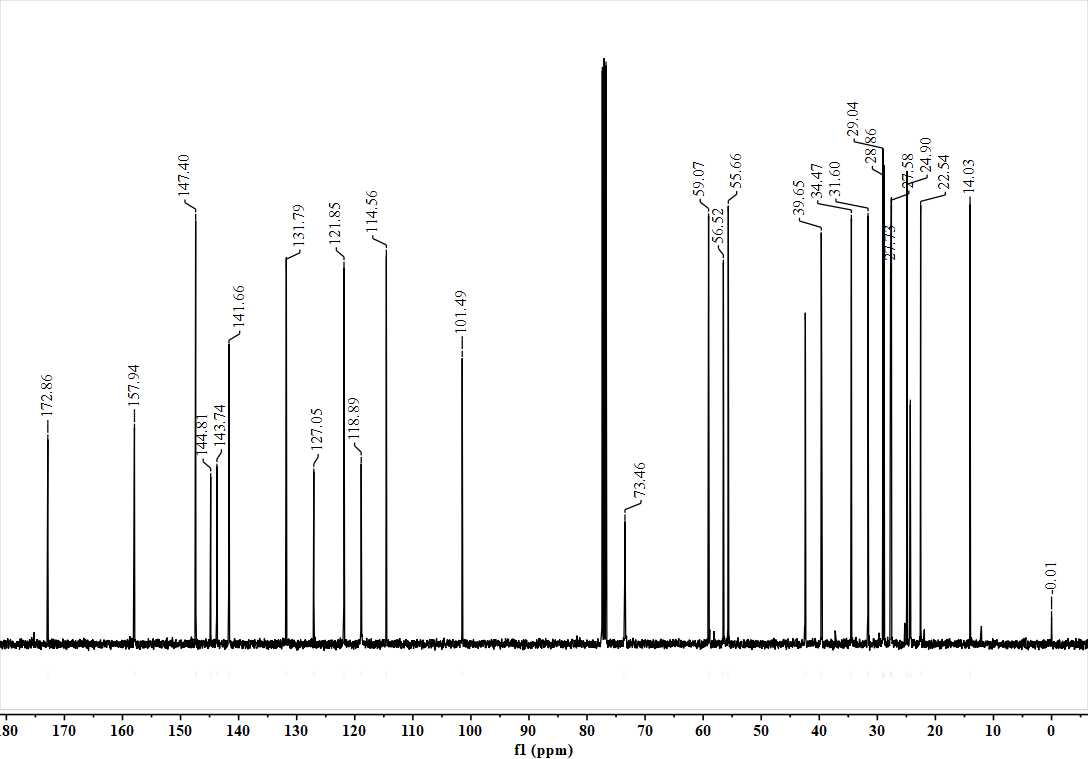
*

**Figure S10.** ^13^C-NMR of (*R*)-(6-methoxyquinolin-4-yl) (1*S*,2*S*,4*S*,5*R*)-5-vinylquinuclidin-2-yl) methyl octanoate (**7**).

*
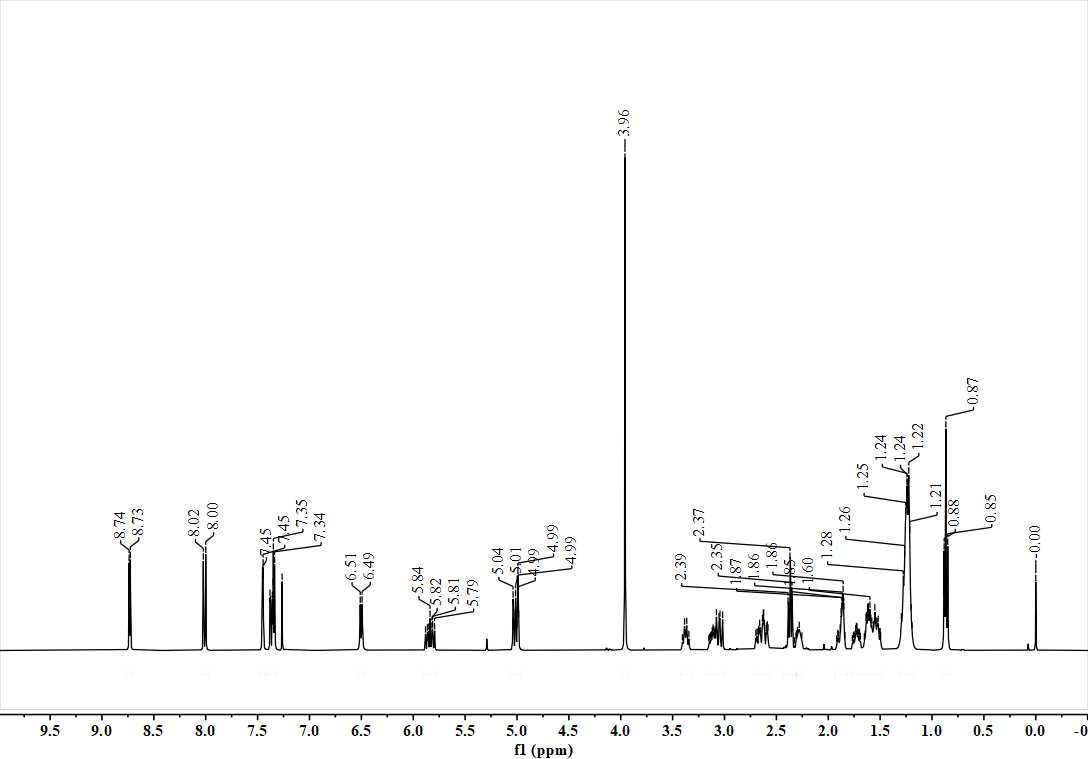
*

**Figure S11.** ^1^H-NMR of (*R*)-(6-methoxyquinolin-4-yl) (1*S*,2*S*,4*S*,5*R*)-5-vinylquinuclidin-2-yl) methyl nonanoate (**8**).

*
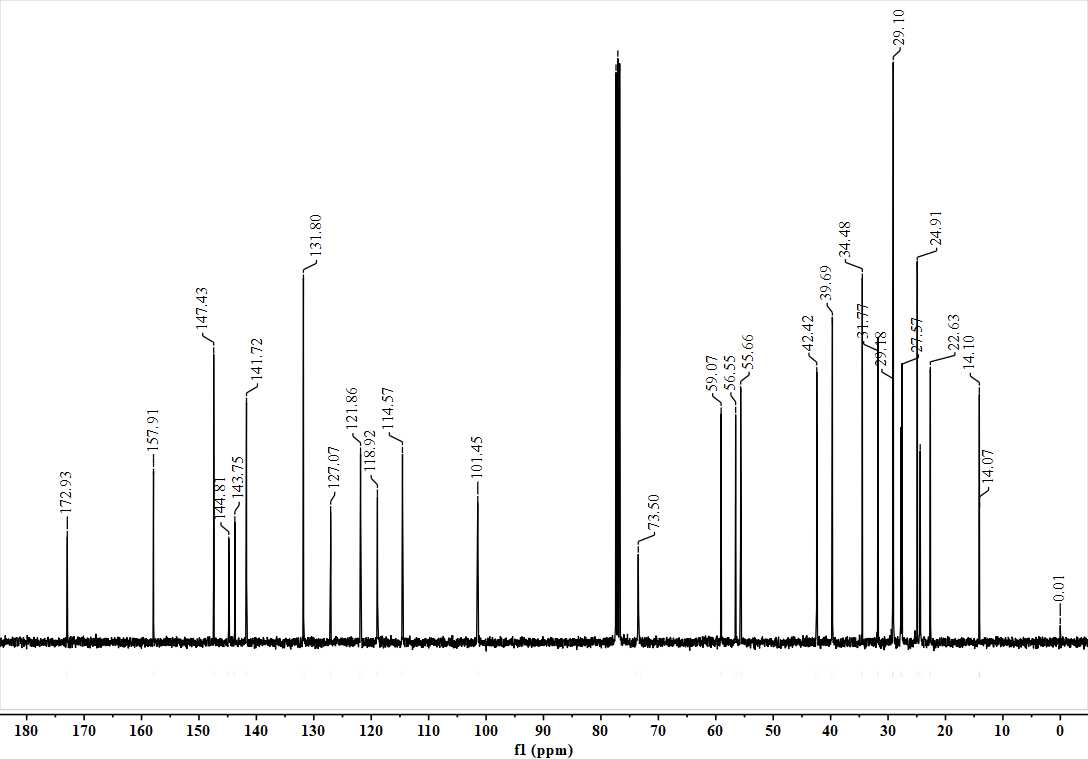
*

**Figure S12.** ^13^C-NMR of (*R*)-(6-methoxyquinolin-4-yl) (1*S*,2*S*,4*S*,5*R*)-5-vinylquinuclidin-2-yl) methyl nonanoate (**8**).

*
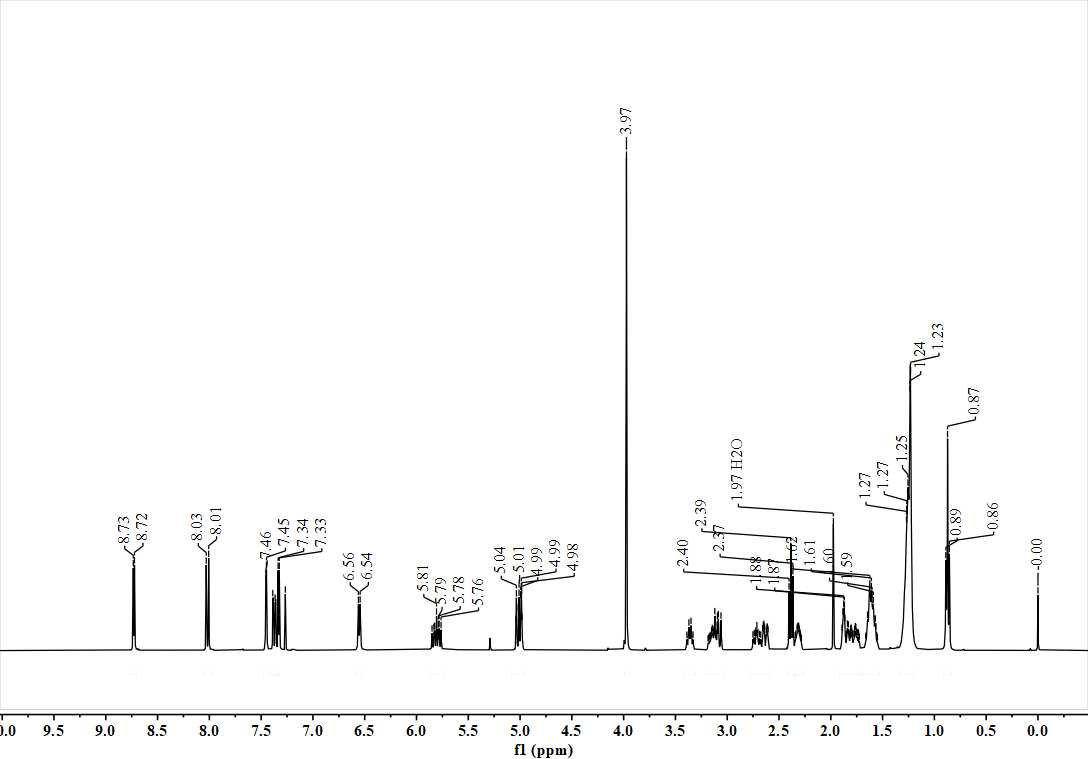
*

**Figure S13.** ^1^H-NMR of (*R*)-(6-methoxyquinolin-4-yl) (1*S*,2*S*,4*S*,5*R*)-5-vinylquinuclidin-2-yl) methyl decanoate (**9**).

*
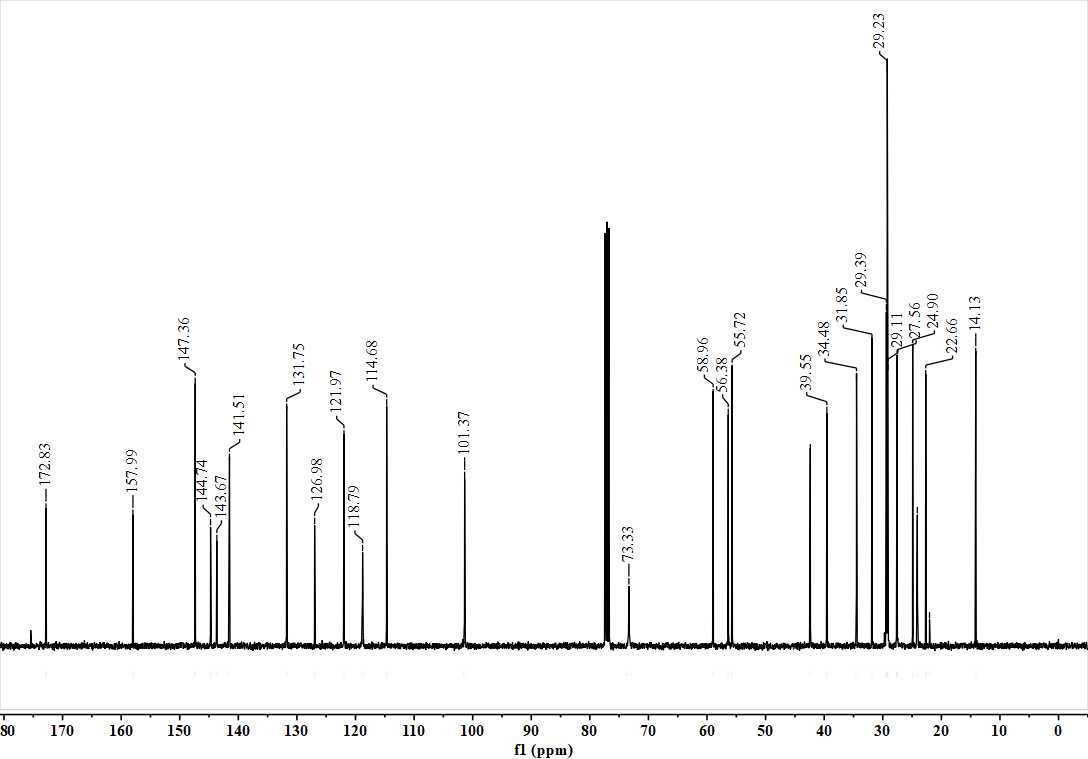
*

**Figure S14.** ^13^C-NMR of (*R*)-(6-methoxyquinolin-4-yl) (1*S*,2*S*,4*S*,5*R*)-5-vinylquinuclidin-2-yl) methyl decanoate (**9**).

*
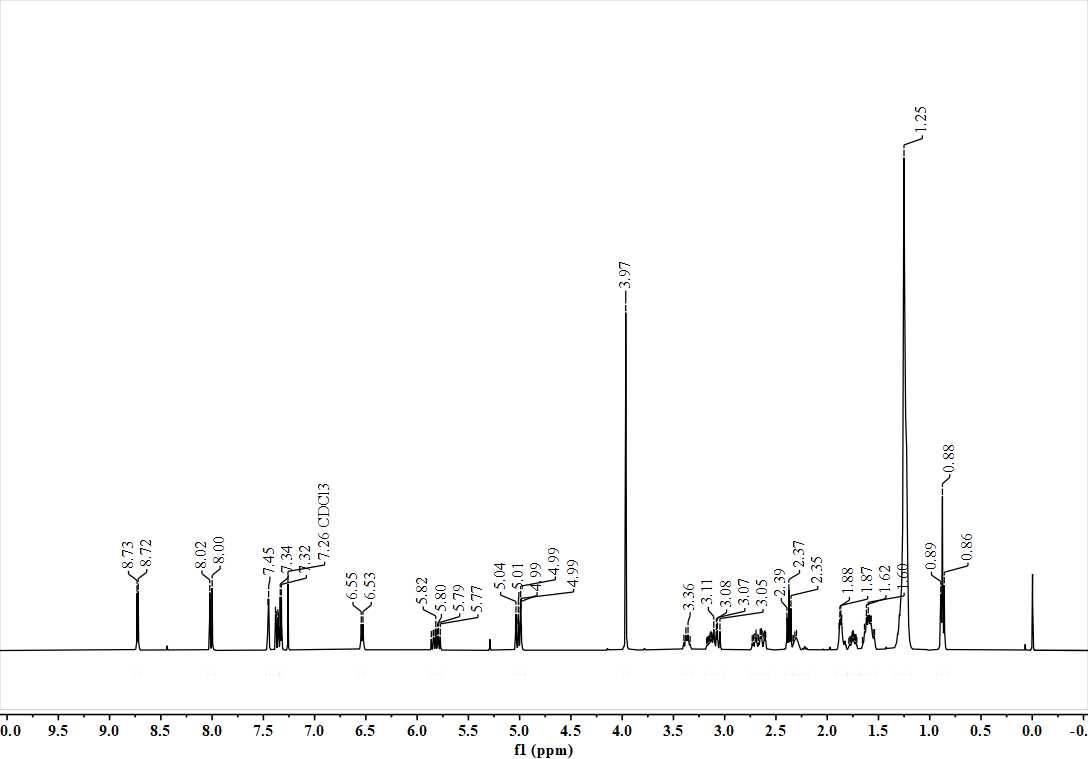
*

**Figure S15.** ^1^H-NMR of (*R*)-(6-methoxyquinolin-4-yl) (1*S*,2*S*,4*S*,5*R*)-5-vinylquinuclidin-2-yl) methyl palmitate (**10**).

*
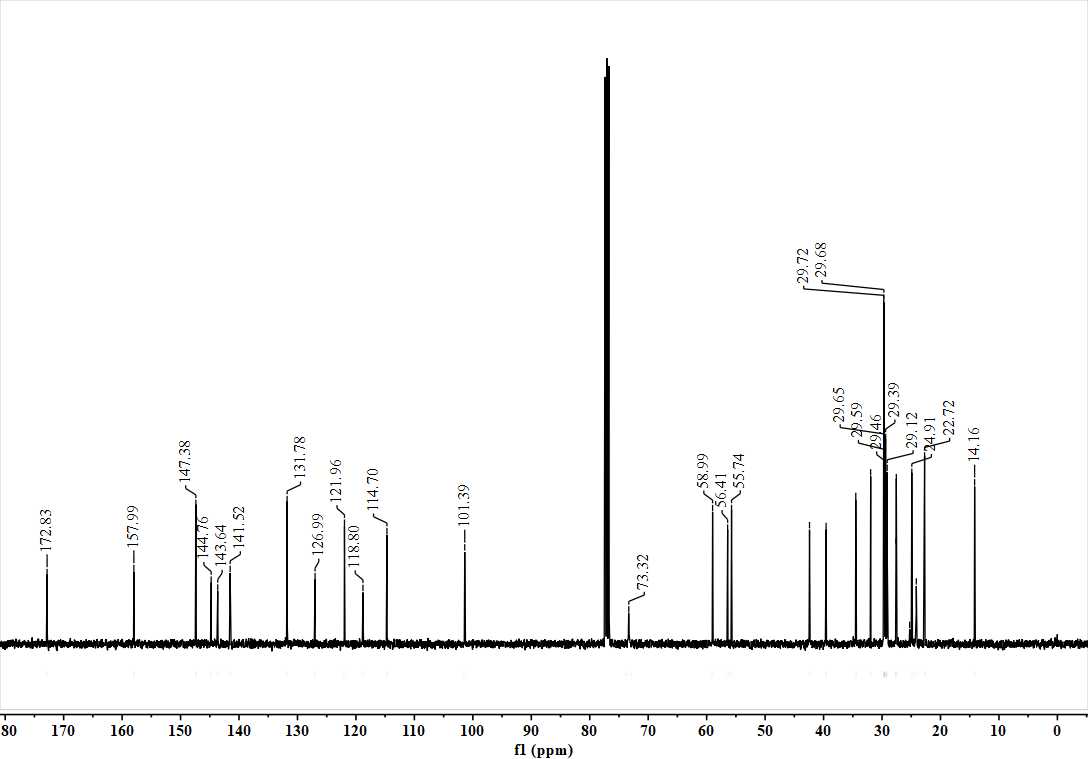
*

**Figure S16.** ^13^C-NMR of (*R*)-(6-methoxyquinolin-4-yl) (1*S*,2*S*,4*S*,5*R*)-5-vinylquinuclidin-2-yl) methyl palmitate (**10**).

*
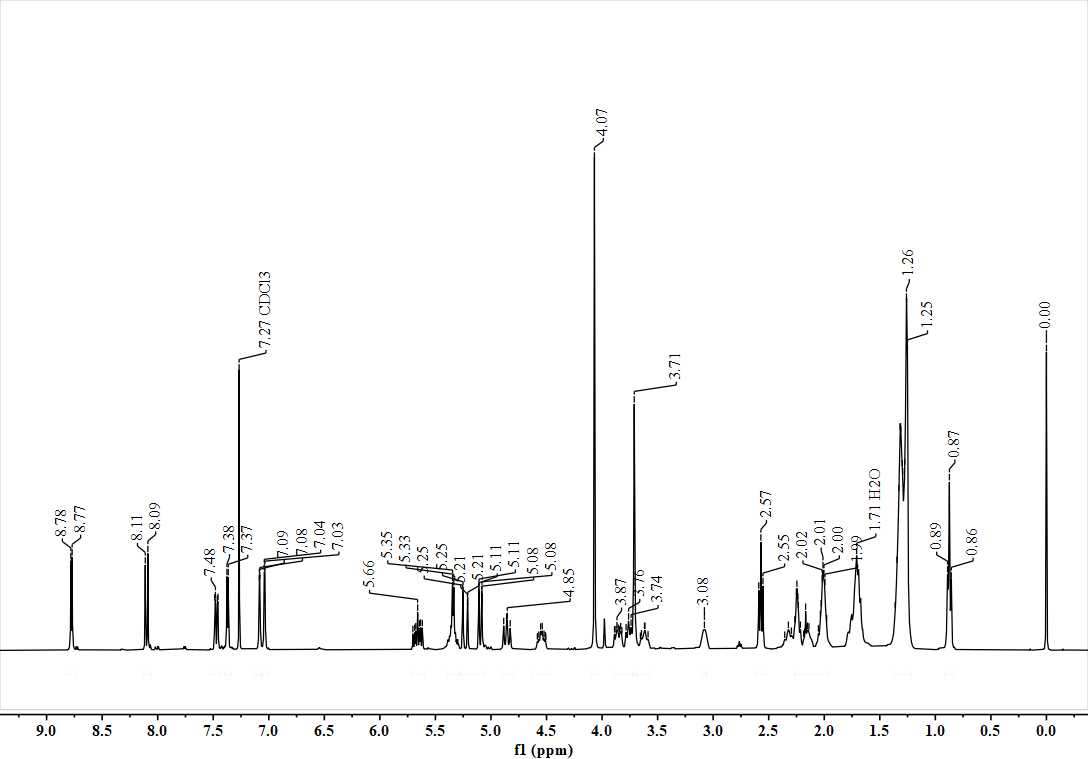
*

**Figure S17.** ^1^H-NMR of (*R*)-(6-methoxyquinolin-4-yl) (1*S*,2*S*,4*S*,5*R*)-5-vinylquinuclidin-2-yl) methyl (*E*)-octadec-9-enoate (**11**).

*
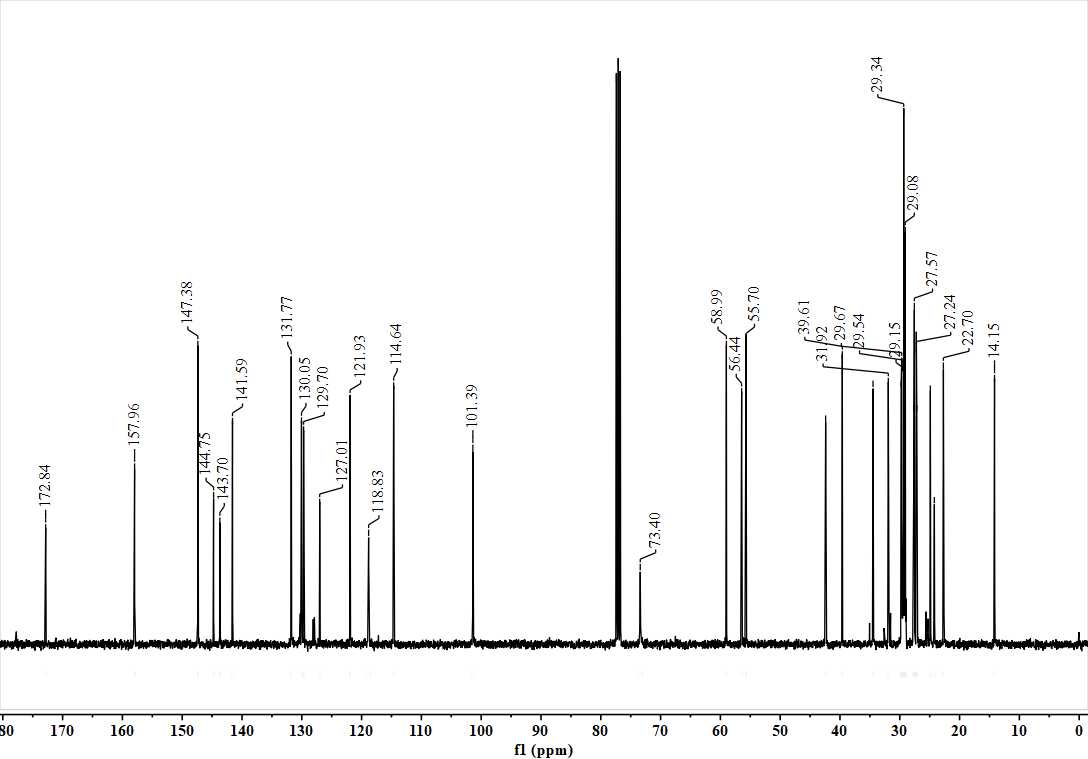
*

**Figure S18.** ^13^C-NMR of (*R*)-(6-methoxyquinolin-4-yl) (1*S*,2*S*,4*S*,5*R*)-5-vinylquinuclidin-2-yl) methyl (*E*)-octadec-9-enoate (**11**).

*
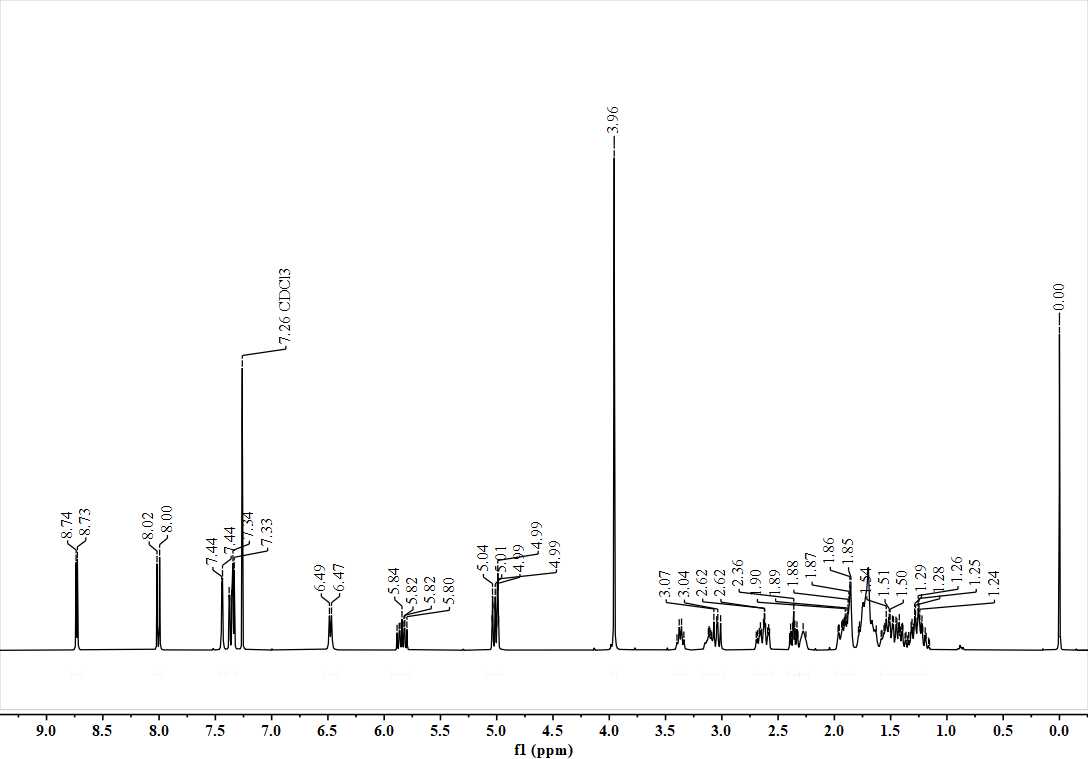
*

**Figure S19.** ^1^H-NMR of (*R*)-(6-methoxyquinolin-4-yl) (1*S*,2*S*,4*S*,5*R*)-5-vinylquinuclidin-2-yl) methyl cyclohexanecarboxylate (**12**).

*
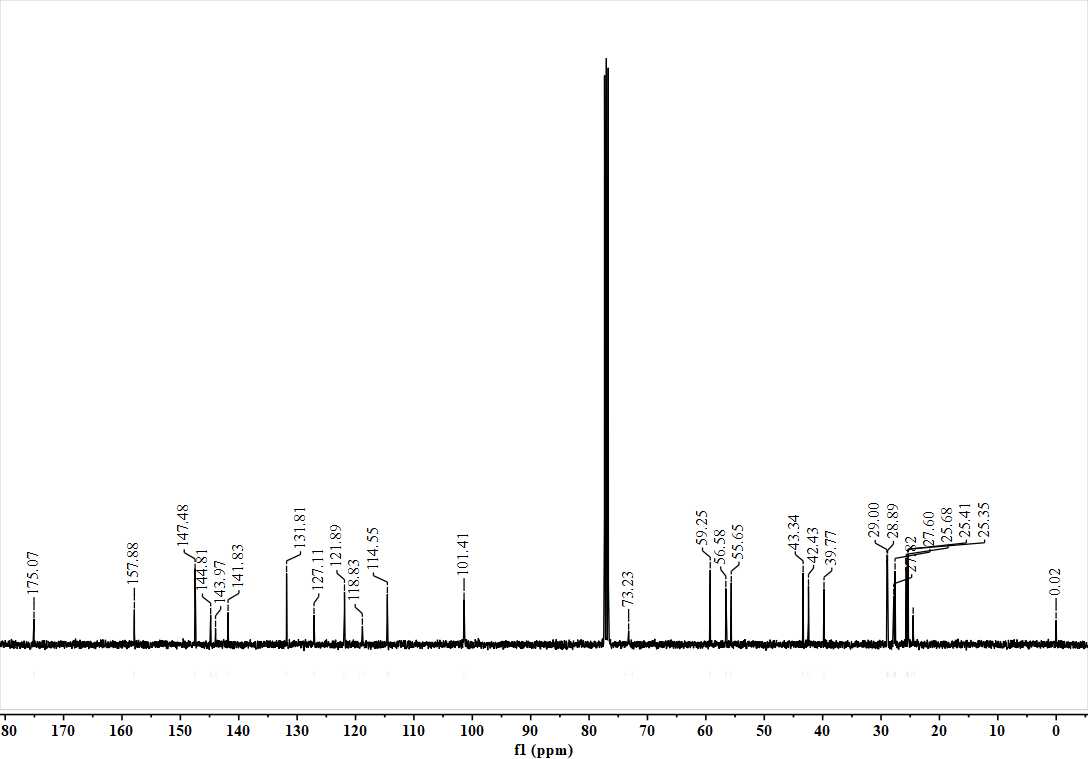
*

**Figure S20.** ^13^C-NMR of (*R*)-(6-methoxyquinolin-4-yl) (1*S*,2*S*,4*S*,5*R*)-5-vinylquinuclidin-2-yl) methyl cyclohexanecarboxylate (**12**).

*
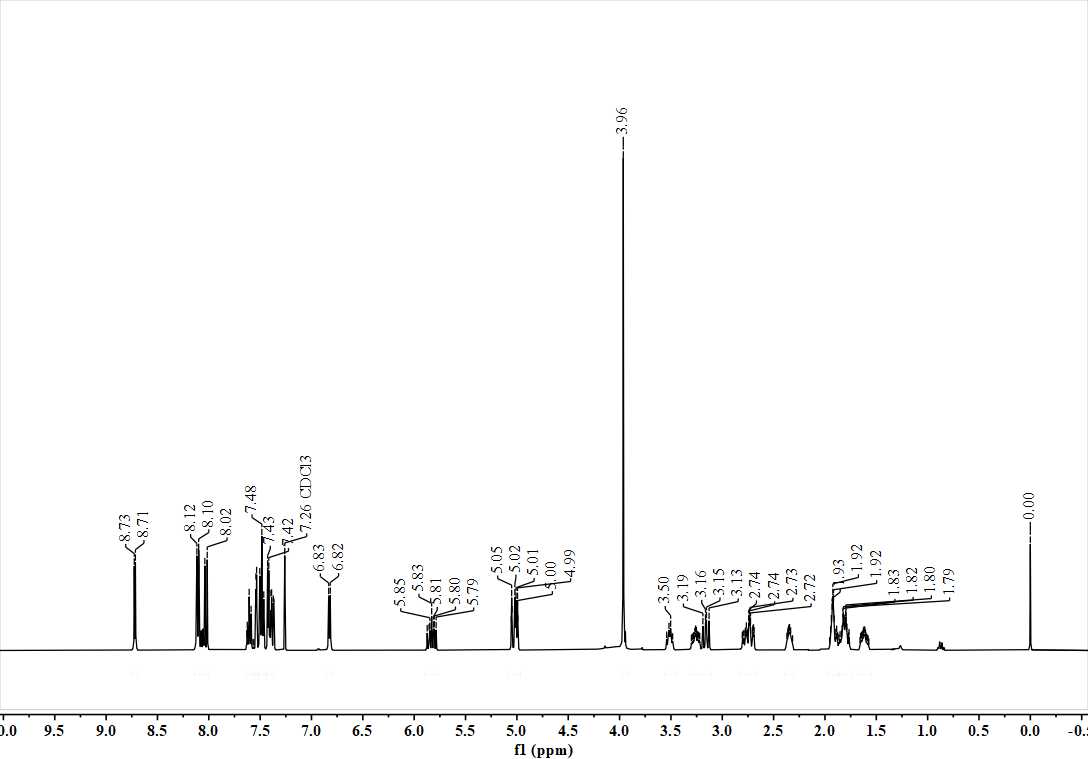
*

**Figure S21.** ^1^H-NMR of (*R*)-(6-methoxyquinolin-4-yl) (1*S*,2*S*,4*S*,5*R*)-5-vinylquinuclidin-2-yl) methyl benzoate (**13**).

*
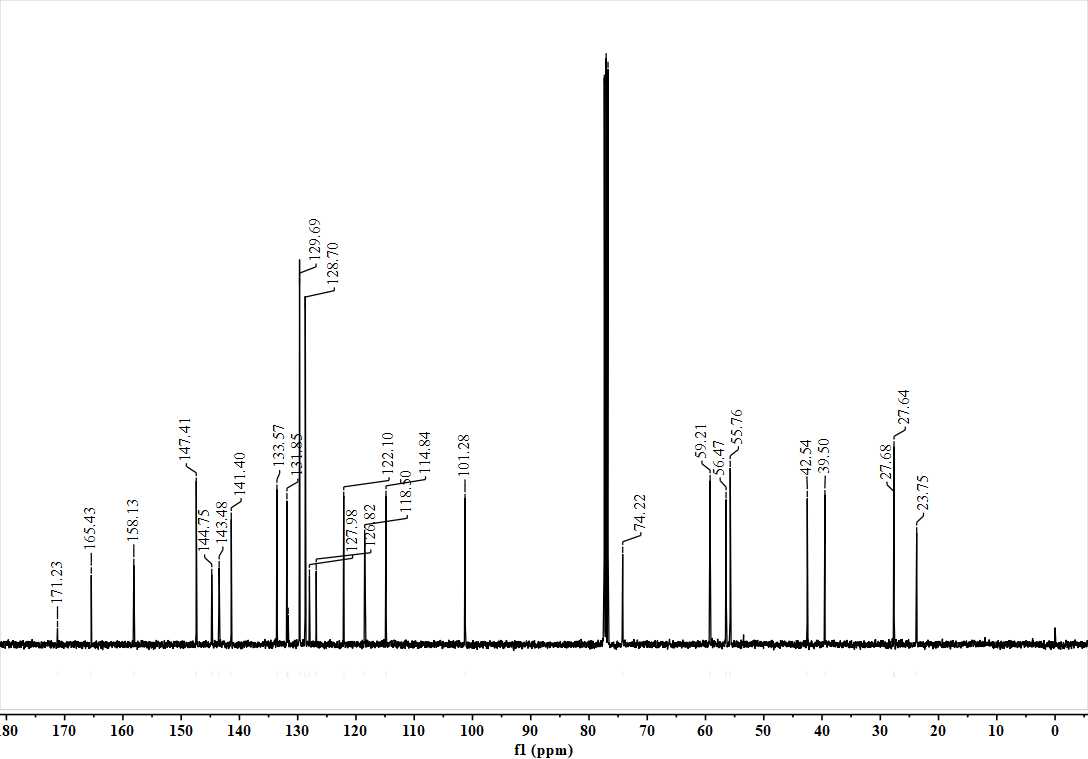
*

**Figure S22.** ^13^C-NMR of (*R*)-(6-methoxyquinolin-4-yl) (1*S*,2*S*,4*S*,5*R*)-5-vinylquinuclidin-2-yl) methyl benzoate (**13**).

*
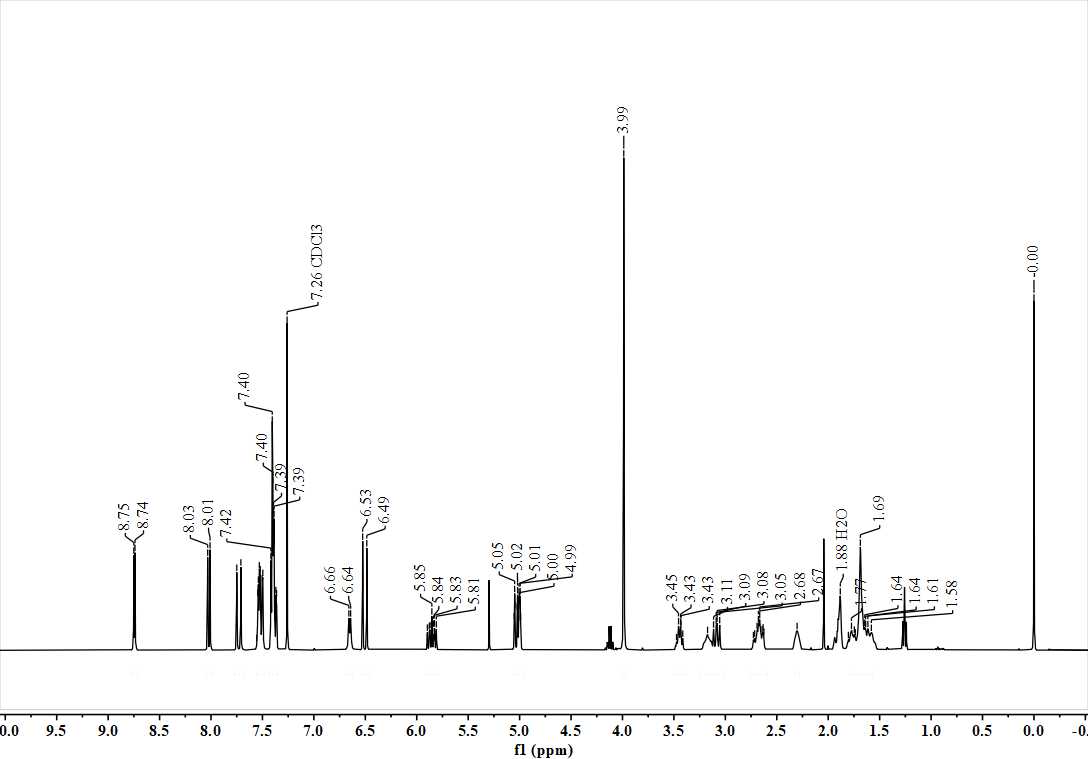
*

**Figure S23.** ^1^H-NMR of (*R*)-(6-methoxyquinolin-4-yl) (1*S*,2*S*,4*S*,5*R*)-5-vinylquinuclidin-2-yl) methyl cinnamate (**14**).

*
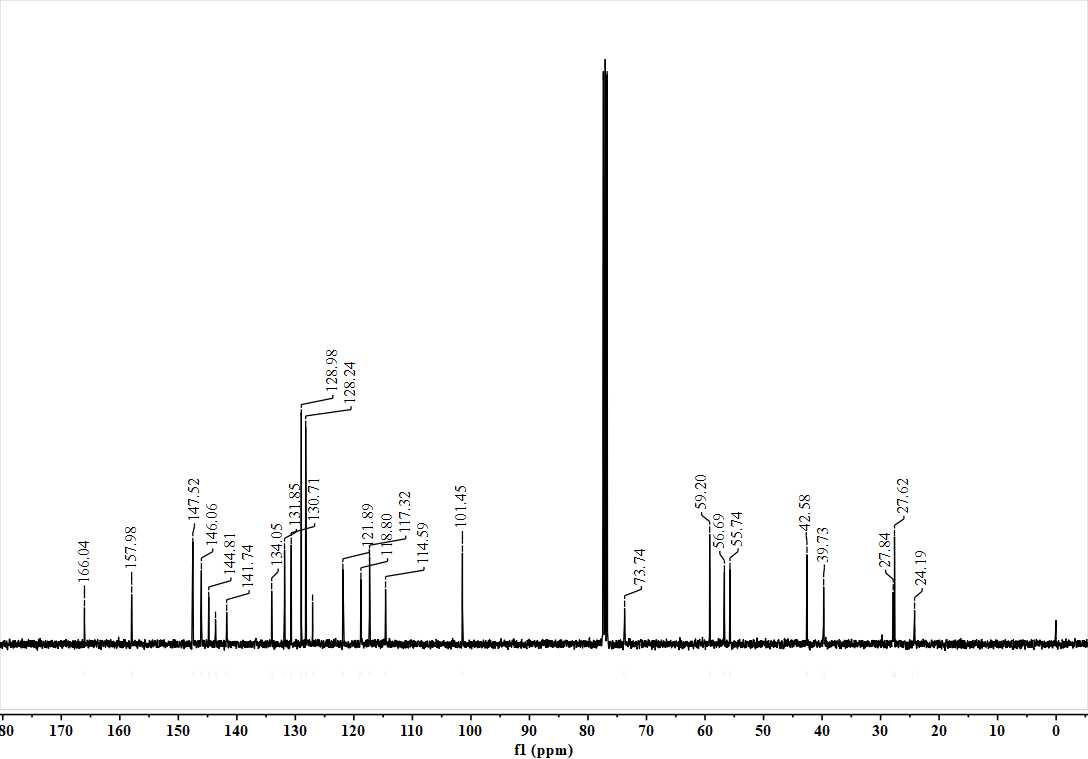
***Figure S24.** ^13^C-NMR of (*R*)-(6-methoxyquinolin-4-yl) (1*S*,2*S*,4*S*,5*R*)-5-vinylquinuclidin-2-yl) methyl cinnamate (**14**).


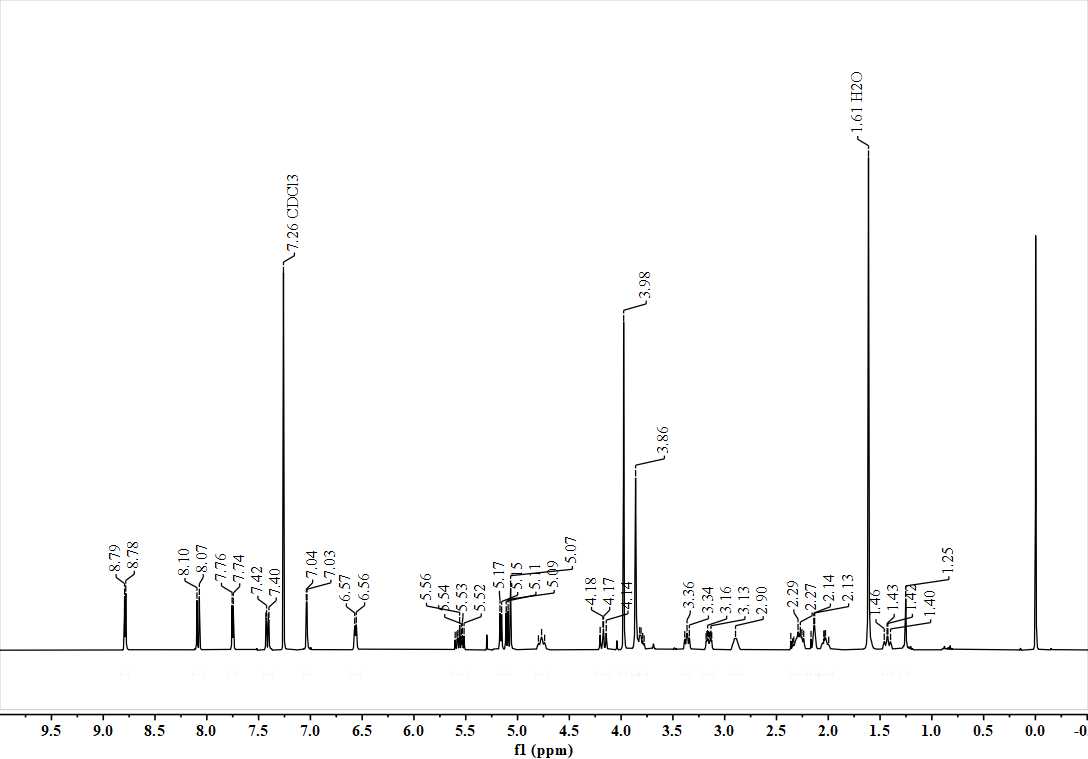


**Figure S25.** ^1^H-NMR of (1*S*,2*S*,4*S*,5*R*)-2-(*R*)-hydroxy(6-methoxyquinolin-4-yl) methyl)-1-methyl-5-vinylquinuclidin-1-ium iodide (**15**).


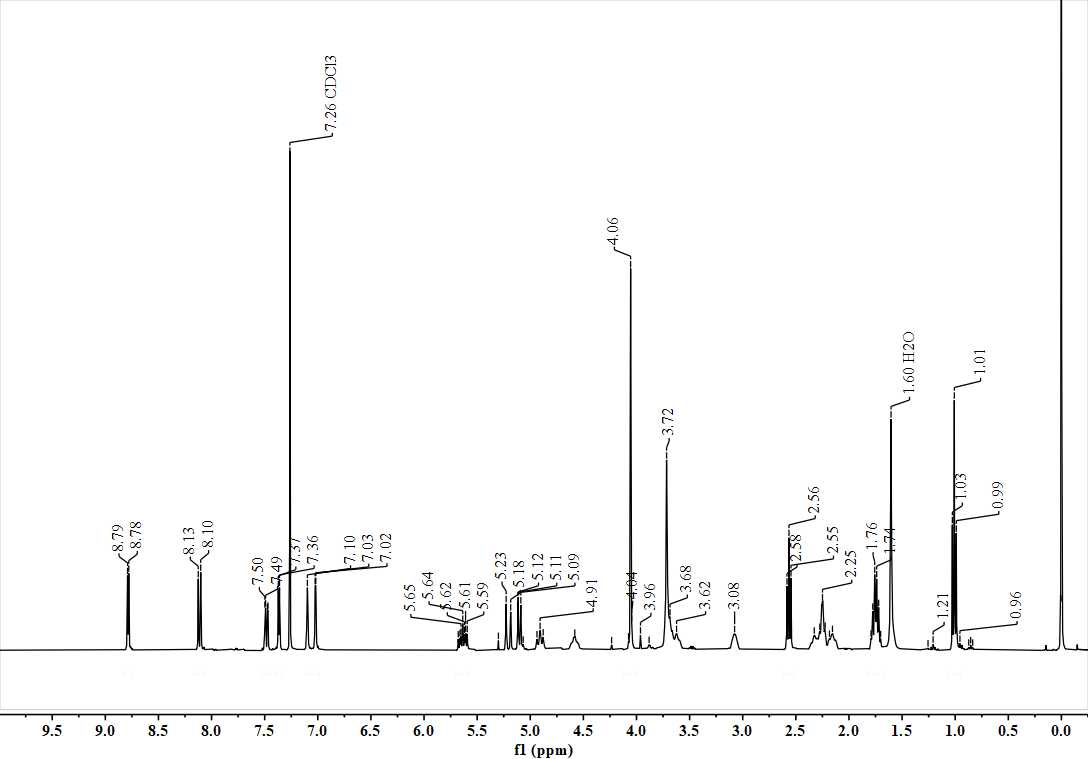


**Figure S26.** ^1^H-NMR of (1*S*,2*S*,4*S*,5*R*)-2-(*R*)-(butyryloxy)(6-methoxyquinolin-4-yl)methyl)-1-methyl-5-vinylquinuclidin-1-ium iodide (**16**).


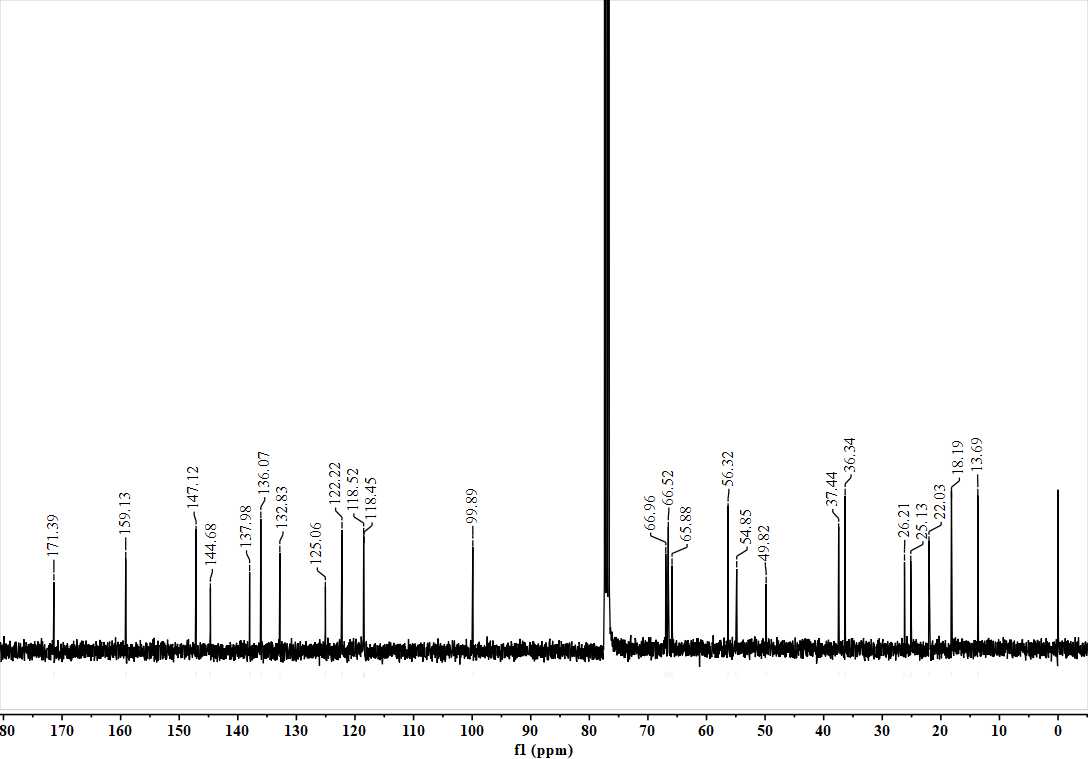


**Figure S27.** ^13^C-NMR of (1*S*,2*S*,4*S*,5*R*)-2-(*R*)-(butyryloxy)(6-methoxyquinolin-4-yl)methyl)-1-methyl-5-vinylquinuclidin-1-ium iodide (**16**).


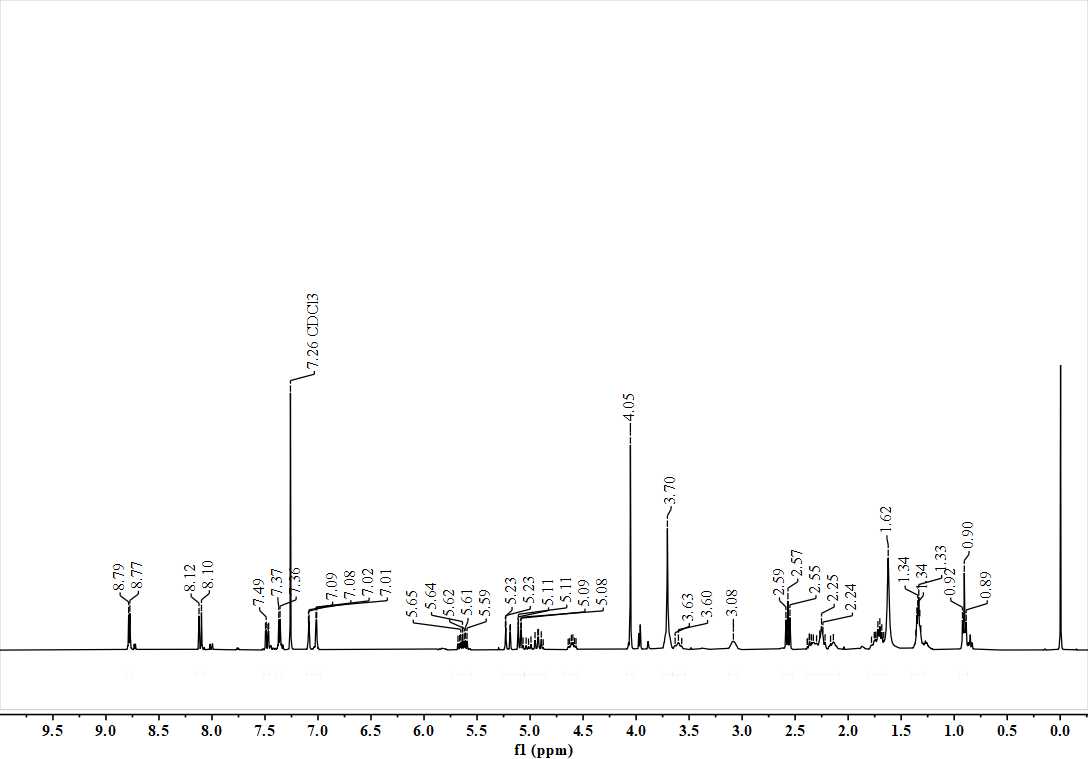


**Figure S28.** ^1^H-NMR of (1*S*,2*S*,4*S*,5*R*)-2-(*R*) -(hexanoyloxy) (6-methoxyquinolin-4-yl) methyl)-1-methyl-5-vinylquinuclidin-1-ium iodide (**17**).


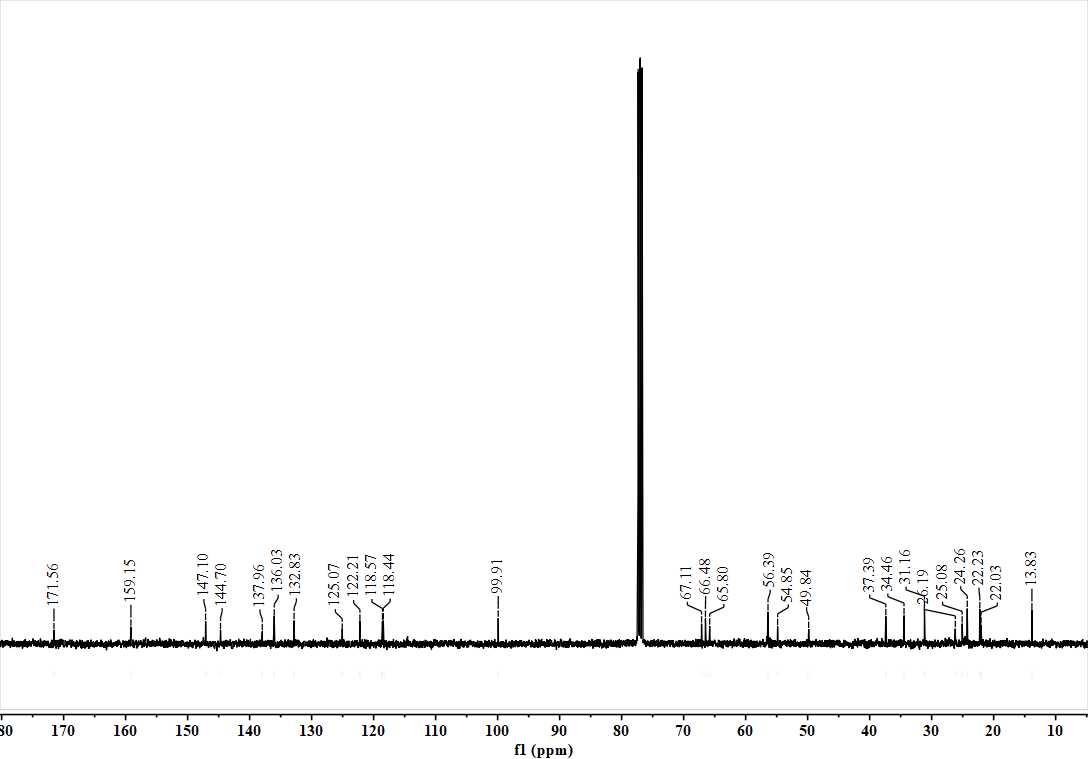


**Figure S29.** ^13^C-NMR of (1*S*,2*S*,4*S*,5*R*)-2-(*R*) -(hexanoyloxy) (6-methoxyquinolin-4-yl) methyl)-1-methyl-5-vinylquinuclidin-1-ium iodide (**17**).


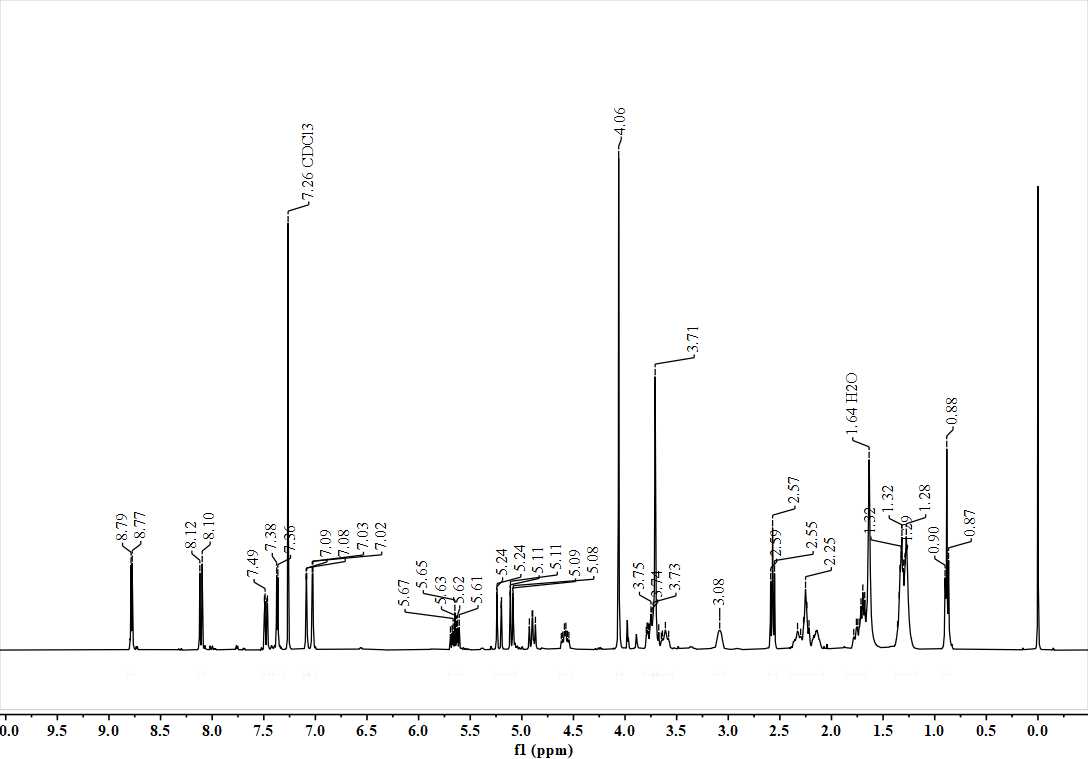


**Figure S30.** ^1^H-NMR of (1*S*,2*S*,4*S*,5*R*)-2-(*R*)-(6-methoxyquinolin-4-yl)(octanoyloxy)methyl)-1-methyl-5 vinylquinuclidin-1-ium iodide (**18**).


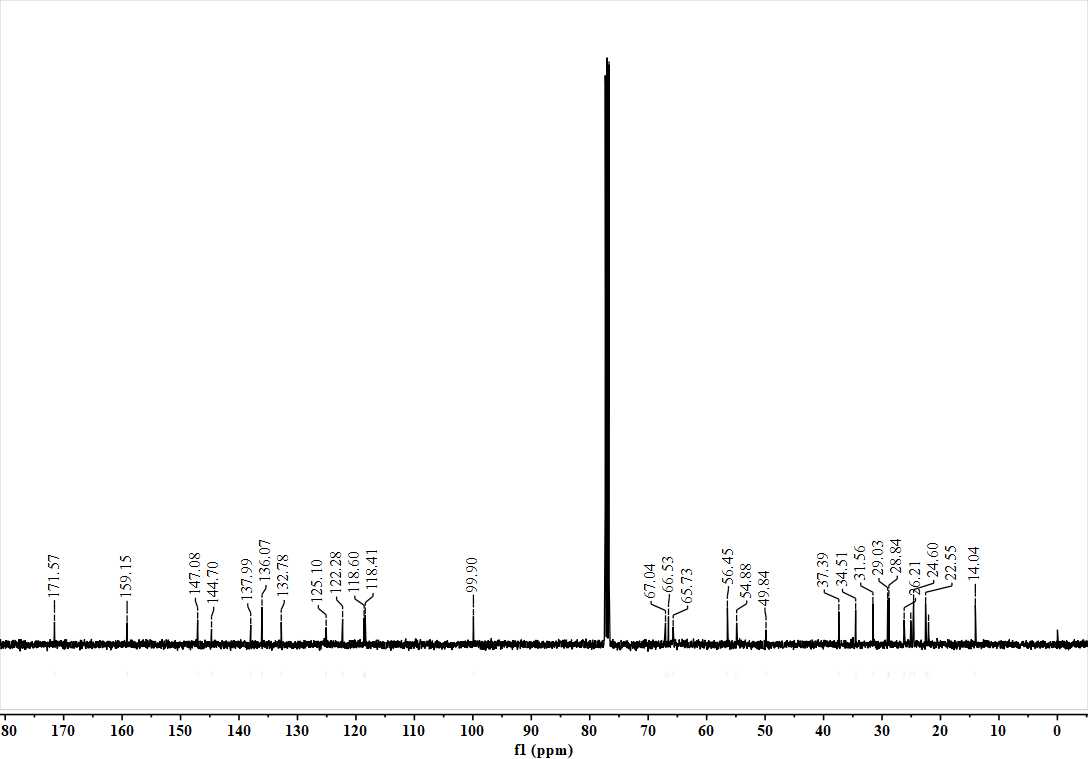


**Figure S31.** ^13^C-NMR of (1*S*,2*S*,4*S*,5*R*)-2-(*R*)-(6-methoxyquinolin-4-yl)(octanoyloxy)methyl)-1-methyl-5 vinylquinuclidin-1-ium iodide (**18**).


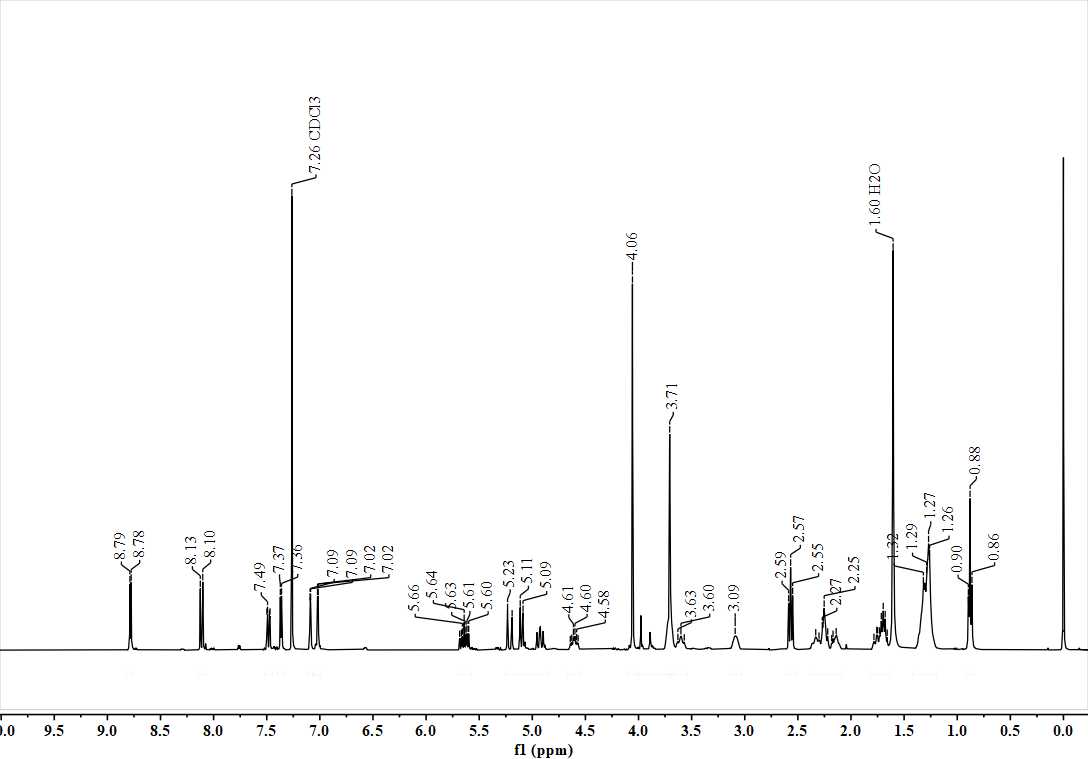


**Figure S32.** ^1^H-NMR of (1*S*,2*S*,4*S*,5*R*)-2-(*R*)-(6-methoxyquinolin-4-yl)(nonanoyloxy)methyl)-1-methyl-5-vinylquinuclidin-1-ium iodide (**19**).


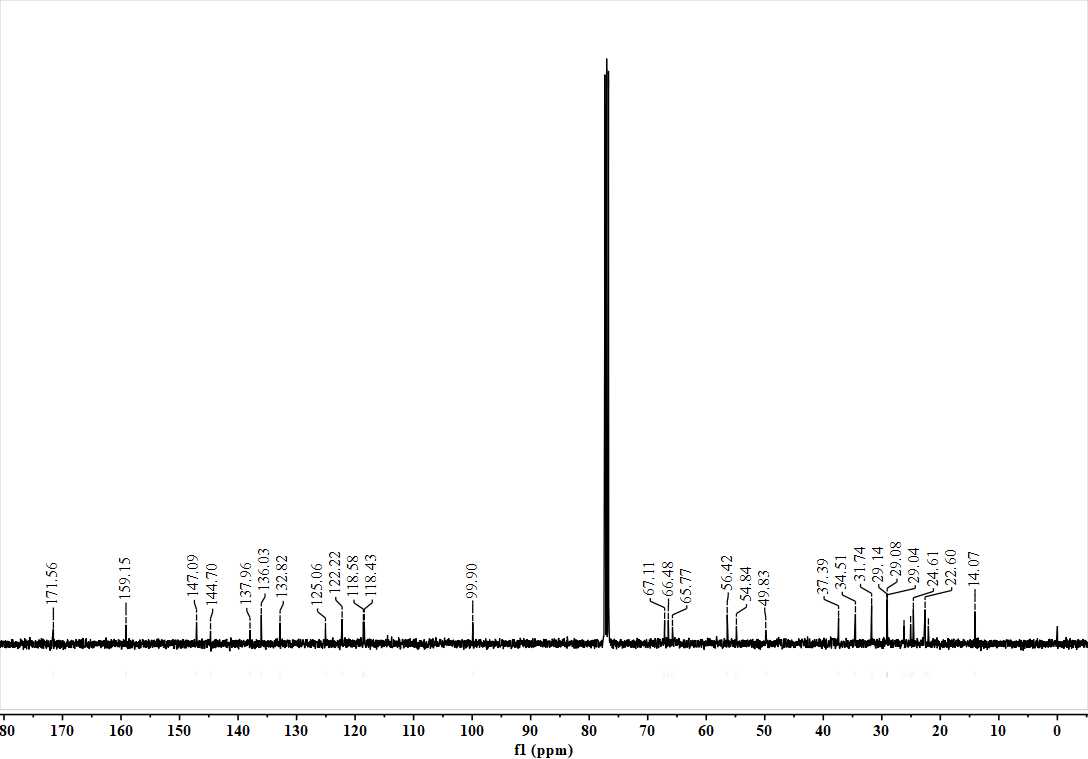


**Figure S33.** ^13^C-NMR of (1*S*,2*S*,4*S*,5*R*)-2-(*R*)-(6-methoxyquinolin-4-yl)(nonanoyloxy)methyl)-1-methyl-5-vinylquinuclidin-1-ium iodide (**19**).


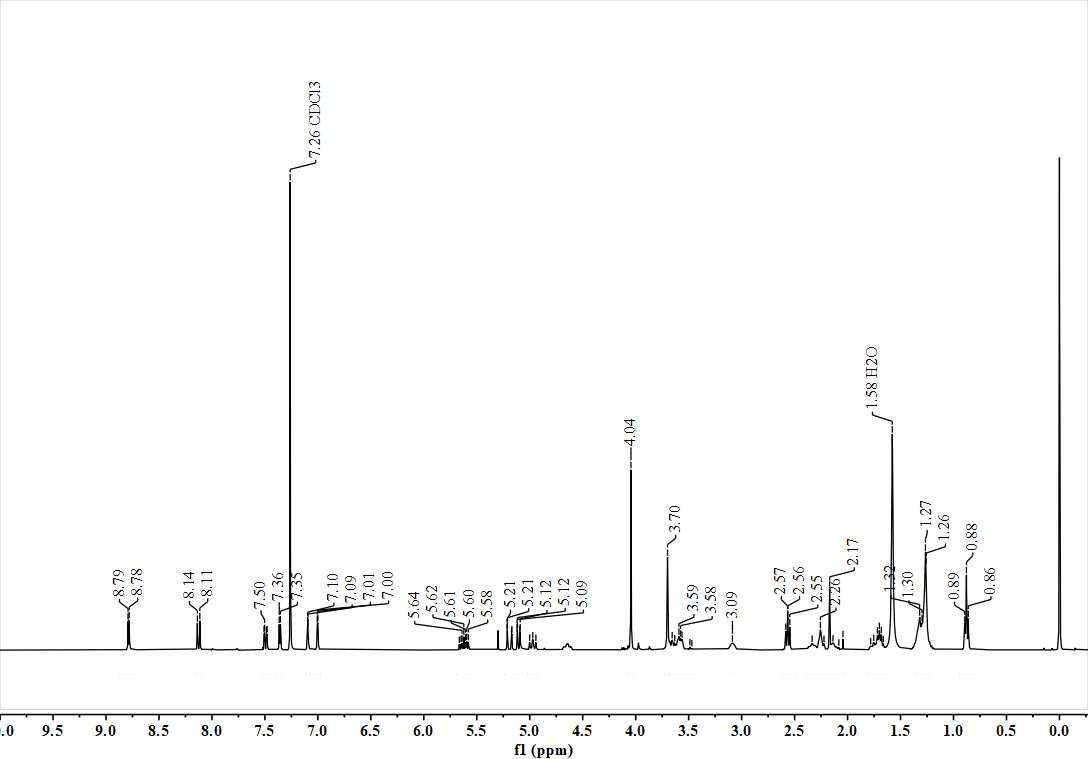


**Figure S34.** ^1^H-NMR of ((1*S*,2*S*,4*S*,5*R*)-2-(*R*)-(decanoyloxy)(6-methoxyquinolin-4-yl)methyl)-1-methyl-5-vinylquinuclidin-1-ium iodide (**20**).


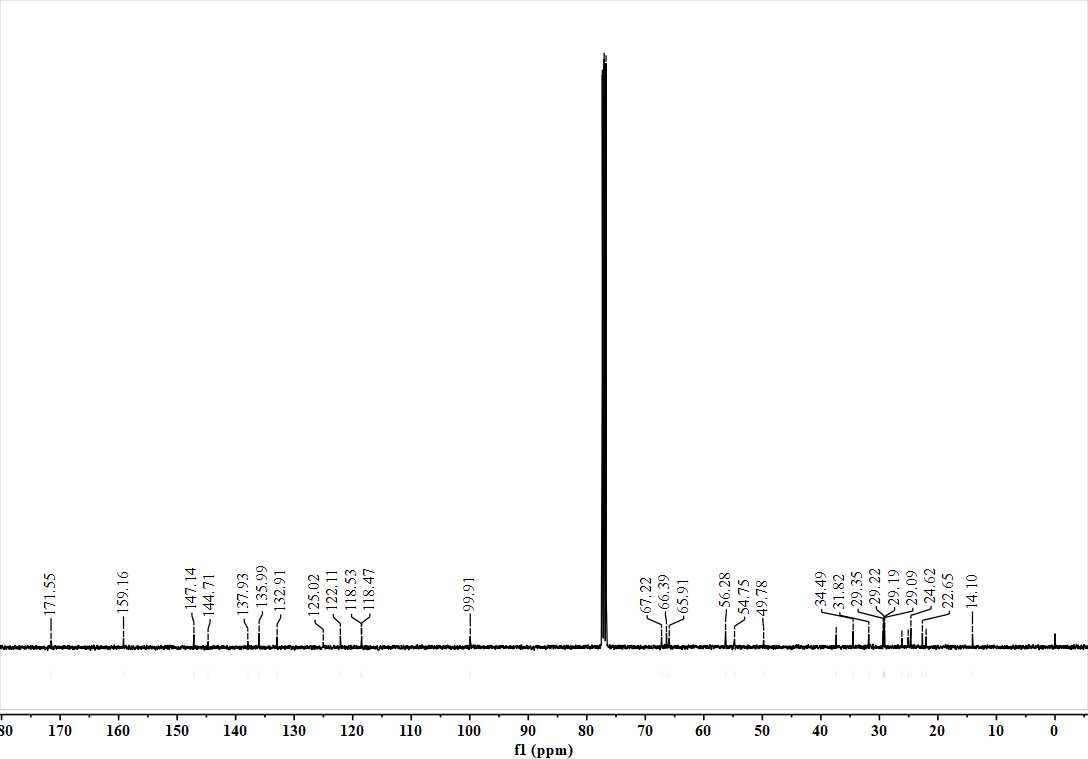


**Figure S35.** ^13^C-NMR of ((1*S*,2*S*,4*S*,5*R*)-2-(*R*)-(decanoyloxy)(6-methoxyquinolin-4-yl)methyl)-1-methyl-5-vinylquinuclidin-1-ium iodide (**20**).


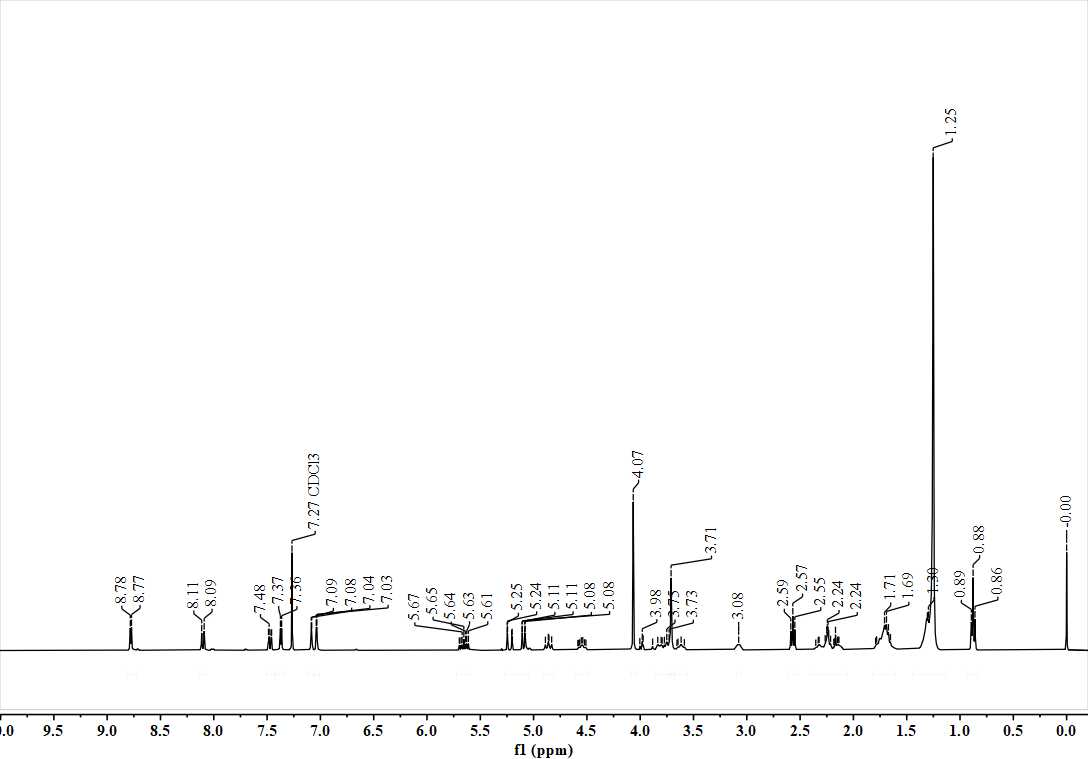


**Figure S36.** ^1^H-NMR of (1*S*,*2S*,4*S*,5*R*)-2-((*R*)-(6-methoxyquinolin-4-yl)(palmitoyloxy)methyl)-1-methyl-5-vinylquinuclidin-1-ium iodide (**21**).


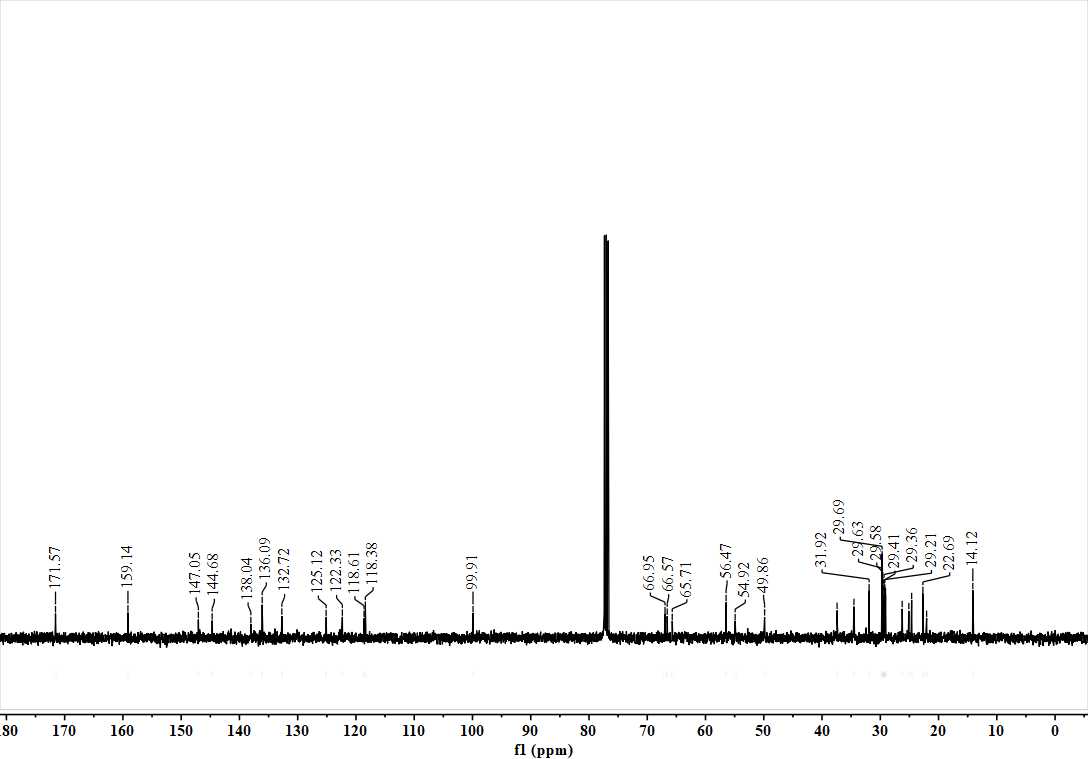


**Figure S37.** ^13^C-NMR of (1*S*,2*S*,4*S*,5*R*)-2-((*R*)-(6-methoxyquinolin-4-yl)(palmitoyloxy)methyl)-1-methyl-5-vinylquinuclidin-1-ium iodide (**21**).


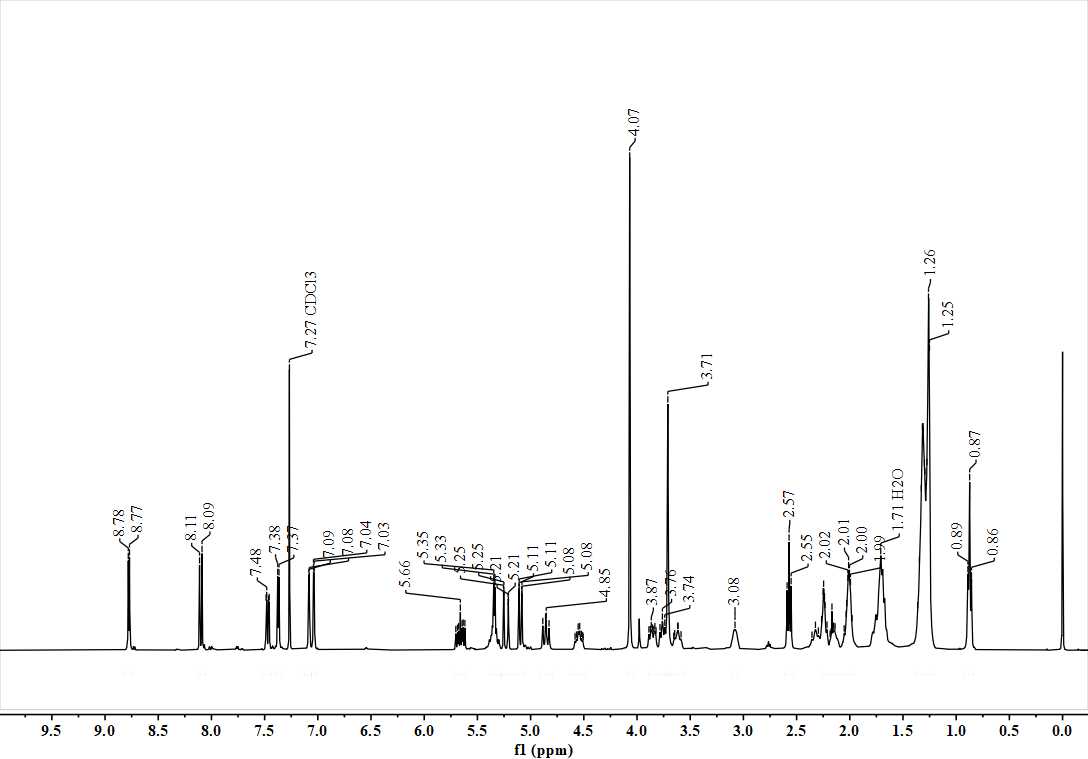


**Figure S38.** ^1^H-NMR of (1*S*,2*S*,4*S*,5*R*)-2-((*R*)-(6-methoxyquinolin-4-yl) (((E)-octadec-9-enoyl) oxy) methyl)-1-methyl-5-vinylquinuclidin-1-ium iodide (**22**).


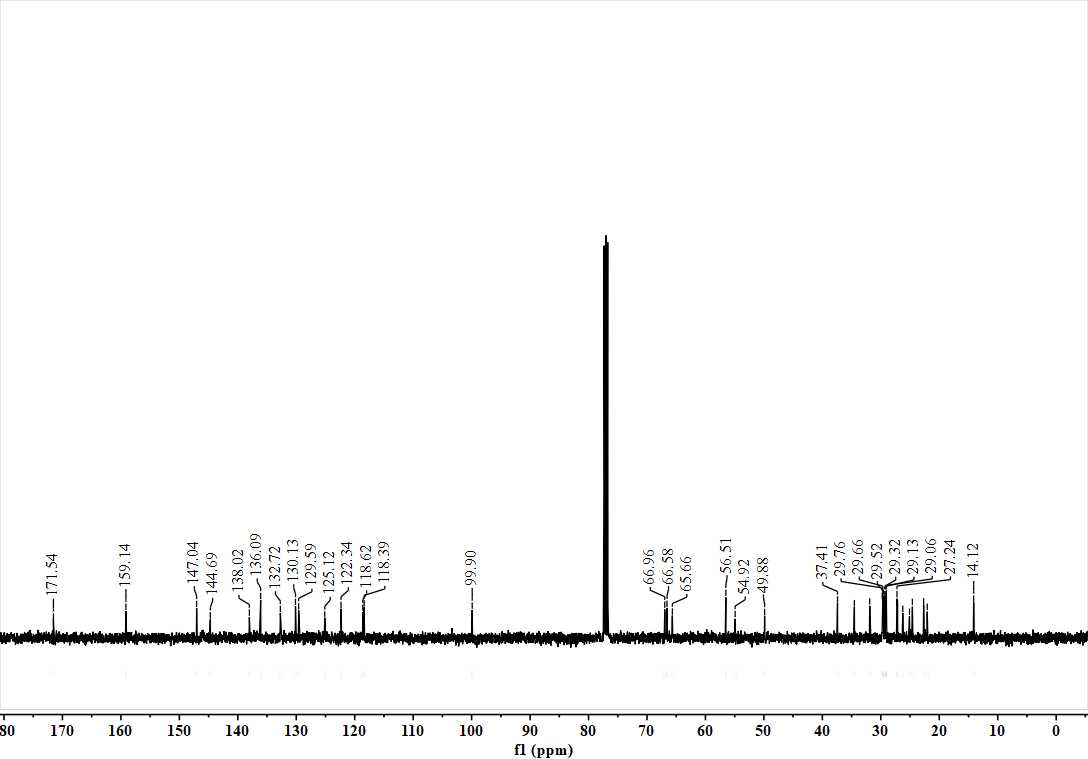


**Figure S39.** ^13^C-NMR of (1*S*,2*S*,4*S*,5*R*)-2-((*R*)-(6-methoxyquinolin-4-yl) (((E)-octadec-9-enoyl) oxy) methyl)-1-methyl-5-vinylquinuclidin-1-ium iodide (**22**).


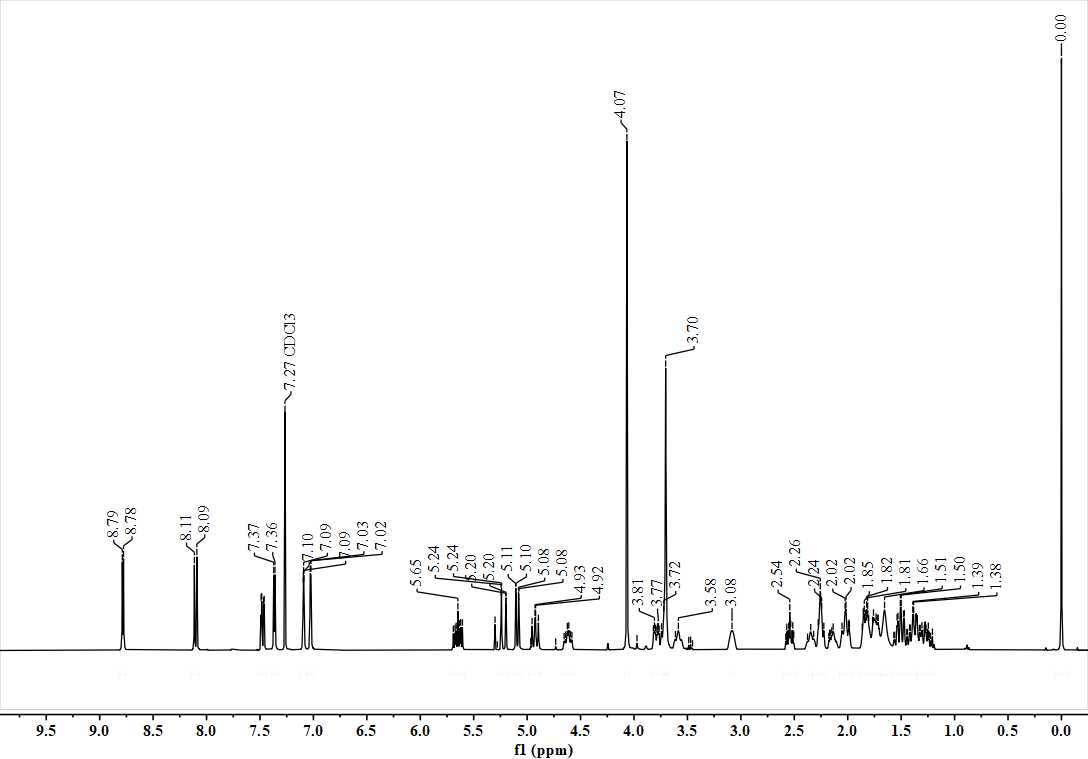


**Figure S40.** ^1^H-NMR of (1*S*,2*S*,4*S*,5*R*)-2-((*R*)-((cyclohexanecarbonyl)oxy)(6-methoxyquinolin-4-yl)methyl)-1-methyl-5-vinylquinuclidin-1-ium iodide (**23**).


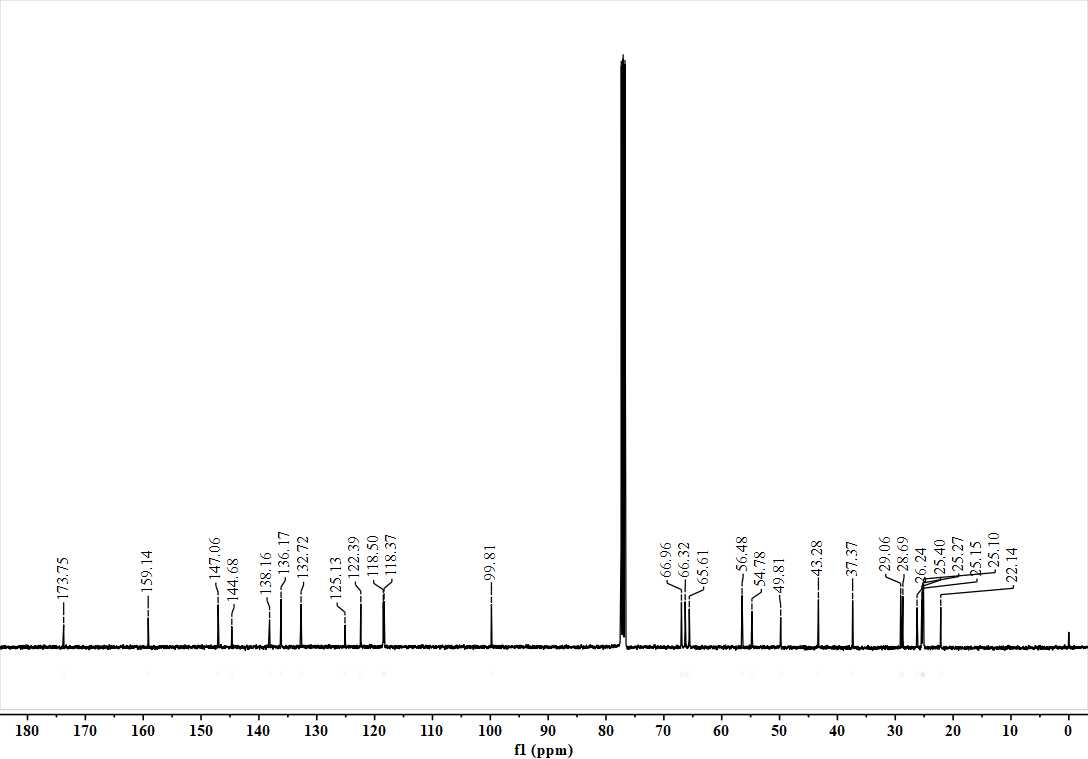


**Figure S41.** ^13^C-NMR of (1*S*,2*S*,4*S*,5*R*)-2-((*R*)-((cyclohexanecarbonyl)oxy)(6-methoxyquinolin-4-yl)methyl)-1-methyl-5-vinylquinuclidin-1-ium iodide (**23**).


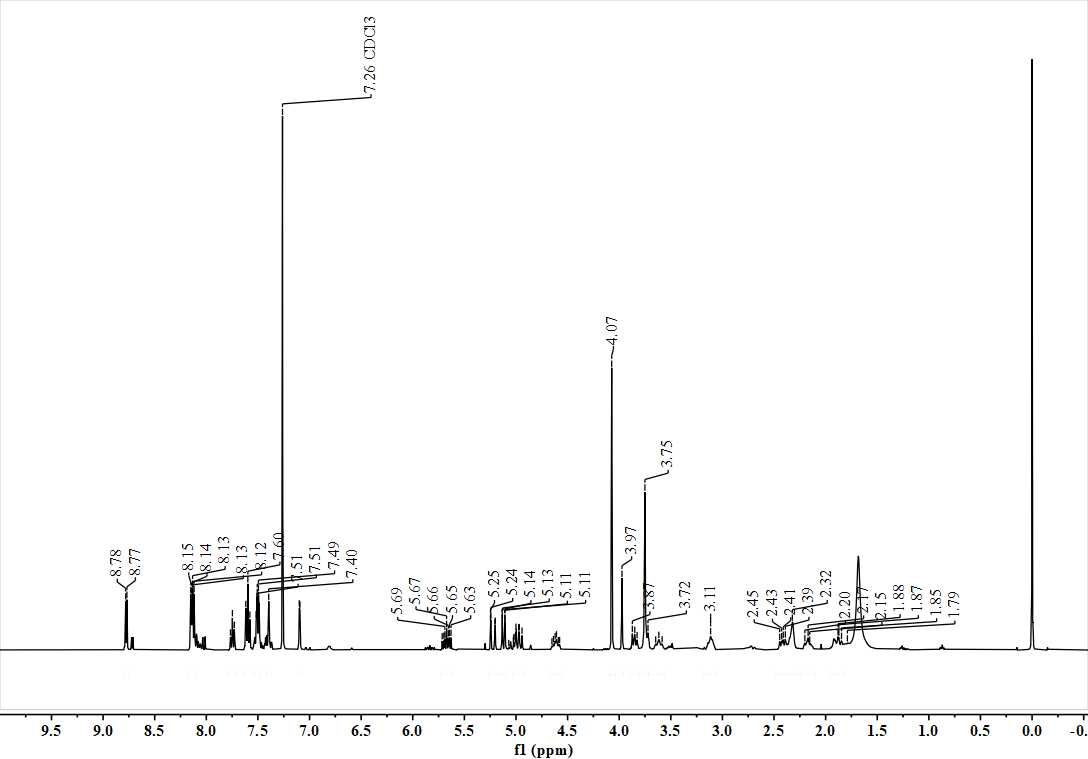


**Figure S42.** ^1^H-NMR of (1*S*,2*S*,4*S*,5*R*)-2-((*R*)-(benzoyloxy)(6-methoxyquinolin-4-yl)methyl)-1-methyl-5-vinylquinuclidin-1-ium iodide (**24**).


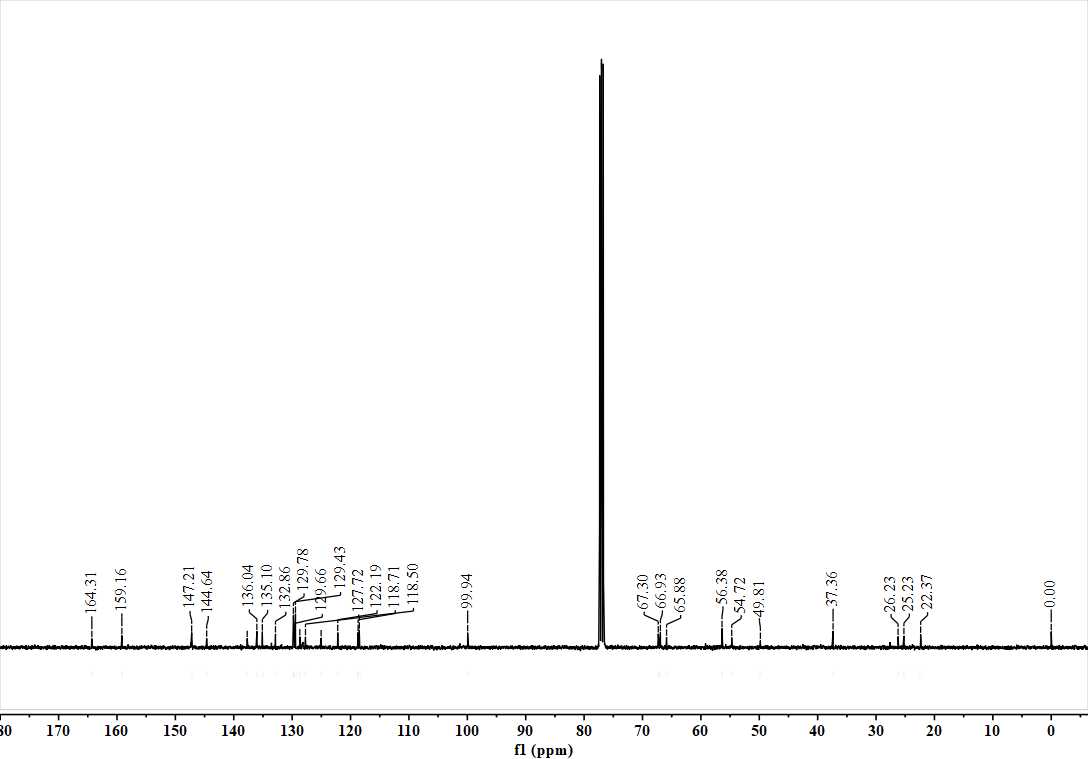


**Figure S43.** ^13^C-NMR of (1*S*,2*S*,4*S*,5*R*)-2-((*R*)-(benzoyloxy)(6-methoxyquinolin-4-yl)methyl)-1-methyl-5-vinylquinuclidin-1-ium iodide (**24**).


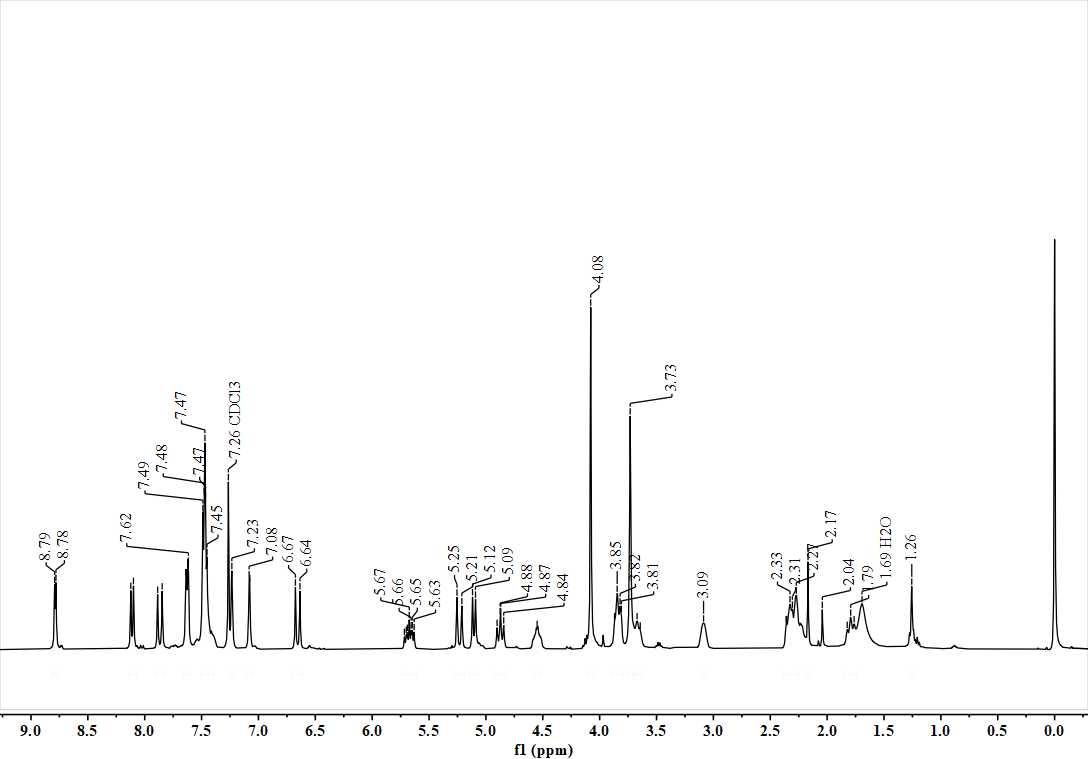


**Figure S44.** ^1^H-NMR of (1*S*,2*S*,4*S*,5*R*)-2-((*R*)-(cinnamoyloxy)(6-methoxyquinolin-4-yl)methyl)-1-methyl-5-vinylquinuclidin-1-ium iodide (**25**).


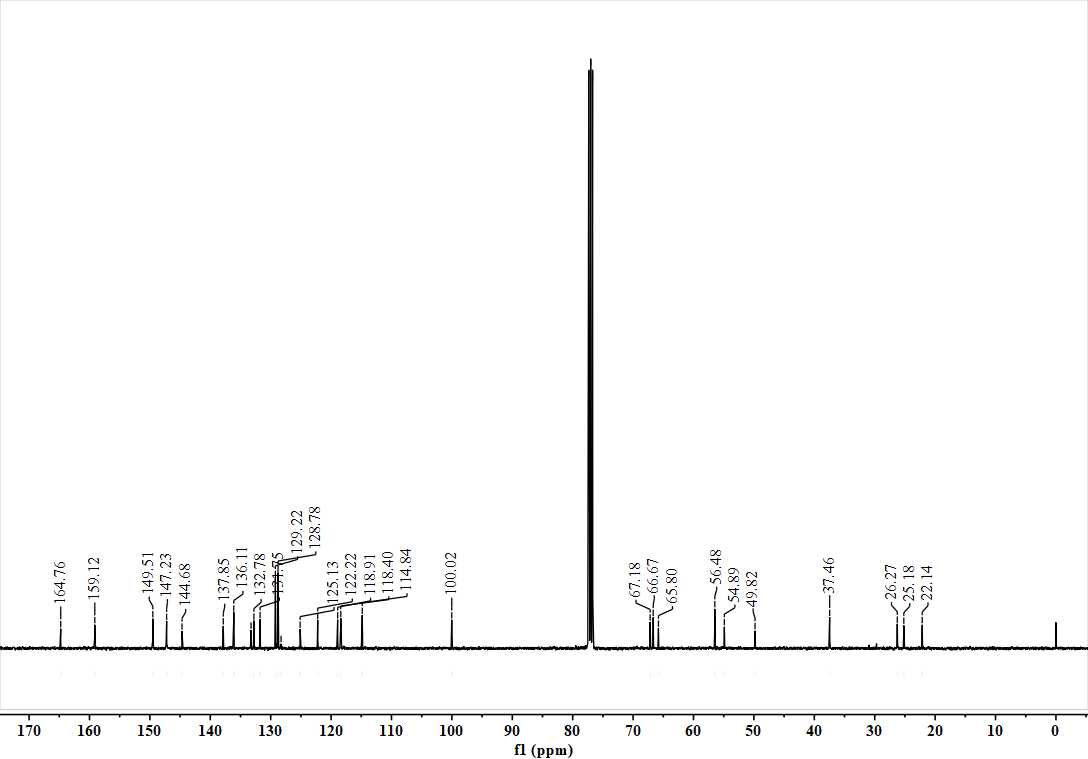


**Figure S45.** ^13^C-NMR of (1*S*,2*S*,4*S*,5*R*)-2-((*R*)-(cinnamoyloxy)(6-methoxyquinolin-4-yl)methyl)-1-methyl-5-vinylquinuclidin-1-ium iodide (**25**).
